# Interface-regulated vapour crystallization for ultrapure perovskite LEDs

**DOI:** 10.1038/s44160-026-01100-w

**Published:** 2026-06-22

**Authors:** Jian Mao, Qichun Gu, Yunzhou Deng, Milos Dubajic, Thomas A. Selby, Yorrick Boeije, Xinjuan Li, Yang Lu, Zhengkang Qu, Sebastiaan Hoek, Linfeng Pan, Weidong Xu, Tianjun Liu, Yuqi Sun, Yu Zhang, Benedetta Gaggio, Zimu Wei, Zher Ying Ooi, Yutong Han, Alessandro J. Mirabelli, Eunyoung Choi, Shenyu Nie, Yi Shen, Hayley Gilbert, Yuanle Tian, Xian Wei Chua, Joo Sung Kim, Xiaoliang Mo, Fengxian Xie, Jianlu Wang, Judith L. MacManus-Driscoll, Meikang Han, Junhao Chu, Neil C. Greenham, Henning Sirringhaus, Caterina Ducati, Tiarnan A. S. Doherty, Paul A. Midgley, Miguel Anaya, Samuel D. Stranks

**Affiliations:** 1 State Key Laboratory of Photovoltaic Science and Technology, Shanghai Frontiers Science Research Base of Intelligent Optoelectronics and Perception, College of Future Information Technology, Institute of Optoelectronics, Fudan University, Shanghai, China; 2 Department of Chemical Engineering and Biotechnology, University of Cambridge, Cambridge, UK; 3 Cavendish Laboratory, University of Cambridge, Cambridge, UK; 4 Department of Materials Science and Metallurgy, University of Cambridge, Cambridge, UK; 5 Diamond Light Source, Harwell Science and Innovation Campus, Oxfordshire, UK

**Keywords:** Electronic devices, Optical materials

## Abstract

Achieving ultranarrow spectral linewidth and broad spectral tunability in light-emitting diodes (LEDs) remains challenging due to linewidth broadening from compositional and size heterogeneities. Here we report an interface-regulated vapour crystallization strategy that enables precise control over the spectral linewidth of solution-processed halide perovskite thin films. Underlying materials that exhibit minimal molecular interactions with perovskite precursors, exemplified by poly(9-vinylcarbazole), facilitate smooth ion diffusion and crystallization assisted by dimethylformamide vapour. This mechanism leads to perovskite films with both horizontal and vertical homogeneity and low inhomogeneous broadening comparable to that of perovskite single crystals. We demonstrate perovskite films with ultranarrow photoluminescence linewidths of 13.6 nm, 13.7 nm, 13.8 nm and 14.4 nm for emissions at 464 nm, 474 nm, 483 nm and 522 nm, respectively. This enables us to achieve sky-blue perovskite LEDs with narrow electroluminescence linewidths of 14.7 nm and a peak external quantum efficiency of 24.6%, with comparable linewidths and performance in LEDs spanning the pure blue to pure green. This work offers a practical and scalable strategy to realize narrow spectral linewidth, broad spectral tunability and high performance in thin film LEDs.

**Figure:**
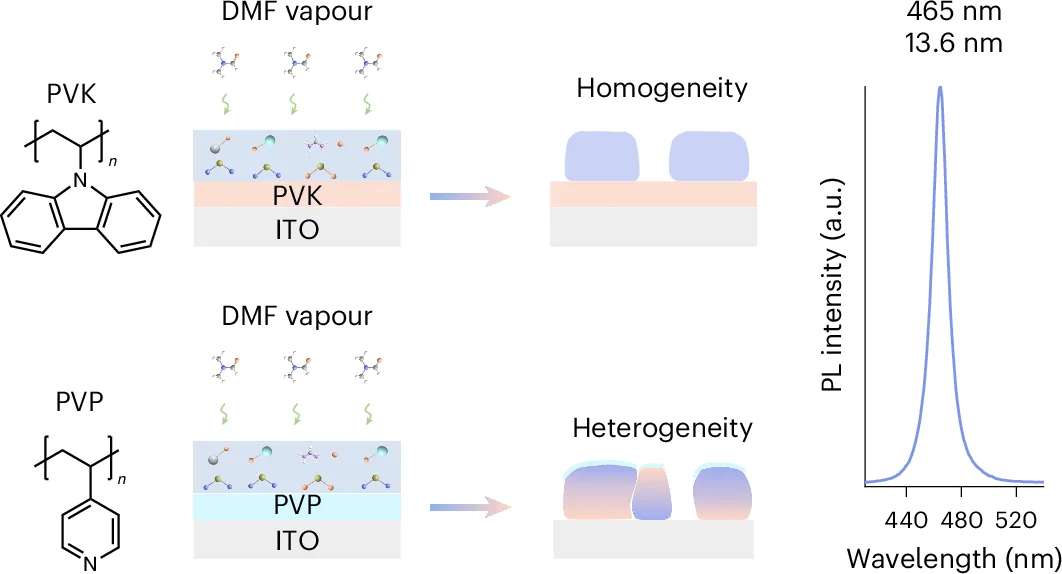

## Abstract (web summary)

An interface-regulated vapour crystallization strategy is reported that enables precise control over the spectral linewidth of solution-processed halide perovskite thin films. The demonstrated perovskite films have low inhomogeneous broadening and photoluminescence linewidths as low as 13.6 nm at 464 nm, enabling efficient perovskite light-emitting diodes spanning pure blue to pure green.

## Main

Precise control over the emission spectrum of semiconductors is fundamental to a broad range of technologies, including displays^1^, optical communications^2^, biomedical imaging^3^, optogenetics^4^ and quantum light sources^5^. However, simultaneously achieving narrow spectral linewidth and broad spectral tunability in semiconductor light-emitting diodes (LEDs) has remained a formidable challenge. III–V semiconductors (for example, GaN and its alloys) enable a wide spectral range from the ultraviolet (UV) to the infrared through compositional engineering. However, III–V LEDs^6^ typically exhibit a broad spectrum with full-width at half-maximum (FWHM) exceeding 150 meV, primarily caused by compositional and structural disorders as well as electron–phonon coupling. Comparable spectral tunability is achievable in chalcogenide quantum dots that leverage the quantum confinement effects but quantum dot LEDs^7–9^ generally show FWHMs above 100 meV, mainly limited by size heterogeneity and electron–phonon coupling. Organic semiconductors allow spectral tunability by modifying *π*-conjugation length or donor–acceptor character. Nevertheless, organic LEDs^10–13^ often display asymmetric emission profiles with long emission tails and broad FWHMs above 100 meV, largely due to structural relaxation and vibronic coupling.

Halide perovskites with the general formula ABX_3_ (where A = CH_3_NH_3_^+^, HC(NH_2_)_2_^+^ or Cs^+^; B = Pb^2+^ or Sn^2+^; X = I^−^, Br^−^ or Cl^−^) have garnered extensive attention over the past decade for their exceptional optoelectronic properties^14–16^. Their emission could be tuned continuously from violet to near-infrared via both quantum confinement effects and compositional engineering. However, these strategies often suffer from substantial inhomogeneous broadening, arising from size dispersity^17^ and compositional and structural inhomogeneity^18,19^. To approach the intrinsic linewidth limit in halide perovskite thin films and devices, these inhomogeneous broadenings must be minimized but doing so requires effective control over their crystallization.

Here we report an interface-regulated vapour crystallization strategy that enables fine control over the spectral linewidth of solution-processed perovskite thin films. We show that the interfacial chemistry between the underlying materials and perovskite precursors plays a critical role in regulating perovskite crystallization in the presence of dimethylformamide (DMF) vapour, ultimately determining its optical, structural and compositional homogeneity. Building on these insights, we demonstrate ultranarrow spectral linewidth for sky-blue-emitting perovskite thin films, which enables sky-blue LEDs with ultrahigh colour purity and high performance. Furthermore, we demonstrate the generality of this strategy by achieving comparable linewidths and performance in LEDs across the pure blue to pure green range.

## Results

**Fig. 1:**
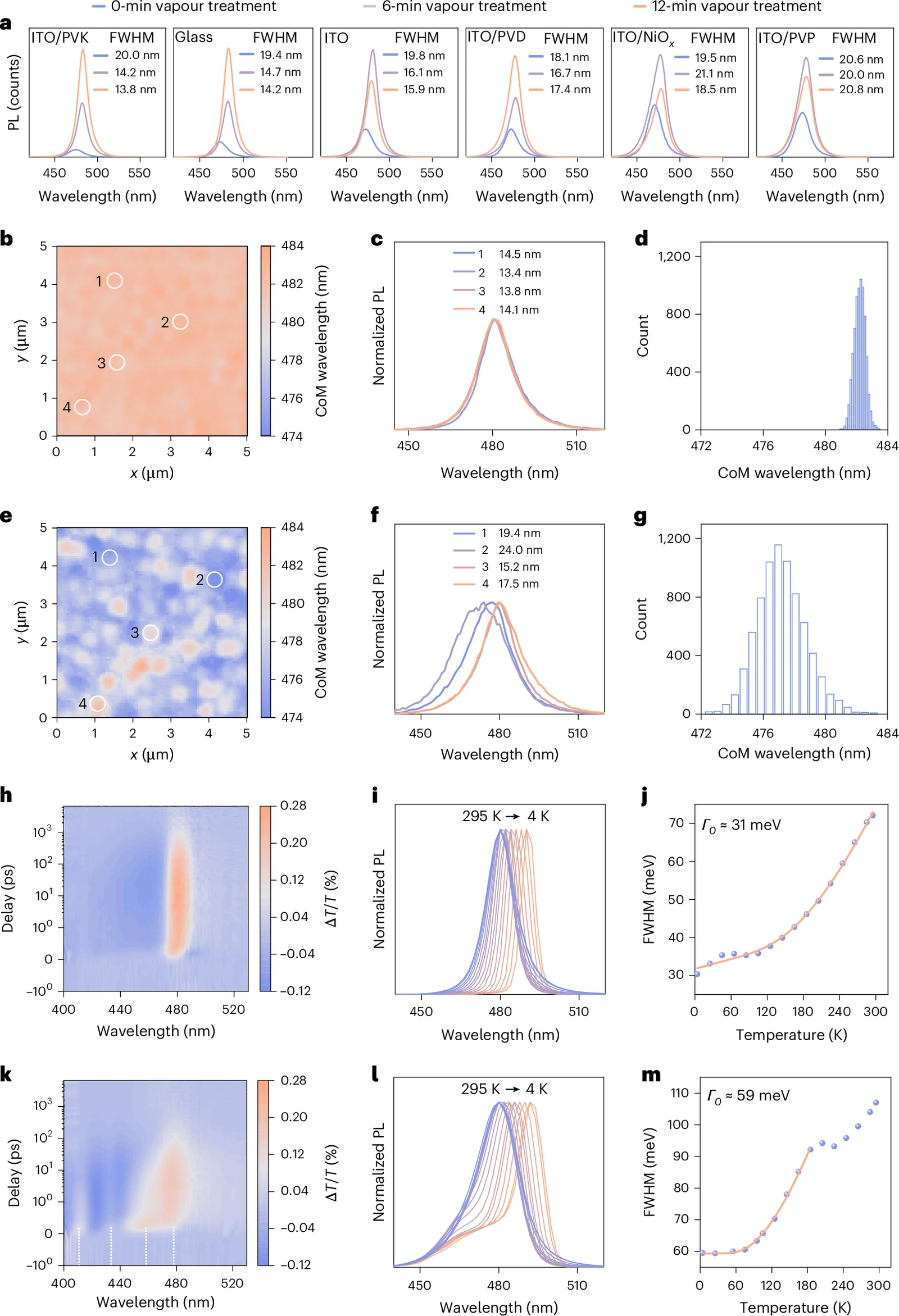
Underlying materials regulate spectral linewidths in vapour-treated perovskite thin films. **a**, PL spectra (excitation at 400 nm) of 35%Cl perovskite films deposited on ITO/PVK, bare glass, bare ITO, ITO/PVD, ITO/NiO_*x*_, ITO/PVP after DMF vapour treatment for 0 min (no treatment), 6 min or 12 min. **b**–**d**,**h**–**j**, Hyperspectral PL CoM peak map (**b**), PL spectra (**c**) of randomly selected four regions in **b**, CoM peak histogram (**d**) extracted from **b**, transient absorption contour (**h**), temperature-dependent PL spectra (**i**) and temperature-dependent FWHM (**j**) extracted from **i** of 35%Cl perovskite thin films on ITO/PVK after 12 min of DMF vapour treatment. **e**–**g**,**k**–**m**, Hyperspectral PL CoM peak map (**e**), PL spectra (**f**) of randomly selected four regions in **e**, CoM peak histogram (**g**) extracted from **e**, transient absorption contour (**k**), temperature-dependent PL spectra (**l**) and temperature-dependent FWHM (**m**) extracted from **l** of 35%Cl perovskite thin films on ITO/PVP after 12 min of DMF vapour treatment. The ITO/PVP, ITO/PVD and ITO/PVK films were prepared using 6 mg ml^−1^ PVP in IPA, 6 mg ml^−1^ PVD in IPA and 6 mg ml^−1^ PVK in CB, respectively. ITO/NiO_*x*_ film was prepared using commercial NiO_*x*_ nanocrystals after dilution with ethanol. The 35%Cl perovskite films were fabricated from precursors of RbBr:CsBr:FABr:PbBr_2_:PbCl_2_:TTDDA = 0.2:1.1:0.2:0.3875:0.6125:0.1. The concentration of perovskite precursors was fixed at 0.08 M Pb in DMSO.

**Extended Data Fig. 1:**
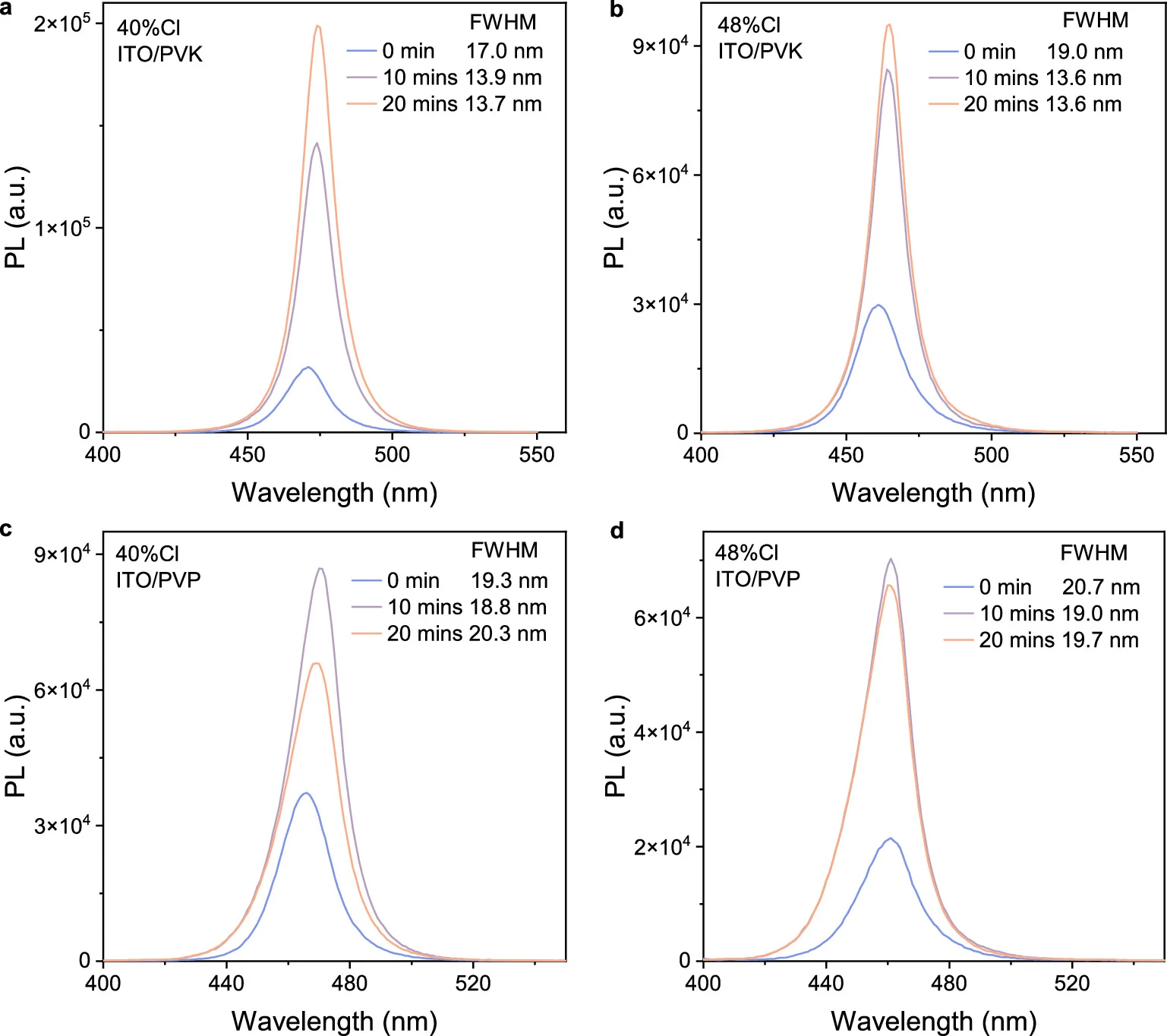
Underlying materials regulate spectral linewidths in vapor-treated 40%Cl and 48%Cl perovskite thin films. Photoluminescence (PL) spectra (excitation at 400 nm) of 40%Cl (**a**), and 48%Cl (**b**) perovskite films deposited on indium tin oxide (ITO)/poly(9-vinylcarbazole) (PVK) after dimethylformamide (DMF) vapor treatment for 0 minutes, 10 minutes, and 20 minutes. PL spectra (excitation at 400 nm) of 40%Cl (**c**), and 48%Cl (**d**) perovskite films deposited on ITO/poly(4-vinylpyridine) PVP after DMF vapor treatment for 0 minutes, 10 minutes, and 20 minutes. The 40%Cl and 48%Cl perovskite films were fabricated from precursors of RbBr:CsBr:FABr:PbBr_2_:PbCl_2_: 4,7,10-trioxa-1,13-tridecanediamine (TTDDA) = 0.2:1.1:0.2:*x*:1-*x*:0.1, where x = 0.30 and 0.16, respectively. The concentration of perovskite precursors was fixed at 0.08 M Pb in dimethyl sulfoxide (DMSO).

We deposited 35%Cl mixed-halide perovskite films (RbBr:CsBr:FABr:PbBr_2_:PbCl_2_:4,7,10-trioxa-1,13-tridecanediamine (TTDDA) = 0.2:1.1:0.2:0.3875:0.6125:0.1) onto different underlying materials, namely indium tin oxide (ITO)/poly(9-vinylcarbazole) (PVK), ITO/NiO_*x*_, ITO/poly(4-vinylpyridine) (PVP), ITO/polyvinylpyrrolidone (PVD), bare ITO and bare glass, followed by DMF vapour exposure for 0 minutes (no treatment), 6 minutes or 12 minutes. Unless otherwise noted, all films discussed in this work were thermally annealed at 80 °C before characterization. As shown in Fig. 1a, without DMF vapour treatment, the perovskite films exhibited similar photoluminescence (PL) spectra centred at ~470–475 nm with comparable FWHMs of ~18–21 nm. Notably, after DMF vapour treatment, we observed a pronounced dependence of the FWHM on the underlying materials. Specifically, after 6 minutes of vapour exposure, the FWHMs for perovskite films on ITO/PVK, bare glass, bare ITO, ITO/PVD, ITO/NiO_*x*_ and ITO/PVP were 14.2 nm, 14.7 nm, 16.1 nm, 16.7 nm, 21.1 nm and 20.0 nm, respectively. After 12 minutes of vapour treatment, the FWHMs further evolved to 13.8 nm, 14.2 nm, 15.9 nm, 17.4 nm, 18.5 nm and 20.8 nm, respectively. These results indicate that for a given posttreatment duration, the FWHM is strongly influenced by the underlying materials, whereas extending the vapour exposure time only slightly tunes the FWHM once the underlying materials are fixed. Among these underlying materials investigated, we are particularly interested in ITO/PVK and ITO/PVP considering that the former yielded the narrowest FWHM (13.8 nm, 73 meV) while the latter led to the broadest FWHMs (20–21 nm, 108–114 meV) and an asymmetric emission profile even for extended vapour treatment (Supplementary Fig. 1b). This underlying material-dependent behaviour is not restricted to 35%Cl perovskite, but is also observed for perovskite with higher chloride content. As shown in Extended Data Fig. 1a,b, the FWHMs for 40%Cl and 48%Cl perovskite films on ITO/PVK after 20 minutes of vapour treatment reduced to 13.7 nm (76 meV) and 13.6 nm (78 meV), respectively. In contrast, the FWHMs for 40%Cl and 48%Cl perovskite on ITO/PVP after 20 minutes of vapour treatment were 20.3 nm (114 meV) and 19.7 nm (115 meV), respectively, as displayed in Extended Data Fig. 1c,d. Although the DMF vapour treatment has been investigated previously^19–21^, the critical role of the underlying layers in governing the FWHM of perovskite thin films is not well understood. More critically, blue-emitting thin films with such narrow spectral linewidths at room temperature (13.6 nm at 464 nm, 13.7 nm at 474 nm and 13.8 nm at 483 nm) without emission tails have not been achieved with III–V semiconductors^22,23^, chalcogenide quantum dots^7,8,24,25^, organic semiconductors^12,13,26^ or halide perovskite^1,20,27^ as the emissive layers.

**Extended Data Fig. 2:**
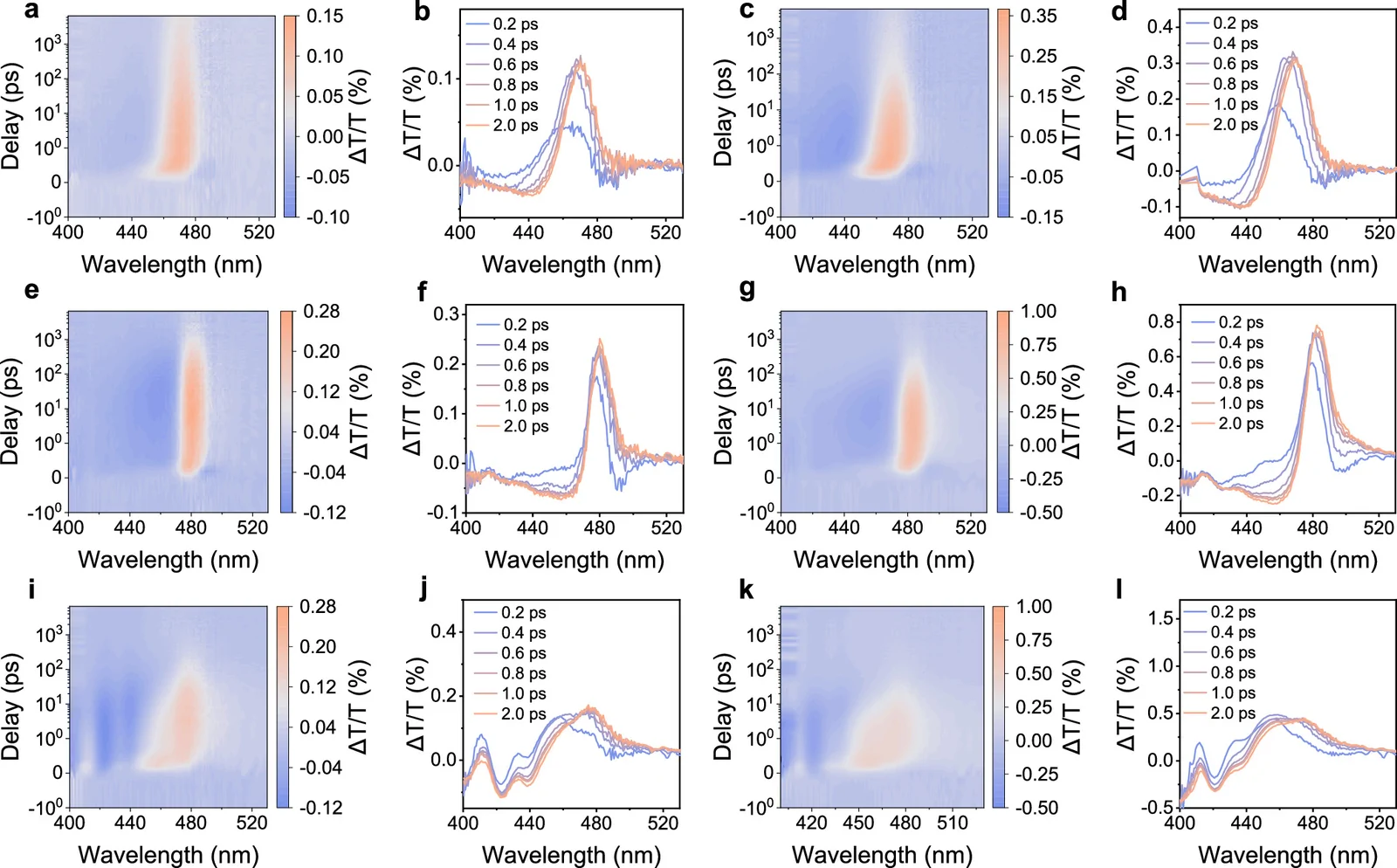
Transient absorption spectroscopy. Transient absorption map and spectra of 35%Cl perovskite on indium tin oxide (ITO)/poly(9-vinylcarbazole) (PVK) without dimethylformamide (DMF) vapor treatment under an excitation fluence of 1.8 μJ cm^−2^ pulse^−1^ (**a**, **b**) and 5.8 μJ cm^−2^ pulse^−1^ (**c**, **d**). Transient absorption map and spectra of 35%Cl perovskite on ITO/PVK after 12 minutes of post-treatment under an excitation fluence of 2.0 μJ cm^−2^ pulse^−1^ (**e**, **f**) and 8.1 μJ cm^−2^ pulse^−1^ (**g**, **h**). Transient absorption map and spectra of 35%Cl perovskite on ITO/poly(4-vinylpyridine) (PVP) after 12 minutes of post-treatment with an excitation fluence of 6.8 μJ cm^−2^ pulse^−1^ (**i**, **j**) and 35.2 μJ cm^−2^ pulse^−1^ (**k**, **l**).

**Extended Data Fig. 3:**
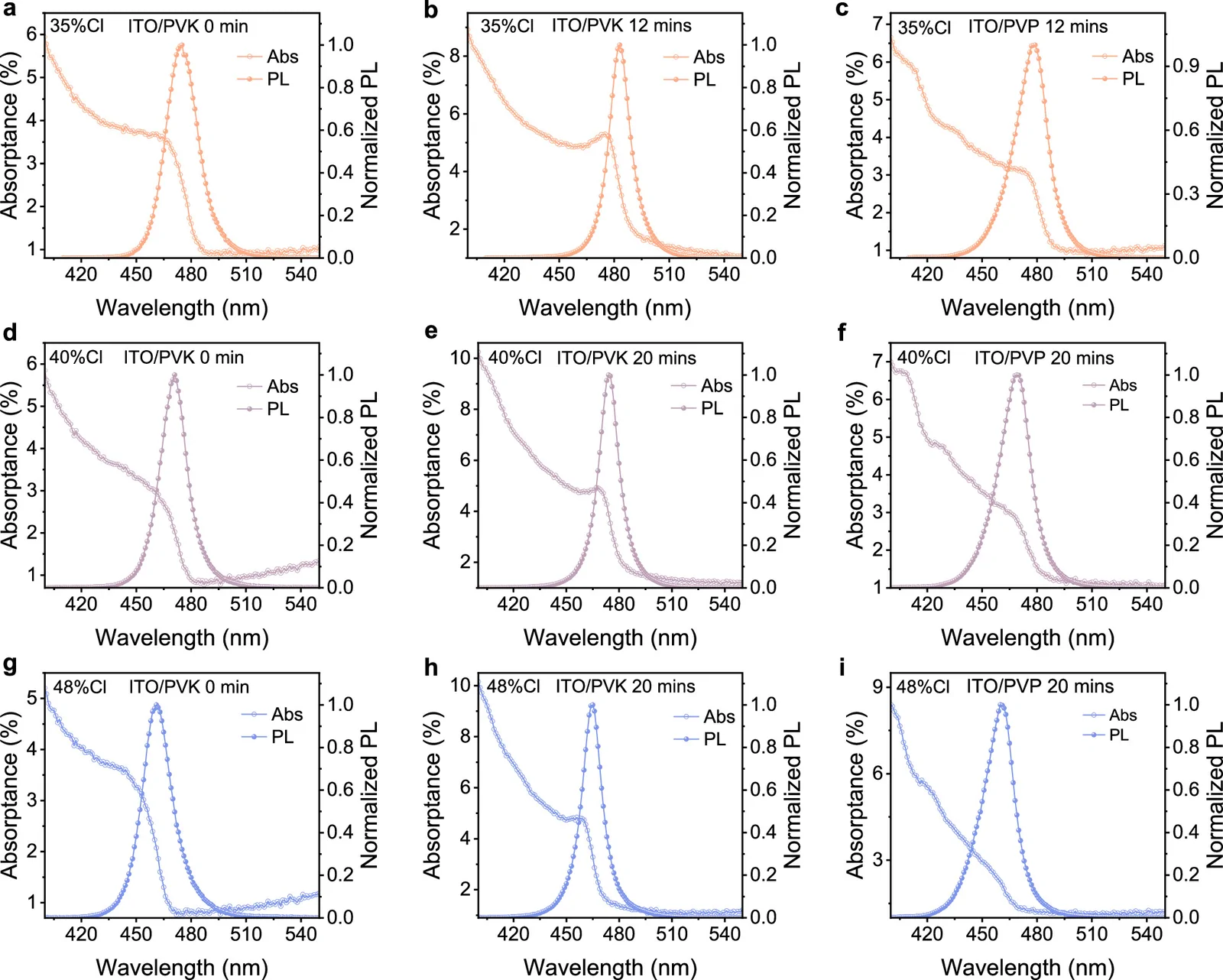
Absorptance and photoluminescence of 35%Cl, 40% and 48%Cl perovskite films. Absorptance and photoluminescence (PL) spectrum of 35%Cl (**a**), 40%Cl (**d**), and 48%Cl (**g**) perovskite on indium tin oxide (ITO)/poly(9-vinylcarbazole) (PVK) without dimethylformamide (DMF) vapor treatment. Absorptance and PL spectrum of 35%Cl (**b**), 40%Cl (**e**), and 48%Cl (**h**) perovskite on ITO/PVK after post-treatment for 12 minutes, 20 minutes, and 20 minutes, respectively. Absorptance and PL spectrum of 35%Cl (**c**), 40%Cl (**f**), and 48%Cl (**i**) perovskite on ITO/poly(4-vinylpyridine) (PVP) after post-treatment for 12 minutes, 20 minutes, and 20 minutes, respectively.

To elucidate the origin of the distinct FWHMs, we performed hyperspectral widefield PL microscopy, transient absorption spectroscopy and cryogenic PL measurements on 35%Cl perovskite thin films deposited on ITO/PVK and ITO/PVP after 12 minutes of vapour treatment. The hyperspectral PL centre-of-mass (CoM) peak map for posttreated perovskite on ITO/PVK is shown in Fig. 1b, with representative PL spectra from four randomly selected regions and the corresponding CoM histogram presented in Fig. 1c,d, respectively. The selected regions exhibited nearly identical PL spectra with narrow FWHMs of 13–14 nm, closely matching the macroscopic PL linewidth in Fig. 1a. The CoM peaks were centred at 482.2 ± 1.3 nm, where the error represented the variation in value, indicating outstanding film homogeneity. It is worth noting that vapour-treated perovskite films exhibited spatially isolated grains (Supplementary Note 1). These results indicate that the narrow linewidth observed in the posttreated perovskite films on ITO/PVK originates from both intrinsically narrow emission from individual grains and minimal grain-to-grain spectral variation. In contrast, the posttreated perovskite films on ITO/PVP exhibited pronounced heterogeneity. As shown in Fig. 1f, the PL spectra from four randomly selected regions revealed diverse peak positions ranging from ~474 nm to 480 nm with broad FWHMs of ~15–24 nm. The CoM peak distribution was correspondingly broader (Fig. 1e), centred at 477.2 ± 5.0 nm (Fig. 1g), suggesting a higher degree of inhomogeneity. These differences in film homogeneity are further corroborated by transient absorption spectroscopy. The posttreated perovskite films on ITO/PVK exhibited a single sharp ground-state bleach feature centred at ~480 nm with a negligible spectral shift over time (Fig. 1h). In contrast, posttreated perovskite films on ITO/PVP displayed multiple ground-state bleach features at ~410 nm, 433 nm, 460 nm and 477 nm (Fig. 1k), independent of excitation fluence (Extended Data Fig. 2e–h). In addition, these multiple ground-state bleach features are consistent with the shoulders at these wavelengths in the absorptance spectrum in Extended Data Fig. 3c. The less pronounced excitonic absorption peak in posttreated perovskite on ITO/PVP than that on ITO/PVK (Extended Data Fig. 3b) could be due to inhomogeneity-induced obscuration of the excitonic feature (Supplementary Note 2).

To quantitatively assess the degree of inhomogeneity, we conducted temperature-dependent PL measurements. On cooling from 295 K to 4 K, the PL peak of posttreated perovskite films on ITO/PVK underwent a continuous redshift from 480 nm to 490 nm (Fig. 1i), accompanied by a gradual narrowing of the FWHM from 13.4 nm (72.1 meV) to 5.9 nm (30.3 meV). This redshift with decreasing temperature is characteristic of lead halide perovskite^18,28–30^. In contrast, posttreated perovskite films on ITO/PVP exhibited distinct spectral splitting at low temperatures (Fig. 1l), although the dominant peak was still redshifted. The FWHM of perovskite thin films on ITO/PVP decreased from 19.8 nm (107 meV) at 295 K to 11.6 nm (59.5 meV) at 4 K. Fitting the temperature-dependent FWHM^28,29^ (Methods) allows us to extract the inhomogeneous broadening term *Γ*_0_, longitudinal optical (LO) phonon energy *E*_LO_ and carrier-longitudinal optical phonon coupling strength *γ*_LO_. The extracted *Γ*_0_ value was 31.7 ± 0.6 meV and 59.4 ± 0.3 meV for posttreated perovskite films on ITO/PVK and ITO/PVP, respectively. The posttreated perovskite films on ITO/PVK exhibited ~30 meV lower inhomogeneous broadening compared with that on ITO/PVP and ~25 meV lower than reported mixed-halide perovskite blue-emitting thin films^18^. Notably, the inhomogeneous broadening achieved here is comparable to that of CsPbBr_3_ (~34 meV)^29^, CH_3_NH_3_PbBr_3_ (~32–33 meV)^31,32^ and CsPbCl_3_ (~29 meV)^29^ single crystals, indicating that the posttreated 35%Cl perovskite films on ITO/PVK approach single-crystal-like optical quality. Meanwhile, the extracted *E*_LO_ are 49.1 ± 1.4 meV and 32.5 ± 1.4 meV for the vapour-treated perovskite films on ITO/PVK and ITO/PVP, respectively. The corresponding *γ*_LO_ are 216.3 ± 14.4 meV and 222.5 ± 23.8 meV, respectively. Consistent with reports on two-monolayer perovskite nanoplatelets, a higher optical phonon energy can reduce phonon population and thus thermal broadening of PL spectra^33^. Therefore, the narrower spectral linewidth of the vapour-treated perovskite on ITO/PVK arises primarily from its substantially smaller inhomogeneous broadening, while the higher optical phonon energy may further suppress thermal broadening by reducing the phonon population.

**Extended Data Fig. 4:**
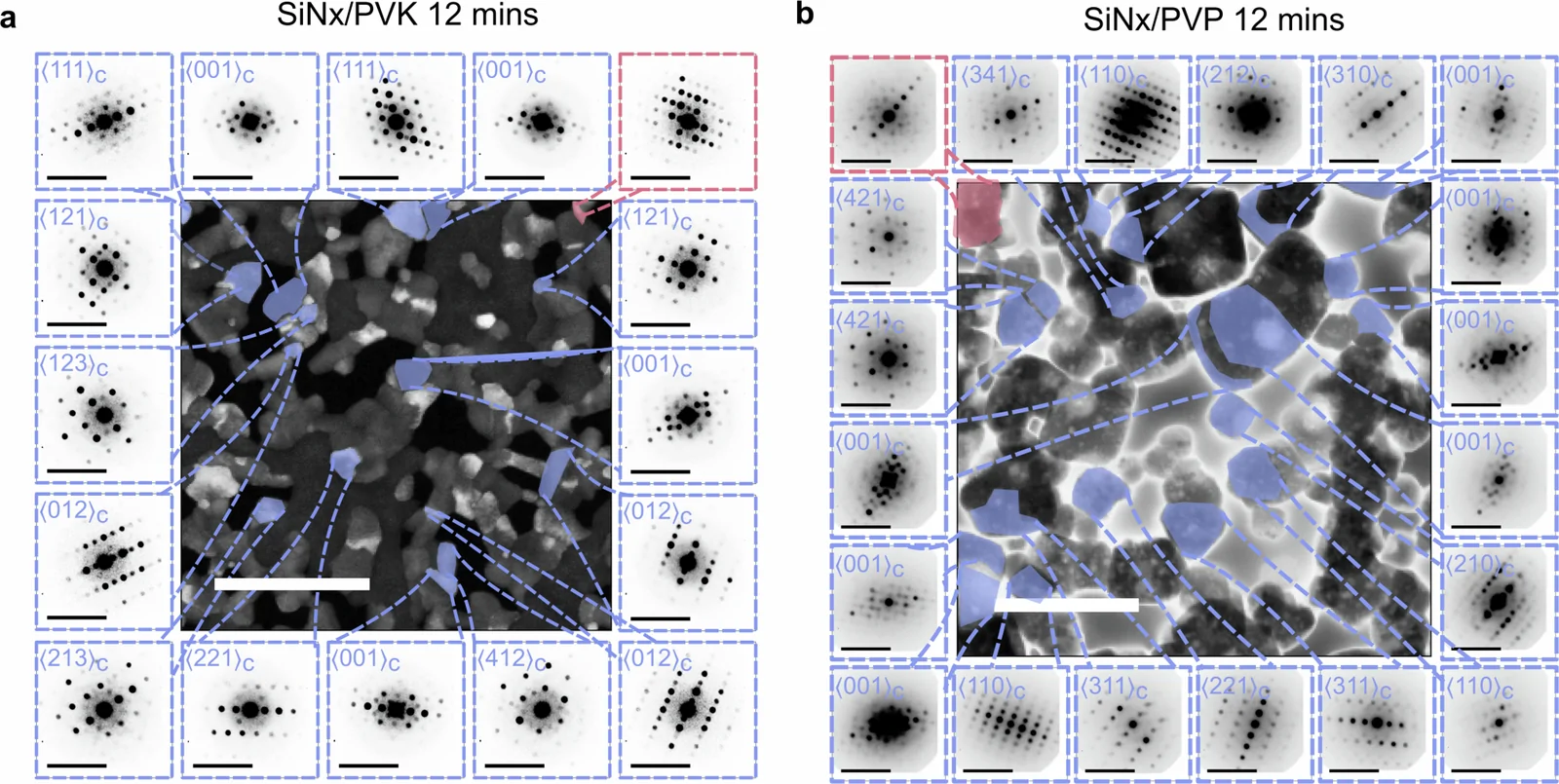
Scanning Electron Diffraction (SED) of vapor-treated perovskite on SiN_*x*_/PVK and SiN_*x*_/PVP. Virtual darkfield image reconstructed from the SED results in 35%Cl perovskite on SiN_*x*_/poly(9-vinylcarbazole) (PVK) (**a**) and SiN_*x*_/poly(4-vinylpyridine) (PVP) (**b**) after 12 minutes of vapor treatment. 6 mg/ml PVK or PVP was spin-coated on standard SiN_*x*_ window grids, followed by spin-coating of perovskite and vapor exposure for 12 minutes. Around the image are diffraction patterns indexed to an approximate zone axis using a pseudo-cubic unit cell (blue), and overlapping grains or phase impurities that cannot be indexed to a pseudo-cubic unit cell (red). Scale bars in a are 300 nm (inner) and **s** = 1 Å^−1^ (outer) and those in b are 1 µm (inner) and **s** = 1 Å^−1^ (outer). The electron fluence (Df) was ~11 e^−^ Å^−2^ and ~22 e^−^ Å^−2^ for **a** and **b**, respectively. In both cases, most grains were indexed to a corner-sharing pseudo-cubic phase (Pm3^−^m, a = 5.76 Å), though a small fraction of diffraction patterns (highlighted in red) could not be indexed to the pseudo-cubic structure, potentially arising from overlapping grains or minor phase impurities. Note that the use of a pseudo-cubic unit cell to index electron diffraction patterns is due to not precisely knowing the octahedral tilting scheme for our material. Indeed, this is likely ill-defined due to subtle structural modulations (such as octahedral tilting) originating from compositional heterogeneity within the film. We note that the use of the pseudo-cubic cell is consistent with that used to index grazing-incidence wide-angle X-ray scattering (GIWAXS) patterns in the main text.

To gain deeper insight into the distinct inhomogeneity, we investigated the morphological, crystallographic and compositional characteristics of 35%Cl perovskite on ITO/PVK (SiN_*x*_/PVK) and ITO/PVP (SiN_*x*_/PVP) using scanning electron microscopy (SEM), scanning electron diffraction (SED), grazing-incidence wide-angle X-ray scattering (GIWAXS) and time-of-flight secondary ion mass spectrometry (TOF-SIMS). SEM images in Supplementary Fig. 2 revealed isolated and increased perovskite grains on vapour treatment for films on both ITO/PVK and ITO/PVP. However, the perovskite on ITO/PVP crystallized more slowly and formed flower-like clusters, a feature also observed in posttreated 48%Cl on ITO/PVP (Supplementary Fig. 3b). These results indicate an underlying material-dependent morphological response to vapour treatment (Supplementary Note 1). To gain a crystallographic understanding of the isolated perovskite grains, we performed SED measurement^34^ on vapour-treated perovskite on SiN_*x*_/PVK and SiN_*x*_/PVP. Hyperspectral PL measurement (Supplementary Fig. 4) confirmed that vapour-treated perovskite on SiN_*x*_/PVK exhibited narrower and symmetric PL spectra than that on SiN_*x*_/PVP, consistent with the observations for films on ITO/PVK and ITO/PVP. As shown in Extended Data Fig. 4a and Supplementary Fig. 5, the perovskite grains grown on SiN_*x*_/PVK exhibited sharp and well-defined diffraction patterns, indicating a good crystallinity of each grain. In contrast, the perovskite grains grown on SiN_*x*_/PVP were substantially larger and thicker with pronounced intra-grain heterogeneity, as clearly evidenced by both the virtual dark-field image (Extended Data Fig. 4b) and the high-angle annular dark-field (HAADF) image (Supplementary Fig. 6b) where intra-grain voids are visually present. In addition, compositional variation laterally across grains manifests at smaller length scales in the energy-dispersive X-ray (EDX) mapping (Supplementary Fig. 7k).

**Fig. 2:**
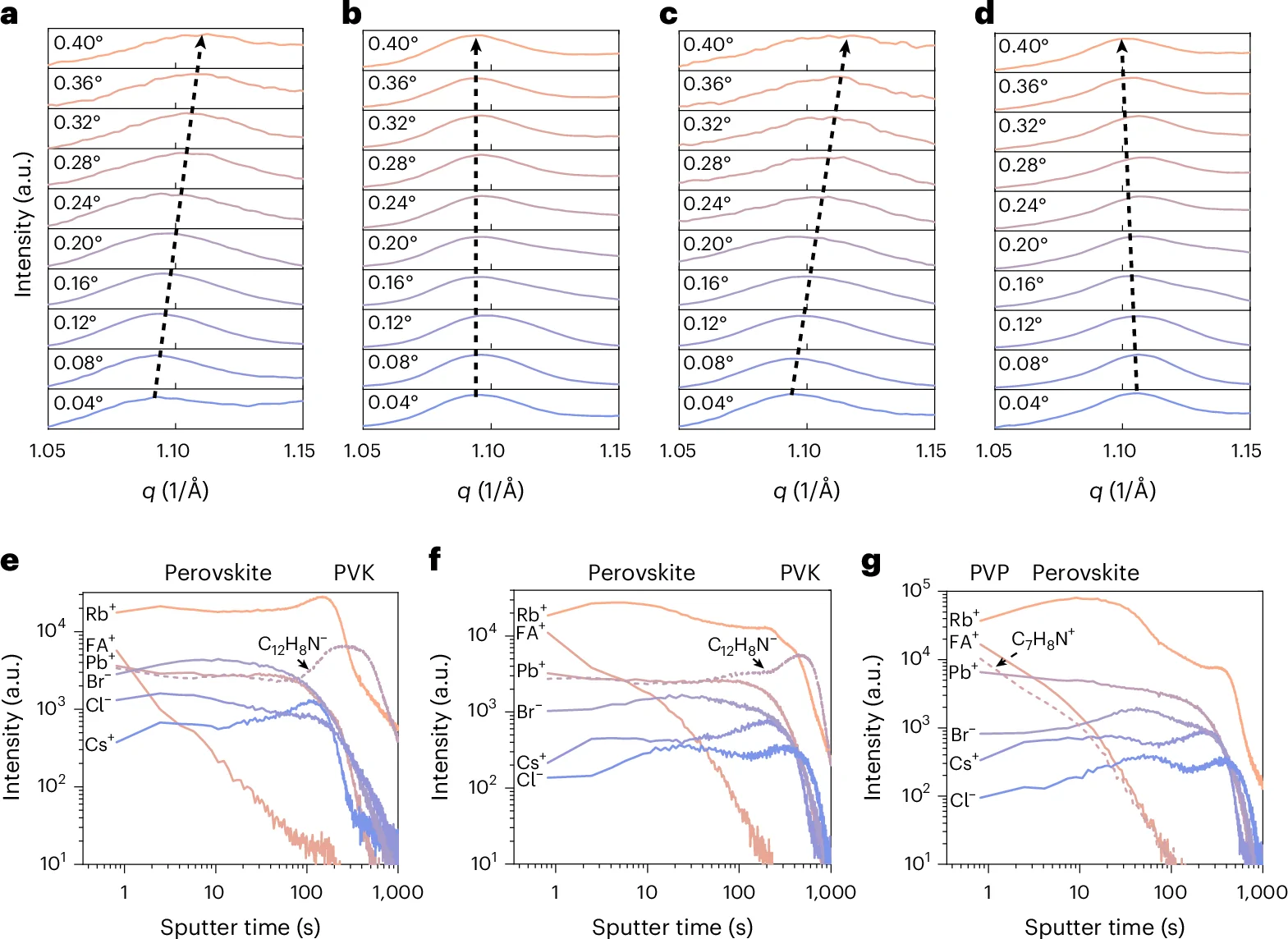
Underlying materials regulate structural and compositional homogeneity in vapour-treated perovskites. **a**–**d**, Incident angle-dependent two-dimensional (2D) GIWAXS plot of 35%Cl perovskite on ITO/PVK thin films after DMF vapour treatment for 0 min (**a**) and 12 min (**b**), as well as on ITO/PVP after DMF vapour treatment for 0 min (**c**) and 12 min (**d**). These curves are extracted by integrating their intensities between 3° and 90° in Supplementary Figs. 8 and 9. The diffraction peak with a *q* value of ~1.10 Å^−1^ corresponds to the (101) plane of perovskite. **e**–**g**, TOF-SIMS depth profiles of 35%Cl perovskite thin films on ITO/PVK after DMF vapour treatment for 0 minutes (**e**) and 12 min (**f**) as well as on ITO/PVP after DMF vapour treatment for 12 min (**g**). The C_7_H_8_N^+^ is from PVP and the C_12_H_8_N^−^ is from PVK. The labels perovskite, PVK and PVP indicate the relative position of these layers.

These differences in nanoscale crystallographic homogeneity were further corroborated by incident angle-dependent GIWAXS measurements on perovskite films deposited on ITO/PVK and ITO/PVP. We focus on the primary diffraction peak located at a *q* value of ~1.100 Å^−1^ that corresponds to the (101) plane of perovskite (*Pnma*, *a* = 8.131 Å, *b* = 11.574 Å, *c* = 8.099 Å)^35,36^ because other primary peaks (for example, at ~1.550 Å^−1^ and ~2.170 Å^−1^) overlapped with those from the ITO substrate and thus exhibited peak distortion at higher incident angles (Supplementary Fig. 8 and 9). For the perovskite films grown on ITO/PVK without vapour treatment (Fig. 2a), the (101) diffraction peak exhibited a gradual shift from ~1.091 Å^−1^ (corresponding to a *d*-spacing of ~5.759 Å) at a small incident angle (0.04°) to ~1.112 Å^−1^ ( ~ 5.650 Å) at a larger incident angle (0.4°). The corresponding X-ray attenuation depth was estimated to be ~2 nm (0.04°) to ~109 nm (0.4°), which is tabulated in Supplementary Table 1. These findings reveal a pronounced vertical crystallographic inhomogeneity in perovskite films without vapour treatment. On 6 minutes (Supplementary Fig. 10a) and 12 minutes (Fig. 2b) of vapour treatment, the (101) diffraction peak aligned well at ~1.095 Å^−1^ (~5.738 Å) across the incident angles investigated, indicative of a more vertically uniform crystal structure. For the perovskite films grown on ITO/PVP without vapour treatment (Fig. 2c), we also observed a vertical crystallographic inhomogeneity, as evidenced by the variable (101) diffraction peak positions with changing incident angles. After DMF vapour treatment for 6 minutes (Supplementary Fig. 10b) and 12 minutes (Fig. 2d), a misalignment between the diffraction peaks at small and large incident angles remained, indicating a vertical crystallographic inhomogeneity. Furthermore, the extracted FWHM values of the (101) plane (Supplementary Fig. 10c) in posttreated perovskite films on ITO/PVP are consistently broader than those on ITO/PVK across the incident angles, indicating higher structural disorders in the former.

**Extended Data Fig. 5:**
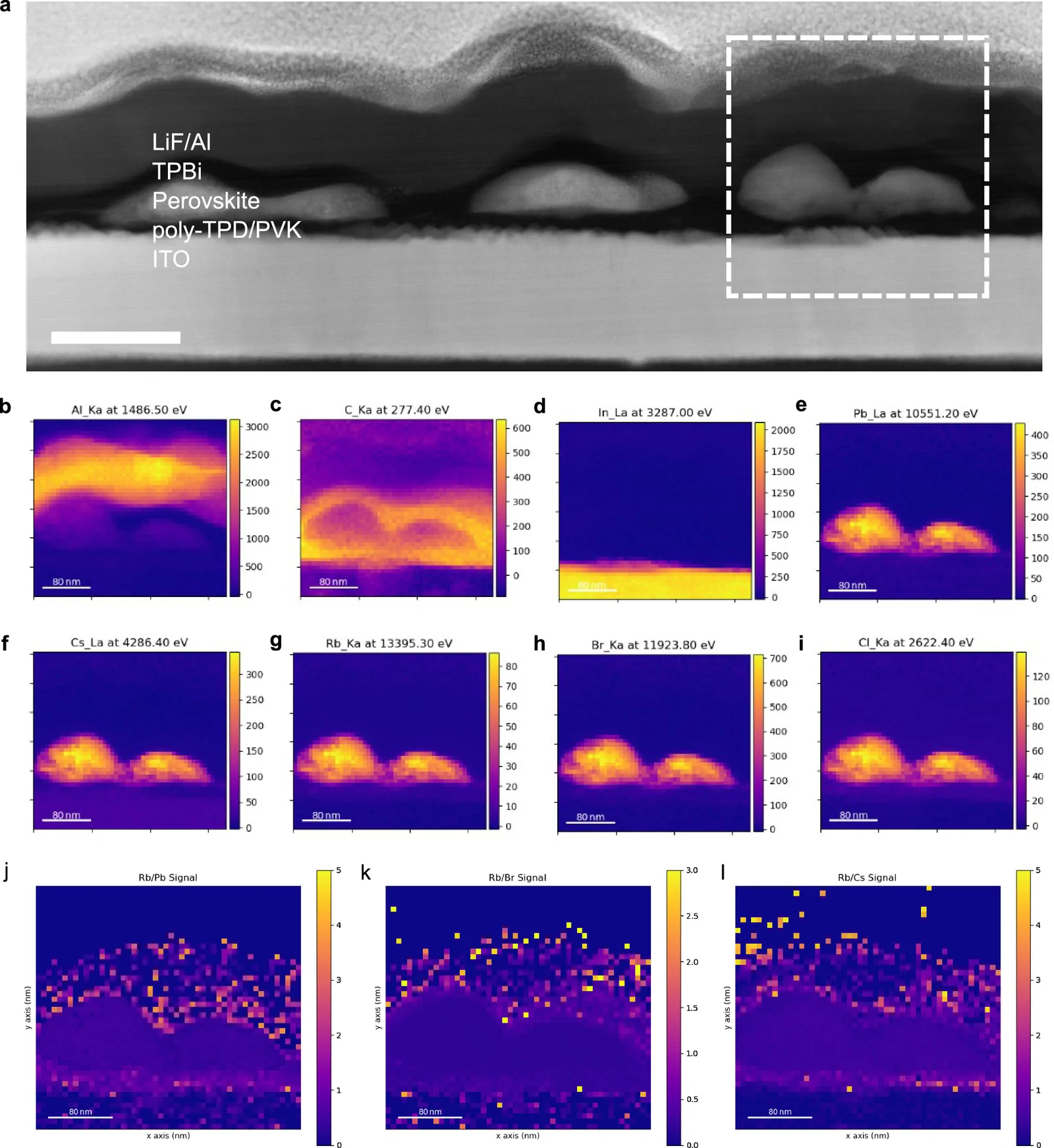
HAADF-STEM-EDX micrographs of 35%Cl PeLEDs. **a**, Cross-section high-angle annular dark-field scanning transmission electron microscopy (HAADF-STEM) micrographs of 35%Cl perovskite light-emitting diodes (PeLEDs) with a structure of indium tin oxide (ITO)/poly(N,N’-bis-4-butylphenyl-N,N’-bisphenyl)benzidine (poly-TPD)/poly(9-vinylcarbazole) (PVK)/perovskite/2,2’,2’-(1,3,5-benzinetriyl)-tris(1-phenyl-1-H-benzimidazole) (TPBi)/LiF/Al. The scale bar is 150 nm. Energy-dispersive X-ray spectroscopy (EDX) maps of the dotted area in (**a**) based on elements Al (**b**), C (**c**), In (**d**), Pb (**e**), Cs (**f**), Rb (**g**), Br (**h**), Cl (**i**), Rb/Pb ratio (**j**), Rb/Br ratio(**k**), and Rb/Cs ratio (**l**). The scale bar is 80 nm. The EDX data was denoised with principal component analysis and non-matrix factorization.

This different crystallographic inhomogeneity is further supported by TOF-SIMS depth profiles. As shown in Fig. 2e, perovskite thin films deposited on ITO/PVK without vapour exposure exhibited slight surface enrichment of Br^−^ and Cl^−^, along with pronounced accumulation of FA^+^ near the surface and Rb^+^ and Cs^+^ at the bottom. Given the larger ionic radius of FA^+^ (253 pm) compared with Cs^+^ (167 pm) and Rb^+^ (152 pm)^37^, this vertical compositional gradient is consistent with the broader *d*-spacing of the (101) plane near the surface and narrower *d*-spacing at greater depths in the angle-dependent GIWAXS (Fig. 2a). Note that a previous study^38^ on solution-processed FA_1-x_Cs_x_PbI_3_ thin films showed that FA^+^ preferentially accumulated near the film surface, whereas Cs^+^ was more concentrated towards the bottom, which was attributed to the unbalanced crystallization and phase transitions between A-site components. In contrast, on 12 minutes vapour exposure, the perovskite films on ITO/PVK exhibited improved vertical homogeneity for Br^−^, Cl^−^ and Cs^+^, while FA^+^ and Rb^+^ remained enriched at the film surface (Fig. 2f). This observation is further corroborated with the cross-sectional HAADF scanning transmission electron microscopy (STEM) coupled with EDX spectroscopy (HAADF-STEM-EDX) that revealed slightly elevated Rb/Pb (Extended Data Fig. 5j), Rb/Br (Extended Data Fig. 5k) and Rb/Cs (Extended Data Fig. 5l) ratios at the surface relative to the bulk. The simultaneous enrichment of large radius FA^+^ and small radius Rb^+^ at the surface may effectively tune the local lattice constant, resulting in uniform *d*-spacing of (101) plane throughout the film thickness (Fig. 2b). Nevertheless, despite a similar surface accumulation of FA^+^ and Rb^+^ in posttreated perovskite on ITO/PVP, these films exhibited greater vertical inhomogeneity for Rb^+^, Br^−^ and Cl^−^ (Fig. 2g). This correlates well with the misalignment of (101) plane throughout the film thickness (Fig. 2d).

**Fig. 3:**
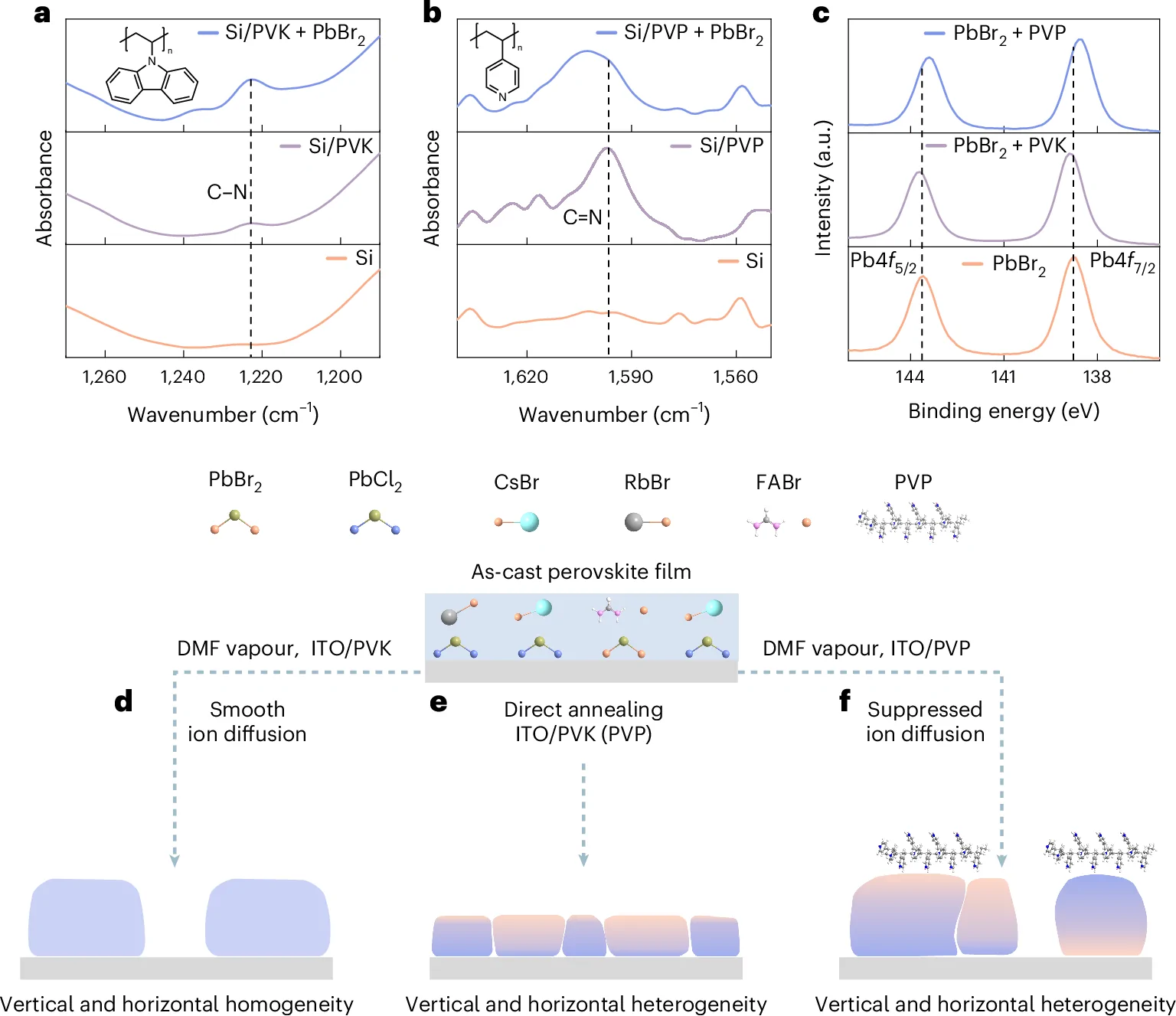
Mechanism of underlying materials-regulated vapour crystallization. **a**, FTIR of PVK and PVK + PbBr_2_ thin films on Si. The inset shows the molecular structure of PVK. **b**, FTIR spectra of PVP and PVP + PbBr_2_ thin films on Si. The inset shows the molecular structure of PVP. **c**, XPS Pb4*f* spectra of PbBr_2_, PbBr_2_ + PVK, and PbBr_2_ + PVP thin films on ITO. The XPS spectra were calibrated using its C1s peak (binding energy of 284.8 eV). **d**–**f**, Schematic images of perovskite crystallized on ITO/PVK after vapour exposure (**d**), on ITO/PVK or ITO/PVP after direct thermal annealing (**e**) and on ITO/PVP after vapour exposure (**f**). The colour scale represents compositional variation.

To elucidate the underlying materials-regulated inhomogeneity from the perspective of molecular interactions, we performed Fourier-transform infrared spectroscopy (FTIR) and X-ray photoelectron spectroscopy (XPS) measurements on PbBr_2_ mixed with PVK or PVP. The FTIR spectra (Fig. 3a) of pristine PVK and its mixture with PbBr_2_ showed no discernible shift or broadening in the C–N stretching vibration mode at ~1,222 cm^−1^, a characteristic signature of PVK^39^. This observation suggests minimal interaction between PVK and PbBr_2_. In contrast, the FTIR spectra (Fig. 3b) of PVP and its mixture with PbBr_2_ revealed a distinct shift in the C=N stretching vibration^40^ from ~1,596 cm^−1^ to approximately 1,603 cm^−1^ on mixing. This redshift is indicative of coordination between the lone pair electrons on the C=N group in PVP and the empty 6*p* orbital of Pb^2+^, consistent with pyridine-metal complex formation^41^. Apart from that, the molecular interaction between PbBr_2_ and PVP is further confirmed by the XPS result. As shown in Fig. 3c, the Pb4*f*_7/2_ peak shifted from 138.7 eV in pristine PbBr_2_ to 138.5 eV in PbBr_2_ mixed with PVP, accompanied by a corresponding shift of the Pb4*f*_5/2_ peak from 143.6 eV to 143.4 eV. The decrease in binding energy signifies electron donation from PVP to Pb^2+^, consistent with reported perovskite doped with TFPPO^42^. In contrast, mixing PbBr_2_ with PVK led to a minimal shift towards high binding energy (Fig. 3c), supporting the conclusion of a weak interaction between PVK and the Pb4*f* orbital of PbBr_2_.

Apart from the molecular interaction, we find that the C=N stretching vibration of PVP disappeared in posttreated perovskite films on Si/PVP (Supplementary Fig. 11). Even simply spin-coating dimethyl sulfoxide (DMSO) onto Si/PVP also led to the disappearance of this vibration, suggesting that the PVP dissolves in DMSO during perovskite fabrication. Dissolved PVP remained in perovskite thin films with a limited amount and accumulated at the perovskite surface, as evidenced by TOF-SIMS, SED and conductive atomic force microscopy (c-AFM) analysis. In Fig. 2g, the C_7_H_8_N^+^ signals attributed to PVP^43^ were primarily located near the film surface, in contrast to the C_12_H_8_N^−^ signals from PVK^44^ that remained concentrated underneath perovskite both before and after vapour exposure. In addition, SED mapping (Supplementary Fig. 7a) revealed a thin amorphous layer surrounding the perovskite grains deposited on SiN_*x*_/PVP, which was absent in the SiN_*x*_/PVK counterpart (Supplementary Fig. 5). Furthermore, c-AFM measurements (Supplementary Fig. 12e,f) showed a pronounced increase in conductivity on ITO/PVP following perovskite deposition and vapour treatment, rising from the noise level (30–40 pA) to several nanoamps under a 3-V bias.

We propose a mechanistic framework to elucidate how the underlying materials regulate the spectral linewidths of mixed-composition perovskite thin films before and after DMF vapour treatment. Initially, as-cast perovskite films exhibit negligible PL intensity (Supplementary Fig. 13), primarily due to the presence of residual DMSO that suppresses crystallization. Direct thermal annealing of the as-cast films leads to perovskite crystallization but this process results in vertical (Fig. 2a,e) and horizontal heterogeneity (Supplementary Fig. 14), as illustrated in Fig. 3e. This heterogeneity, largely independent of the underlying materials (Fig. 2a,c), probably originates from a top-to-bottom crystallization process driven by the gradual evaporation of the residual DMSO from the film surface^45^, as well as unbalanced crystallization and phase transition^38^. This heterogeneity explains the broad FWHM (Fig. 1a) in perovskite films via direct thermal annealing. In contrast, DMF vapour exposure induces a different crystallization pathway. On DMF vapour treatment for 1 minute, the as-cast thin films exhibit strong PL intensity at ~480 nm (Supplementary Fig. 13) even without thermal annealing. We attribute this result to accelerated ion diffusion under DMF vapour, which facilitates local supersaturation and thus initiates nucleation and crystallization of perovskite. This crystallization pathway is sensitive to the underlying materials. On ITO/PVK (Fig. 3d), perovskite crystallization proceeds smoothly with a higher crystallization rate (Supplementary Fig. 2a). This enables high vertical (Fig. 2b,f) and horizontal (Fig. 1b) homogeneity in perovskite thin films, therefore ultranarrow spectral linewidth (Fig. 1a). In contrast, perovskite crystallization is clearly retarded on ITO/PVP (Supplementary Fig. 2b). Dissolved PVP in perovskite precursors coordinates with lead and thus hinders ion diffusion and perovskite crystallization, resulting in vertical (Fig. 2d,j) and horizontal (Fig. 1e) inhomogeneity, as illustrated in Fig. 3f, eventually resulting in a broader spectral linewidth (Fig. 1a). This mechanistic insight is further supported by the broad FWHM (Fig. 1a) observed for posttreated perovskite on ITO/PVD (17.4 nm) and ITO/NiO_*x*_ (18.5 nm). The carbonyl (C=O) groups in PVD^46^ were reported to coordinate with Pb^2+^, while the hydroxyl (–OH) groups on NiO_*x*_ surfaces^47^ were found to react with perovskite. These interactions disrupt ion diffusion and perovskite crystallization, thereby leading to broad spectral linewidths. Conversely, posttreated perovskite on bare glass exhibits a narrow FWHM (14.2 nm), consistent with minimal interfacial interaction. Collectively, these results underscore the critical role of underlying material–perovskite interfacial chemistry in governing the crystallization and spectral linewidth of perovskite thin films in the presence of DMF vapour.

**Fig. 4:**
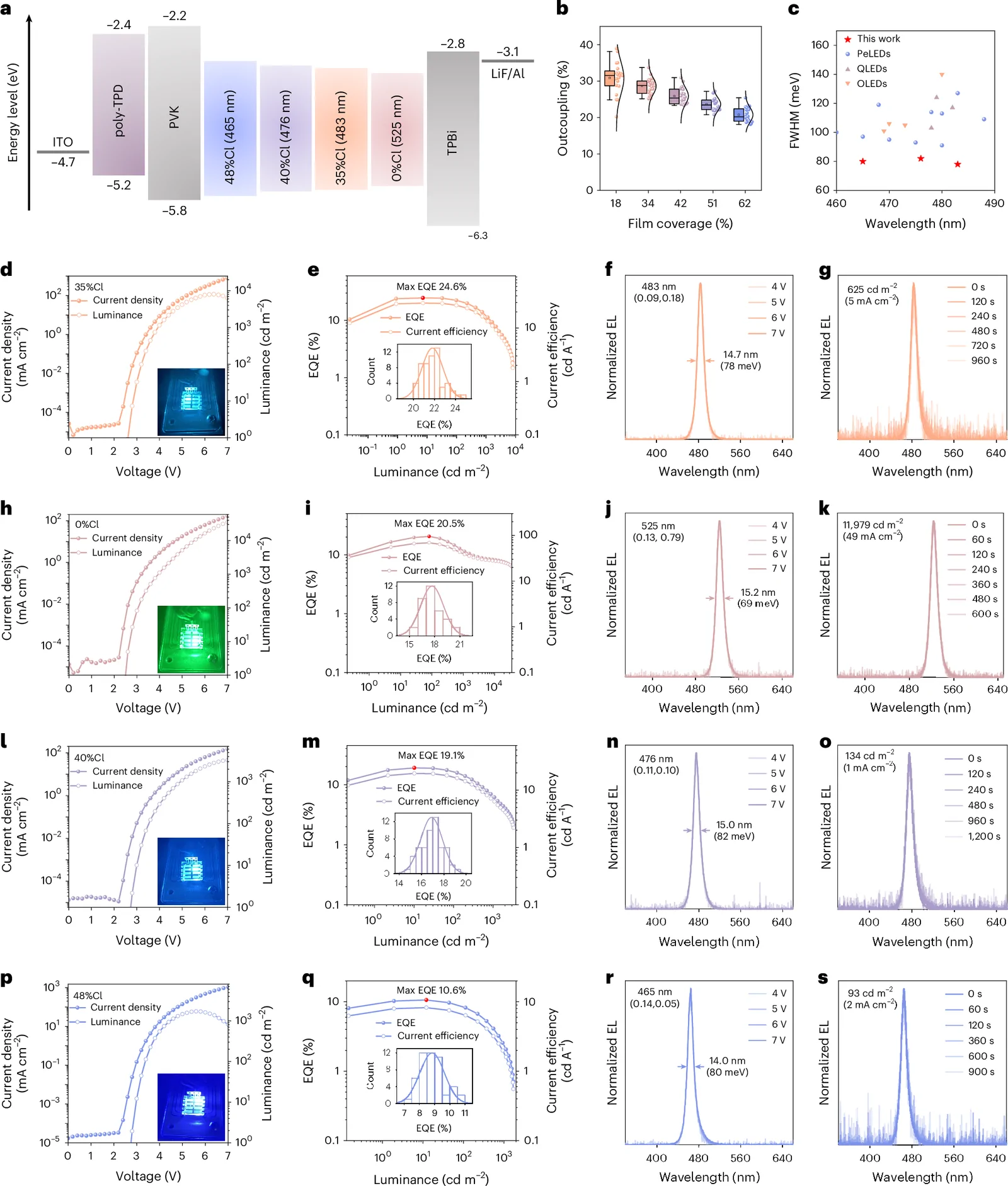
LED devices with ultranarrow linewidth EL enabled by surface-regulated vapour crystallization. **a**, Energy level diagram of PeLEDs with a device structure of ITO/poly-TPD/PVK/TPBi/LiF/Al. **b**, Calculated outcoupling efficiency of PeLEDs based on the film coverage of posttreated 35%Cl perovskite thin films. The film coverage was estimated from their SEM micrographs in Supplementary Fig. 20. For each film coverage, 20 dipoles were simulated and each circular symbol represents the outcoupling efficiency calculated from one dipole. In the box plots, the centre line indicates the median, the box bounds represent the 25th and 75th percentiles, and the whiskers represent the minimum and maximum values. **c**, Reported FWHM of efficient blue LEDs with emission peak between 460 nm and 490 nm according to the data provided in Supplementary Tables 2–4. **d**,**h**,**l**,**p**, Voltage–current density–luminance characteristics of 35%Cl (**d**), 0%Cl (**h**), 40%Cl (**l**) and 48%Cl (**p**) PeLEDs. The inset displays the digital image of their working devices (12 mm × 12 mm). **e**,**i**,**m**,**q**, Luminance–EQE–current efficiency characteristics of 35%Cl (**e**), 0%Cl (**i**), 40%Cl (**m**) and 48%Cl (**q**) PeLEDs. The inset shows the EQE histogram of their devices. **f**,**j**,**n**,**r**, EL spectra of 35%Cl (**f**), 0%Cl (**j**), 40%Cl (**n**) and 48%Cl (**r**) PeLEDs under a bias voltage between 4 V and 7 V. **g**,**k**,**o**,**s**, EL spectra of 35%Cl (**g**), 0%Cl (**k**), 40%Cl (**o**) and 48%Cl (**s**) PeLEDs operated at a constant current density of 5 mA cm^−2^ (initial luminance of 625 cd m^−2^), 49 mA cm^−2^ (initial luminance of 11,979 cd m^−2^), 1 mA cm^−2^ (initial luminance of 134 cd m^−2^) and 2 mA cm^−2^ (initial luminance of 93 cd m^−2^), respectively. OLEDs, organic LEDs; QLEDs, quantum dot LEDs.

Encouraged by the ultranarrow emission in posttreated perovskite on ITO/PVK, we explored their applications in perovskite LEDs (PeLEDs) using a device architecture of ITO (150 nm)/poly(*N*,*N*′-bis-4-butylphenyl-*N*,*N*′-bisphenyl)benzidine (poly-TPD, ~5 nm)/PVK (~15 nm)/perovskite (~45 nm)/2,2′,2′-(1,3,5-benzinetriyl)-tris(1-phenyl-1-*H*-benzimidazole) (TPBi, ~35 nm)/LiF (1.5 nm)/Al (100 nm), as illustrated in the HAADF cross-sectional images (Extended Data Fig. 5a). The PeLEDs energy band diagram is shown in Fig. 4a (ref. ^48^). Device design and optimization (Supplementary Figs. 15–18) are discussed in Supplementary Note 3. In addition, we find that the outcoupling efficiency of the PeLEDs could be improved by reducing the perovskite film coverage (Fig. 4b). Detailed analysis of the outcoupling simulation (Supplementary Figs. 19–22) is provided in Supplementary Note 4. Therefore, we demonstrate efficient sky-blue (483 nm) PeLEDs with a peak external quantum efficiency (EQE) of 24.6% at a luminance of 7.3 cd m^−2^ (Fig. 4e), a peak luminance of 7,997 cd m^−2^ (Fig. 4d) and an ultranarrow FWHM of 14.7 nm (78 meV) maintained across 4–7 V (Fig. 4f). The corresponding Commission Internationale de l’Éclairage colour coordinate is (0.09, 0.18). The digital image and video clip of an operating device are provided in Fig. 4d (inset) and Supplementary Video 1. This represents a very narrow FWHM compared with reported efficient blue LEDs based on III–V semiconductors^22,49^, chalcogenide quantum dots^8,9,24,25,50–52^, organics^10,12,13,26,53–57^ or halide perovskite^1,27,58–62^ (Fig. 4c). The peak EQE is comparable to the state-of-the-art sky-blue PeLEDs (Supplementary Table 2) and the peak luminance outperforms most highly efficient sky-blue PeLEDs. Moreover, the devices exhibit good electroluminescence (EL) spectral stability under a constant current density of 0.5 mA cm^−2^ (Supplementary Fig. 23a) and 5 mA cm^−2^ (Fig. 4g) for 1,200 seconds. Even at a high current density injection of 50 mA cm^−2^, the devices exhibit only slight broadening over 600 seconds (Supplementary Fig. 23c). However, the operational EL half-lifetime (420 seconds) of the device remains comparable to the reported high-performance blue PeLEDs^27,59,62,63^, highlighting a central challenge in this field (Supplementary Note 5). We further evaluated the device performance under pulsed operation to reduce Joule heating^64^. Under a pulsed bias of 18.7 V, the devices achieve a peak luminance of ~91,539 cd m^−2^ (Supplementary Fig. 24a), which is more than an order of magnitude higher than that under continuous operation at 6.4 V (7,997 cd m^−2^).

To evaluate the generality of our approach, we extended the strategy to PeLEDs incorporating 0%Cl, 40%Cl and 48%Cl perovskite as the emissive layers using the same device architecture. The 0%Cl perovskite thin films achieved a minimal FWHM of 14.4 nm (66 meV) on ITO/PVK after vapour exposure (Supplementary Fig. 25), while exhibiting a similar isolated morphology (Supplementary Fig. 26). The digital images of 0%Cl, 40%Cl and 48%Cl PeLEDs in operation are provided in the insets of Fig. 4h,l,p, respectively, with corresponding video clips provided in Supplementary Videos 2–4. The 0%Cl, 40%Cl and 48%Cl PeLEDs exhibit EL peaks at 525 nm (Fig. 4j), 476 nm (Fig. 4n) and 465 nm (Fig. 4r) with narrow FWHMs of 15.2 nm (69 meV), 15.0 nm (82 meV) and 14.0 nm (80 meV), rendering Commission Internationale de l’Éclairage colour coordinates of (0.13, 0.79), (0.11, 0.10) and (0.14, 0.05), respectively. The peak EQEs of 0%Cl, 40%Cl and 48%Cl PeLEDs are 20.5% (Fig. 4i), 19.1% (Fig. 4m) and 10.6% (Fig. 4q), with peak luminance reaching 35,400 cd m^−2^ (Fig. 4h), 3,305 cd m^−2^ (Fig. 4l) and 1,713 cd m^−2^ (Fig. 4p), respectively. We note that all devices exhibit similar luminance roll-off at current densities of several hundred mA cm^−2^, which is consistent with previous reports^1,65^ and indicative of a common challenge in the field. We assert that Joule heating should be a major contributing factor, particularly given the poor thermal conductivity of glass substrates^66^. Despite that, all devices maintain good EL spectral stability under constant current densities of 49 mA cm^−2^ (Fig. 4k and Supplementary Fig. 27a), 1 mA cm^−2^ (Fig. 4o and Supplementary Fig. 28a) and 2 mA cm^−2^ (Fig. 4s and Supplementary Fig. 29a) over 600 seconds, 1,200 seconds and 900 seconds, respectively. The corresponding unnormalized EL spectra are provided in Supplementary Fig. 30.

## Discussion

Our results demonstrate that interface-regulated vapour crystallization is a simple but powerful strategy that enables precise control over the spectral linewidth of solution-processed perovskite thin films by regulating their crystallization. By using underlying materials with minimal molecular interactions with perovskite precursors, exemplified by PVK, we achieve mixed-cation, mixed-halide perovskite thin films with high horizontal and vertical homogeneity. This uniformity enables ultranarrow spectral linewidth in thin film emitters spanning the pure blue to pure green range. Our strategy and findings highlight a key advantage of halide perovskite over traditional semiconductors (III–V semiconductors, chalcogenide quantum dots and organics) in producing ultrapure emission across a wide spectral range without the need for complex epitaxial techniques or sophisticated fabrication conditions, opening new opportunities for next-generation optoelectronic technologies that require wavelength specific ultrapure emitters.

## Methods

### Materials

Rubidium bromide (RbBr, 99.6%), caesium bromide (CsBr, 99.999%), lead bromide (PbBr_2_, 99.999%), lead chloride (PbCl_2_, 99.999%), poly-TPD (molecular weight ≥20,000 g mol^−1^), PVP (molecular weight ~60,000), PVD (molecular weight 40,000), PVK (Mn 25,000–50,000), TTDDA (97%), lithium fluoride (LiF, 99.99%), DMSO (99.9%), *N*,*N*-DMF (DMF, 99.8%), chlorobenzene (CB) (99.8%) and 2-propanol (isopropyl alcohol (IPA), 99.5%) were purchased from Sigma Aldrich. Formamidinium bromide (FABr) was obtained from Greatcell Solar Materials. TPBi was purchased from Ossila and NiO_*x*_ nanocrystals were purchased from Avantama AG.

### Fabrication of perovskite thin films

Perovskite thin films with 35%Cl, 40%Cl and 48%Cl content were prepared from precursors with the following molar ratios, RbBr:CsBr:FABr:PbBr_2_:PbCl_2_:TTDDA = 0.2:1.1:0.2:*x*:1 − *x*:0.1 where *x* equals 0.3875, 0.30 and 0.16, respectively. The 0%Cl perovskite thin films were prepared using CsBr:FABr:PbBr_2_:TTDDA = 1.1:0.4:1:0.1. All precursors were dissolved in DMSO with Pb concentrations ranging from 0.06 M to 0.14 M to tune film coverage. ITO substrates were coated with 25 μl of PVP (6 mg ml^−1^ in IPA), PVD (6 mg ml^−1^ in IPA) or PVK (6 mg ml^−1^ in CB) by spin-coating in a glovebox, followed by thermal annealing at 100 °C for 10 min. Next, 25 μl of NiO_*x*_ layers were spin-coated from an ethanol-diluted dispersion (v:v = 3:7) in a fume hood and annealed at 150 °C for 10 min. After cooling to room temperature, 20-μl perovskite thin films were spin-coated onto the various underlying materials (ITO/PVP, ITO/PVD, ITO/PVK, ITO/NiO_*x*_, bare ITO or bare glass) and exposed to 20 μl of DMF in a Petri dish for a defined duration, followed by annealing at 80 °C for 8 min.

### Fabrication and characterization of PeLEDs

Here 20 μl of poly-TPD (4 mg ml^−1^ in CB) was spin-coated on ITO and annealed at 120 °C for 10 min. Subsequently, 20 μl of PVK (8 mg ml^−1^ in CB) was deposited and annealed at 100 °C for 10 min. A thin PVP layer (0 mg ml^−1^, 1.5 mg ml^−1^, 3 mg ml^−1^ or 6 mg ml^−1^ in IPA) was spin-coated and annealed at 80 °C for 10 min. Perovskite layers were deposited on top, exposed to 20 μl of DMF vapour and annealed at 80 °C for 8 min. TPBi (35 nm), LiF (1.5 nm) and Al (100 nm) were thermally evaporated through shadow masks. The active device area was 4.5 mm^2^.

All PeLEDs were encapsulated with glass covers using BluFixx epoxy. Current–voltage–luminance characteristics were measured in air using a Keithley 2400 source meter and a calibrated silicon photodiode. EQE was calculated considering the detector’s spectral responsivity. EL spectra were recorded using an Ocean Optics Flame spectrometer.

For pulsed operation, square voltage pulses (500 ns width, 10 Hz repetition) were generated by an HP8116A function generator (rise time <7 ns) and amplified using a custom-built circuit. Transient electrical responses were recorded with a digital oscilloscope (RSPRO RSDS 1304CFL). Time-resolved EL spectra were collected using an intensified CCD (Andor iStar DH740) coupled with a grating spectrometer (Andor SR303i).

### PL and PL stability

Steady-state PL spectra were obtained using an Edinburgh FLS1000 equipped with a xenon arc lamp (excitation at 400 nm). PL stability was evaluated using continuous 405-nm laser excitation (Thorlabs) with spectral acquisition every minute using a Princeton Instrument HRS-500 spectrometer.

### Transient PL

Fluence-dependent transient PL was measured using an Edinburgh FLS1000 system with a pulsed laser at 405 nm (HPL-405) and a repetition rate of 2 MHz. Fluence of 11.20 nJ pulse^−1^ cm^−2^, 2.41 nJ pulse^−1^ cm^−2^ and 0.20 nJ pulse^−1^ cm^−2^ were used to excite the samples and achieved with a motor-controlled filter wheel.

### PLQY

PL quantum yield (PLQY) was measured in an integrating sphere using the three-measurement approach from ref. ^67^. A 405-nm continuous-wave laser (CNI MGL-III-405) was used for excitation and emission was collected via a Teledyne PIXIS:1024BR_eXcelon detector and HRS-500 monochromator. This system was calibrated with a halogen source (AvaLight-HALCAL-ISP50-MINI, AVANTES).

### Hyperspectral PL microscopy

Cryogenic hyperspectral PL microscopy was performed using a Photon etc. IMA system. A Zeiss Plan-Neofluar objective lens with a numerical aperture of 0.75 and a magnification of ×63 was used for the measurements. To prevent degradation due to oxygen and humidity, samples were kept in a nitrogen-filled glovebox until just before measurement. For temperature-dependent experiments, samples were affixed with silver paste to the cold finger of an Oxford HiRes cryostat, which was cooled with liquid helium. Each sample was maintained at the target temperature for a minimum of 15 min before measurement. Cooling and heating were carried out at a maximum rate of 1 °C min^−1^ to prevent any unintended thermal stress effects. To correct aberrations caused by optical elements along the detection path, the system was calibrated using a reference sample, which provided postprocessing parameters to rectify image distortion. In addition, chromatic aberrations were minimized by calibrating the sample’s *z*-position for each collected wavelength. A 400-nm picosecond pulsed laser was used as the excitation source, operating at a repetition rate of 100 kHz with a fluence of 0.68 µJ cm^−2^ and a top hat profile spanning 150 µm × 150 µm. These repetition rates were chosen as the highest values at which no laser-induced decrease in PL intensity was observed in vacuum, an indicator commonly associated with laser-induced damage. PL was collected from an 85 µm × 85 µm field of view using a microscope. While hyperspectral microscopy allows for PL spectra collection at every pixel, the PL response was integrated across the entire field of view to enhance the signal-to-noise particularly important at elevated temperatures where PL is weaker. To separate the PL signal from the excitation light, a high-quality 405-nm Semrock dichroic mirror was used to filter the excitation laser. Emitted light from the sample was directed onto a volume Bragg grating, which spectrally dispersed the light onto a Hamamatsu silicon CMOS camera with a 2,048 × 2,048 pixel array, where each pixel measured 6.5 µm × 6.5 µm. The PL FWHM was extracted by fitting the PL spectrum to a Voigt function.

The temperature-dependence of the FWHM was modelled using equation (1)^28,29^

1 $$\Gamma \,=\,{\Gamma }_{0}+{\gamma }_{{\rm{ac}}}T+{\gamma }_{{\rm{LO}}}/[{{\rm{e}}}^{{E}_{{\rm{LO}}}/{k}_{{\rm{B}}}T}-1]$$

where *Γ*_0_ is a temperature-independent inhomogeneous broadening term, *γ*_ac_ is carrier-acoustic phonon coupling strength, *γ*_LO_ is carrier-longitudinal optical phonon coupling strength, *E*_LO_ is the longitudinal optical phonon energy, *k*_B_ is the Boltzmann constant and *T* is the temperature. Note that for the perovskite on ITO/PVP, the fitting was restricted to the 4–180 K range where the FWHM primarily originates from the dominant peak at longer wavelengths.

Room temperature hyperspectral PL microscopy was conducted using above same system. A ×100 chromatic aberration-corrected air objective lens from Olympus was used. The perovskite thin films were excited with a 405-nm continuous-wave laser. The CoM PL peak was calculated using the following equation ${\rm{CoM}}=\frac{\sum \lambda {I}_{{\rm{PL}}}(\lambda )}{\sum {I}_{{\rm{PL}}}(\lambda )}$.

### Transient absorption spectroscopy

Picosecond transient absorption spectroscopy measurements were performed using a commercial transient absorption system (HARPIA, Light Conversion). A broadband UV-visible (UV-Vis) probe spanning 350–530 nm was generated by pumping a sapphire crystal with a focused 515-nm beam, followed by re-collimation. The 515-nm beam was generated by the second-harmonic generation of the 1,030-nm Yb:KGW amplifier laser (Light Conversion Pharos, 20 W, 10 kHz, 160 fs). The pump beam at 400 nm was generated using a commercial optical parametric amplifier (Light Conversion, ORPHEUS-Neo). The pump beam was weakly focused onto the sample, creating a near-Gaussian (FWHM ~1,100 μm) excitation profile with fluences ranging from 4 μJ cm^−2^ to 70 μJ cm^−2^. The probe beam was tightly focused (FWHM ~250 μm) in a non-collinear geometry and mechanically delayed relative to the pump. After transmission through the sample at various pump-probe delay times, the probe was spectrally dispersed and detected. The Δ*T/T*(*λ*,*t*) are calculated by separately recording *T*_pump,on_(*λ*,*t*) and *T*_pump,off_(*λ*,*t*) spectra through pump modulation at 100 Hz using a mechanical chopper.

### UV-Vis absorption spectroscopy

Transmittance and reflectance spectra of perovskite thin films were collected using an Agilent Cary 7000 UV-Vis-near-infrared Universal Measurement Spectrometer equipped with a diffuse reflectance accessory. The 100% reflectance and transmittance baseline were established using a reference polytetrafluoroethylene plate placed at the reflectance port. Measurements were performed over 300–550 nm with an averaging time of 0.4 s and a slit bandwidth of 2 nm. Absorptance was calculated as: absorptance = 100% − transmittance − reflectance.

### SEM

SEM images were acquired using a Zeiss GeminiSEM 300 operated at 5 kV with a 30-μm aperture and in-lens detector. Film coverage was quantified using ImageJ.

### AFM

AFM was performed using an Asylum Research MFP-3D system (alternating current mode) with a silicon cantilever (Bruker, spring constant 48 N m^−1^, resonant frequency ~170 kHz). C-AFM was conducted using an Asylum Research Cypher ES AFM system under nitrogen flow, using an iridium-coated conductive tip (ASYELEC.01-R2) in contact mode. Samples were mounted on a stainless-steel puck and electrically grounded with a silver wire and conductive silver paste. The tip had a bias voltage applied of 3 V. Since any off-set voltage between the tip and the sample had not been calibrated, a standard off-set on the order of 30 mV was always present. This off-set caused non-zero currents in highly conducting samples even without bias. Current amplification was performed by the Dual Gain ORCA cantilever holder, which allowed measurement of a wide range of currents (~10 pA–10 μA).

### SED

SiN_*x*_ TEM grids (Norcada NT025X) were first treated with oxygen plasma and then spin-coated with 6 mg ml^−1^ PVK or PVP. 35%Cl perovskite was spin-coated on SiN_*x*_/PVK or SiN_*x*_/PVP and then treated with DMF vapour treatment for 12 min. SED was performed on the perovskite using a ThermoFisher Spectra 300 STEM operated at 200 kV accelerating voltage, 423 μrad convergence semi-angle and a camera length of 145 mm. Diffraction patterns were recorded in 256 × 256 maps on a Quantum Detectors Merlin/Medipix3 single chip direct electron detector with a dwell time of 1 ms, and data were recorded with an approximate current of either ~2 pA or ~60 pA depending on the grain size. If a stationary probe is considered we maintain a maximum fluence (*D*_p_) of ~12 e^−^Å^−2^ for both the PVK and PVP samples when changing current, the rationale for this is as follows: when measuring at 2 pA our probe is diffraction limited to have a diameter of ~3.62 nm (FWHM) (d* = ~5.25 nm)^68^, when the current is increased to 60 pA the probe in-turn increases in size to a diameter of ~19.8 nm (FWHM) (d* = ~28.71 nm)^68^ to maintain brightness down the column. When scanning as our estimated probe size is on the order of the pixel size, or indeed larger, we quote frame limited fluences *D*_f_ in the figure captions^68^. Annular dark-field images were recorded concurrently with SED data with an inner and outer detector collection semi-angle of 62 mrad and 200 mrad, respectively. Reciprocal space calibrations were performed via the use of a gold cross grating calibration sample, recorded at the same camera length. Postacquisition analysis of SED data was performed using pyXem v.0.16^69^. In accordance with the standard practices in the electron diffraction community, we quote reciprocal lattice vectors as **g**, not **q** as has been done for GIWAXS measurements, there is therefore a factor of 2*π* between the electron and X-ray diffraction experiments. EDX analysis was recorded similarly to the SED data using a super-X system. To improve the signal-to-noise for EDX quantification multiple EDX scans were drift corrected and combined. The diffraction patterns were indexed to a corner-sharing pseudo-cubic phase (*Pm*3^−^*m, a* = 5.76 Å).

### STEM

The cross-sectional lamellae for STEM-EDX were prepared with an FEI HeliosNanolab Dualbeam focused ion beam/SEM according to a standard procedure. The lamellae were immediately transferred into an FEI Osiris (S)TEM operated at 200 kV with air exposure minimized to 2 min. The HAADF micrograph was acquired with a Fischione detector with a dwell time of 1 μs per pixel and a beam current of 64 pA. STEM-EDX spectra were obtained with a Bruker Super-X silicon drift detector with a solid collection angle of about 0.9 sr. To minimize beam damage, a defocused beam (Δ*f*¼ = 1 μm) with a spatial sampling of 7 nm per pixel was used. The STEM-EDX data were denoised with principal component analysis and non-matrix factorization, and processed in HyperSpy, a Python-based analysis suite for hyperspectral data. In EDX analysis, elemental concentrations in materials were quantified by Cliff–Lorimer factors (*k*-factors).

### GIWAXS

GIWAXS measurements were conducted at the I07 beamline at the Diamond Light Source (UK). X-ray beam energy of 10 keV with an incident angle of 0.04–0.4° was used. The sample detector (the Pilatus 2M detector) distance was approximately 0.41 m for measurements. Calibration of reciprocal space was performed using a lanthanum hexaboride (LaB_6_) reference sample. All presented GIWAXS maps were obtained with a dwell time of 1 s under inert conditions (He gas flow). To index GIWAXS peaks, we used an orthorhombic unit cell with a *Pnma* space group (*a* = 8.131 Å, *b* = 11.574 Å, *c* = 8.099 Å) and note that we quoted reciprocal lattice vectors in **q**, not **g**, as was done for the SED data.

### FTIR

FTIR spectra were measured using a Bruker Vertex 70 v spectrometer. For transmission spectra, double-sided polished Si substrates (PI-KEM, 1–100 Ω·cm, p-type, thickness of 600–650 μm) were used as substrates. PVP and PVK thin films were deposited on Si using their solution in IPA and CB, respectively. PbBr_2_ + PVP and PbBr_2_ + PVK thin films were prepared using their solution in DMSO. Note that heating at 65 °C was required to obtain a clear PbBr_2_ + PVK solution.

### XPS

XPS was measured using a ThermoFisher Nexsa system under an ultrahigh vacuum of below 1.8 × 10^−9 ^mbar. An electron flood gun was used to neutralize charge build-up. The XPS spectra were calibrated using their C1s peak (binding energy of 284.8 eV). PbBr_2_, PbBr_2_ + PVK and PbBr_2_ + PVP thin films were prepared using their solution in DMSO.

XPS of 35%Cl perovskite thin films on ITO after vapour exposure was conducted in a SPECS ultrahigh vacuum system using an XR50 X-ray source (Al K-Alpha radiation at 1,486.74 eV), FOCUS 500 X-ray monochromator and a PHOIBOS 100 hemispherical analyser. Samples were electrically contacted to the sample holder using double-sided copper tape and silver paste. For chemical composition analysis, narrow scans were deconvoluted with the CasaXPS software. The spectra were fitted using a Shirley approach and a mixed Gaussian–Lorentzian (50–50) line shape. Scans were charge calibrated to C 1*s* (adventitious) to 284.8 eV. Quantification was performed by using elemental sensitivity factors provided by the CasaXPS Kratos library. To ensure accuracy in area determination and peak fitting, doublet peak fitting was conducted using the area, FWHMs and spin–orbit splitting position constraints based on the NIST XPS database (https://srdata.nist.gov/xps).

### TOF-SIMS

TOF-SIMS was performed using an ION TOF.SIMS 5 system in both positive and negative ion modes. For positive mode, the depth profiles of FA^+^, Cs^+^, Rb^+^, Pb^+^ and C_7_H_8_N^+^ were obtained using an O_2_^+^ sputter ion beam (0.25 keV, 30 nA) over an area of 300 × 300 μm^2^ (sputtering ion dose density of 4.26 × 10^17 ^ions cm^−2^) and a Bi_3_^+^ primary ion beam (30 keV, 0.5 pA) for analysis over an area of 100 × 100 μm^2^ (primary ion dose density of 6.39 × 10^13 ^ions cm^−2^). For negative mode, the depth profiles of Cl^−^, Br^−^ and C_12_H_8_N^−^ were acquired using a Cs^+^ sputter ion beam (0.25 keV, 10 nA) over the same area of 300 × 300 μm^2^ (sputtering ion dose density of 1.87 × 10^17 ^ions cm^−2^), with analysis performed using the Bi_3_^+^ primary ion beam (30 keV, 0.5 pA) over an area of 100 × 100 μm^2^ (primary ion dose density of 8.43 × 10^13 ^ions cm^−2^).

### Light outcoupling simulations

We performed three-dimensional finite-difference time-domain simulations on PeLED models constructed with disordered perovskite patterns (lumerical finite-difference time domain). The device model is shown in Supplementary Fig. 19. The glass substrates, charge-transport layers and electrodes were modelled as planar multilayers. The disordered patterns of perovskite grains were imported from SEM micrographs that were converted into binary images. The perovskites were modelled as a non-absorbing medium and the entire organic stack was modelled as a single effective medium with average optical constants. While this approximation may limit spatial resolution, it still captures the essential interference and outcoupling effects within the multilayer devices.

The simulation region was 15 μm × 15 μm in the horizontal direction and 0.8 μm in the vertical direction. The boundary conditions were set as metal type for the top electrode and perfectly matched layer (PML) type for the other interfaces. The power of the out-coupled light was obtained from the far-field projection of the transmitted light in the glass substrate. To simulate light outcoupling from a disordered pattern, monochromatic dipoles (485 nm, 3.2-fs pulse) were injected into 20 different horizontal locations in the perovskite grains with a fixed vertical position within the grains (25 nm to the electron-transport layer (ETL) interface) independently. The dipole positions were near the central region of the model (1.5 μm × 1.5 μm) to ensure a complete outcoupling in the normal direction before the light reaches the horizontal boundaries. For each dipole position, three orthogonal dipole orientations were respectively simulated and then averaged. The light outcoupling efficiency of the PeLED was obtained from the average value of the results from 20 dipole positions.

## Supplementary information

Supplementary Information Supplementary Figs. 1–41, Notes 1–5 and Tables 1–4.

Supplementary Video 1 35%Cl PeLEDs.

Supplementary Video 2 0%Cl PeLEDs.

Supplementary Video 3 40%Cl PeLEDs.

Supplementary Video 4 48%Cl PeLEDs.

## Data Availability

The data underlying this paper are available via the University of Cambridge at https://doi.org/10.17863/CAM.129885.

## References

[1] Jiang, Y. Z. et al. Synthesis-on-substrate of quantum dot solids. *Nature* **612**, 679–684 (2022). 10.1038/s41586-022-05486-3 36543955

[2] García de Arquer, F. P. et al. Semiconductor quantum dots: technological progress and future challenges. *Science* **373**, eaaz8541 (2021). 10.1126/science.aaz8541 34353926

[3] Hong, G., Antaris, A. L. & Dai, H. Near-infrared fluorophores for biomedical imaging. *Nat. Biomed. Eng.* **1**, 0010 (2017).

[4] Yang, Y. Y. et al. Wireless multilateral devices for optogenetic studies of individual and social behaviors. *Nat. Neurosci.* **24**, 1035–1045 (2021). 10.1038/s41593-021-00849-x PMC8694284 33972800

[5] Shields, A. J. Semiconductor quantum light sources. *Nat. Photon.* **1**, 215–223 (2007).

[6] Iida, D. & Ohkawa, K. Recent progress in red light-emitting diodes by III-nitride materials. *Semicond. Sci. Technol.* **37**, 013001 (2021).

[7] Wu, Q. Q. et al. Homogeneous ZnSeTeS quantum dots for efficient and stable pure-blue LEDs. *Nature* **639**, 633–638 (2025). 10.1038/s41586-025-08645-4 40044864

[8] Deng, Y. Z. et al. Solution-processed green and blue quantum-dot light-emitting diodes with eliminated charge leakage. *Nat. Photon.* **16**, 505–511 (2022).

[9] Kim, T. et al. Efficient and stable blue quantum dot light-emitting diode. *Nature* **586**, 385–389 (2020). 10.1038/s41586-020-2791-x 33057219

[10] Zhao, H., Arneson, C. E., Fan, D. & Forrest, S. R. Stable blue phosphorescent organic LEDs that use polariton-enhanced Purcell effects. *Nature* **626**, 300–305 (2023). 10.1038/s41586-023-06976-8 38122821

[11] Aizawa, N. et al. Delayed fluorescence from inverted singlet and triplet excited states. *Nature* **609**, 502–506 (2022). 10.1038/s41586-022-05132-y PMC9477729 36104553

[12] Chan, C.-Y. et al. Stable pure-blue hyperfluorescence organic light-emitting diodes with high-efficiency and narrow emission. *Nat. Photon.* **15**, 203–207 (2021).

[13] Kondo, Y. et al. Narrowband deep-blue organic light-emitting diode featuring an organoboron-based emitter. *Nat. Photon.* **13**, 678–682 (2019).

[14] Fakharuddin, A. et al. Perovskite light-emitting diodes. *Nat. Electron.* **5**, 203–216 (2022).

[15] Liu, X.-K. et al. Metal halide perovskites for light-emitting diodes. *Nat. Mater.* **20**, 10–21 (2020). 10.1038/s41563-020-0784-7 32929252

[16] Han, T.-H. et al. A roadmap for the commercialization of perovskite light emitters. *Nat. Rev. Mater.* **7**, 757–777 (2022).

[17] Shinde, A., Gahlaut, R. & Mahamuni, S. Low-temperature photoluminescence studies of CsPbBr_3_ quantum dots. *J. Phys. Chem. C* **121**, 14872–14878 (2017).

[18] Chen, Z. et al. Photoluminescence enhancement for efficient mixed-halide blue perovskite light-emitting diodes. *Adv. Opt. Mater.* **11**, 2202525 (2023).

[19] Luo, X. Y. et al. Effects of local compositional heterogeneity in mixed halide perovskites on blue electroluminescence. *Matter* **7**, 1054–1070 (2024).

[20] Karlsson, M. et al. Mixed halide perovskites for spectrally stable and high-efficiency blue light-emitting diodes. *Nat. Commun.* **12**, 361 (2021). 10.1038/s41467-020-20582-6 PMC7806600 33441549

[21] Chen, Y. et al. All-site alloyed perovskite for efficient and bright blue light-emitting diodes. *Nat. Commun.* **16**, 3254 (2025). 10.1038/s41467-025-58470-6 PMC11972346 40188089

[22] Sheen, M. et al. Highly efficient blue InGaN nanoscale light-emitting diodes. *Nature* **608**, 56–61 (2022). 10.1038/s41586-022-04933-5 35922503

[23] Shin, J. et al. Vertical full-colour micro-LEDs via 2D materials-based layer transfer. *Nature* **614**, 81–87 (2023). 10.1038/s41586-022-05612-1 36725999

[24] Shen, H. B. et al. Visible quantum dot light-emitting diodes with simultaneous high brightness and efficiency. *Nat. Photon.* **13**, 192–197 (2019).

[25] Yang, Y. et al. High-efficiency light-emitting devices based on quantum dots with tailored nanostructures. *Nat. Photon.* **9**, 259–266 (2015).

[26] Stavrou, K., Franca, L. G., Danos, A. & Monkman, A. P. Key requirements for ultraefficient sensitization in hyperfluorescence organic light-emitting diodes. *Nat. Photon.* **18**, 554–561 (2024).

[27] Gao, Y. et al. Highly efficient blue light-emitting diodes based on mixed-halide perovskites with reduced chlorine defects. *Sci. Adv.* **10**, eado5645 (2024). 10.1126/sciadv.ado5645 PMC466955 39018409

[28] Wright, A. D. et al. Electron-phonon coupling in hybrid lead halide perovskites. *Nat. Commun.* **7**, 11755 (2016). 10.1038/ncomms11755 PMC4894981 27225329

[29] Sebastian, M. et al. Excitonic emissions and above-band-gap luminescence in the single-crystal perovskite semiconductors CsPbBr_3_ and CsPbCl_3_. *Phys. Rev. B* **92**, 235210 (2015).

[30] Milot, R. L., Eperon, G. E., Snaith, H. J., Johnston, M. B. & Herz, L. M. Temperature-dependent charge-carrier dynamics in CH_3_NH_3_PbI_3_ perovskite thin films. *Adv. Funct. Mater.* **25**, 6218–6227 (2015).

[31] Woo, H. C. et al. Temperature-dependent photoluminescence of CH_3_NH_3_PbBr_3_ perovskite quantum dots and bulk counterparts. *J. Phys. Chem. Lett.* **9**, 4066–4074 (2018). 10.1021/acs.jpclett.8b01593 29975057

[32] Liu, S. et al. Cryogenic photoluminescence study on perovskite MAPbBr_3_ single crystals. *ECS J. Solid State Sci. Technol.* **10**, 046003 (2021).

[33] Gramlich, M., Lampe, C., Drewniok, J. & Urban, A. S. How exciton-phonon coupling impacts photoluminescence in halide perovskite nanoplatelets. *J. Phys. Chem. Lett.* **12**, 11371–11377 (2021). 10.1021/acs.jpclett.1c03437 34791883

[34] Doherty, T. A. S. et al. Performance-limiting nanoscale trap clusters at grain junctions in halide perovskites. *Nature* **580**, 360–366 (2020). 10.1038/s41586-020-2184-1 32296189

[35] Stoumpos, C. C. et al. Crystal growth of the perovskite semiconductor CsPbBr_3_: a new material for high-energy radiation detection. *Cryst. Growth. Des.* **13**, 2722–2727 (2013).

[36] Linaburg, M. R., McClure, E. T., Majher, J. D. & Woodward, P. M. Cs_1–*x*_Rb_*x*_PbCl_3_ and Cs_1–*x*_Rb_*x*_PbBr_3_ solid solutions: understanding octahedral tilting in lead halide perovskites. *Chem. Mater.* **29**, 3507–3514 (2017).

[37] Saliba, M. et al. Incorporation of rubidium cations into perovskite solar cells improves photovoltaic performance. *Science* **354**, 206–209 (2016). 10.1126/science.aah5557 27708053

[38] Liang, Z. et al. Homogenizing out-of-plane cation composition in perovskite solar cells. *Nature* **624**, 557–563 (2023). 10.1038/s41586-023-06784-0 PMC10733143 37913815

[39] Botta, A. et al. Synthesis, characterization, and use as emissive layer of white organic light emitting diodes of the highly isotactic poly(*N*-pentenyl-carbazole). *Polym. Compos.* **36**, 1110–1117 (2015).

[40] Santana, A. L., Noda, L. K., Pires, A. T. N. & Bertolino, J. R. Poly (4-vinylpyridine)/cupric salt complexes: spectroscopic and thermal properties. *Polym. Test.* **23**, 839–845 (2004).

[41] Gill, N. S., Nuttall, R. H., Scaife, D. E. & Sharp, D. W. A. The infra-red spectra of pyridine complexes and pyridinium salts. *J. Inorg. Nucl. Chem.* **18**, 79–87 (1961).

[42] Ma, D. X. et al. Distribution control enables efficient reduced-dimensional perovskite LEDs. *Nature* **599**, 594–598 (2021). 10.1038/s41586-021-03997-z 34819678

[43] Wong, C. P. J., Ng, K. M., Weng, L. T., Lun Yeung, K. & Chan, C. M. Analysis of ToF-SIMS spectra of poly(2-vinylpyridine) and poly(4-vinylpyridine) with density functional theory calculations. *Surf. Interface Anal.* **49**, 1095–1107 (2017).

[44] Weng, L. T. et al. Sulfonation of poly(-vinylcarbazole) studied by combined time of-flight secondary ion mass spectrometry and X-ray photoelectron spectroscopy. *Anal. Chem.* **72**, 4908–4913 (2000). 10.1021/ac000152c 11055708

[45] Chen, S. S. et al. Crystallization in one-step solution deposition of perovskite films: upward or downward?. *Sci. Adv.* **7**, eabb2412 (2021). 10.1126/sciadv.abb2412 PMC10670903 33523938

[46] Yu, X. et al. Polyvinyl pyrrolidone induced ‘confinement effect’ on PbI_2_ and the improvement on crystallization and thermal stability of perovskite. *Small* **20**, 2306101 (2023). 10.1002/smll.202306101 37759427

[47] Wu, T. H. et al. Elimination of light-induced degradation at the nickel oxide-perovskite heterojunction by aprotic sulfonium layers towards long-term operationally stable inverted perovskite solar cells. *Energy Environ. Sci.* **15**, 4612–4624 (2022).

[48] Mao, J. et al. All-perovskite emission architecture for white light-emitting diodes. *ACS Nano* **12**, 10486–10492 (2018). 10.1021/acsnano.8b06196 30222315

[49] Baek, W. J. et al. Ultra-low-current driven InGaN blue micro light-emitting diodes for electrically efficient and self-heating relaxed microdisplay. *Nat. Commun.* **14**, 1386 (2023). 10.1038/s41467-023-36773-w PMC10023660 36932091

[50] Zhang, W. J. et al. Stable and efficient pure blue quantum-dot LEDs enabled by inserting an anti-oxidation layer. *Nat. Commun.* **15**, 783 (2024). 10.1038/s41467-024-44894-z PMC10817946 38278797

[51] Chen, X. T. et al. Blue light-emitting diodes based on colloidal quantum dots with reduced surface-bulk coupling. *Nat. Commun.* **14**, 284 (2023). 10.1038/s41467-023-35954-x PMC9845229 36650161

[52] Pu, C. D. et al. Electrochemically-stable ligands bridge the photoluminescence-electroluminescence gap of quantum dots. *Nat. Commun.* **11**, 937 (2020). 10.1038/s41467-020-14756-5 PMC7028909 32071297

[53] Xing, L. J. et al. Highly efficient pure-blue organic light-emitting diodes based on rationally designed heterocyclic phenophosphazinine-containing emitters. *Nat. Commun.* **15**, 6175 (2024). 10.1038/s41467-024-50370-5 PMC11263564 39039042

[54] Hua, T. et al. Deep-blue organic light-emitting diodes for ultrahigh-definition displays. *Nat. Photon.* **18**, 1161–1169 (2024).

[55] Cho, H. H. et al. Suppression of Dexter transfer by covalent encapsulation for efficient matrix-free narrowband deep blue hyperfluorescent OLEDs. *Nat. Mater.* **23**, 519–526 (2024). 10.1038/s41563-024-01812-4 PMC10990937 38480865

[56] Sun, J. et al. Exceptionally stable blue phosphorescent organic light-emitting diodes. *Nat. Photon.* **16**, 212–218 (2022).

[57] Jeon, S. O. et al. High-efficiency, long-lifetime deep-blue organic light-emitting diodes. *Nat. Photon.* **15**, 208–215 (2021).

[58] Yuan, S. et al. Efficient blue electroluminescence from reduced-dimensional perovskites. *Nat. Photon.* **18**, 425–431 (2024).

[59] Zhang, K. et al. Nondestructive halide exchange via S_N_2-like mechanism for efficient blue perovskite light-emitting diodes. *Nat. Commun.* **15**, 10621 (2024). 10.1038/s41467-024-55074-4 PMC11621679 39639009

[60] Qi, H. et al. Homogenizing energy landscape for efficient and spectrally stable blue perovskite light-emitting diodes. *Adv. Mater.* **36**, e2409319 (2024). 10.1002/adma.202409319 39302002

[61] Nong, Y. et al. Boosting external quantum efficiency of blue perovskite QLEDs exceeding 23% by trifluoroacetate passivation and mixed hole transportation design. *Adv. Mater.* **36**, e2402325 (2024). 10.1002/adma.202402325 38631673

[62] Liu, A. et al. Optimizing perovskite surfaces to enhance post-treatment for efficient blue mixed-halide perovskite light-emitting diodes. *Adv. Mater.* **37**, e2414788 (2024). 10.1002/adma.202414788 PMC12204133 39632461

[63] Tong, Y. F. et al. In situ halide exchange of cesium lead halide perovskites for blue light-emitting diodes. *Adv. Mater.* **35**, e2207111 (2023). 10.1002/adma.202207111 36305014

[64] Sun, Y. Q. et al. Bright and stable perovskite light-emitting diodes in the near-infrared range. *Nature* **615**, 830–835 (2023). 10.1038/s41586-023-05792-4 36922588

[65] Li, M. et al. Acceleration of radiative recombination for efficient perovskite LEDs. *Nature* **630**, 631–635 (2024). 10.1038/s41586-024-07460-7 PMC11186751 38811739

[66] Zou, C., Liu, Y., Ginger, D. S. & Lin, L. Y. Suppressing efficiency roll-off at high current densities for ultra-bright green perovskite light-emitting diodes. *ACS Nano* **14**, 6076–6086 (2020). 10.1021/acsnano.0c01817 32324379

[67] de Mello, J. C., Wittmann, H. F. & Friend, R. H. An improved experimental determination of external photoluminescence quantum efficiency. *Adv. Mater.* **9**, 230–232 (1997).

[68] Egerton, R. F. Dose measurement in the TEM and STEM. *Ultramicroscopy* **229**, 113363 (2021). 10.1016/j.ultramic.2021.113363 34343770

[69] Cautaerts, N. et al. Free, flexible and fast: orientation mapping using the multi-core and GPU-accelerated template matching capabilities in the Python-based open source 4D-STEM analysis toolbox Pyxem. *Ultramicroscopy* **237**, 113517 (2022). 10.1016/j.ultramic.2022.113517 35427885

