# Supplementary material for: Interface-regulated vapour crystallization for ultrapure perovskite LEDs
Source: Nat Synth. 2026 Jun 22;5(11):1712–23. doi: 10.1038/s44160-026-01100-w (PMC13739748; doi:10.1038/s44160-026-01100-w)
Supplement: Supplementary file 1 [file 44160_2026_1100_MOESM1_ESM.pdf]

nature synthesis

Supplementary information https://doi.org/10.1038/s44160-026-01100-w

Interface-regulated vapour crystallization for ultrapure perovskite LEDs

In the format provided by the authors and unedited

Table of Contents

Supplementary Fig. 1 | PL spectra of 35%Cl perovskite films after DMF vapor treatment

Supplementary Fig. 2 | SEM micrographs of 35%Cl perovskite films

Supplementary Fig. 3 | SEM micrographs of 48%Cl and 35%Cl perovskite films

Supplementary Fig. 4 | Hyperspectral PL spectroscopy of vapor-treated 35%Cl on SiN_x/PVK and SiN_x/PVP

Supplementary Fig. 5 | STEM of post-treated 35%Cl perovskite on SiN_x/PVK

Supplementary Fig. 6 | STEM of post-treated 35%Cl perovskite on SiN_x/PVP

Supplementary Fig. 7 | STEM of post-treated 35%Cl perovskite on SiN_x/PVP

Supplementary Fig. 8 | 2D GIWAXS plot and GIWAXS patterns of 35%Cl perovskite films on ITO/PVK

Supplementary Fig. 9 | 2D GIWAXS plot and GIWAXS patterns of 35%Cl perovskite films on ITO/PVP

Supplementary Fig. 10 | 2D GIWAXS plot of 35%Cl perovskite films on ITO/PVK and ITO/PVP

Supplementary Fig. 11 | FTIR spectra

Supplementary Fig. 12 | Conductive AFM of post-treated 35%Cl perovskite

Supplementary Fig. 13 | PL spectra of 35%Cl perovskite without annealing

Supplementary Fig. 14 | Hyperspectral PL microscopy of 35%Cl perovskite without vapor treatment

Supplementary Fig. 15 | Understanding the negligible leakage current in the device

Supplementary Fig. 16 | Device performance of 35%Cl PeLEDs based on NiO_x

Supplementary Fig. 17 | Device performance of 35%Cl PeLEDs as a function of PVP

Supplementary Fig. 18 | Fluence-dependent external PLQY of vapor-treated 35%Cl perovskite films deposited on different underlying materials

Supplementary Fig. 19 | 3D-FDTD simulation of light outcoupling of PeLED

Supplementary Fig. 20 | SEM micrographs of 35%Cl perovskite with different film coverage

Supplementary Fig. 21 | AFM images of 35%Cl perovskite with different film coverage

Supplementary Fig. 22 | Device performance of 35%Cl PeLEDs with different film coverage

Supplementary Fig. 23 | EL spectral stability of 35%Cl PeLEDs

Supplementary Fig. 24 | Pulse operation of 35%Cl PeLEDs

Supplementary Fig. 25 | PL spectra of 0%Cl perovskite after post-treatment

Supplementary Fig. 26 | SEM image of vapor-treated 0%Cl perovskite on ITO/PVK

Supplementary Fig. 27 | EL spectral stability of 0%Cl PeLEDs

Supplementary Fig. 28 | EL spectral stability of 40%Cl PeLEDs

Supplementary Fig. 29 | EL spectral stability of 48%Cl PeLEDs

Supplementary Fig. 30 | EL spectra of 48%Cl, 40%Cl, 35%Cl, and 0%Cl PeLEDs

Supplementary Fig. 31 | PL spectra of 35%Cl perovskite with a film coverage of ~61%

Supplementary Fig. 32 | External PLQY of 35%Cl perovskite with different film coverage

Supplementary Fig. 33 | PL spectral stability of 35%Cl perovskite thin films

Supplementary Fig. 34 | Device performance of 35%Cl PeLEDs without vapor exposure

Supplementary Fig. 35 | GIWAXS pattern of 35%Cl perovskite films with different film coverage

Supplementary Fig. 36 | XPS analysis of 35%Cl perovskite on ITO after vapor exposure for 12 minutes

Supplementary Fig. 37 | SEM images of ITO/PVK before and after DMF vapor treatment

Supplementary Fig. 38 | XRD of 35%Cl perovskite on ITO after vapor exposure for 12 minutes

Supplementary Fig. 39 | Time-resolved PL (TRPL) of vapor-treated perovskite on ITO/PVK and ITO/PVP

Supplementary Fig. 40 | PL spectra of 70%I (30%Br) perovskite on ITO/PVK after DMF vapor treatment

Supplementary Fig. 41 | Logarithm plot of the integrated PL intensity versus laser intensity

Supplementary Note 1 | Morphology change of perovskite on different underlying materials upon vapor treatment

Supplementary Note 2 | Discussion of the excitonic absorption peak of post-treated perovskite

Supplementary Note 3 | Perovskite light-emitting diodes (PeLEDs) design and optimization

Supplementary Note 4 | Outcoupling efficiency simulation

Supplementary Note 5 | Discussion of our strategy and the stability of the device

Supplementary Table 1 | Estimated attenuation length of X-ray at different incident angles

Supplementary Table 2 | Summary of the state-of-the-art blue PeLEDs

Supplementary Table 3 | Summary of the state-of-the-art blue QLEDs

Supplementary Table 4 | Summary of the state-of-the-art blue OLEDs

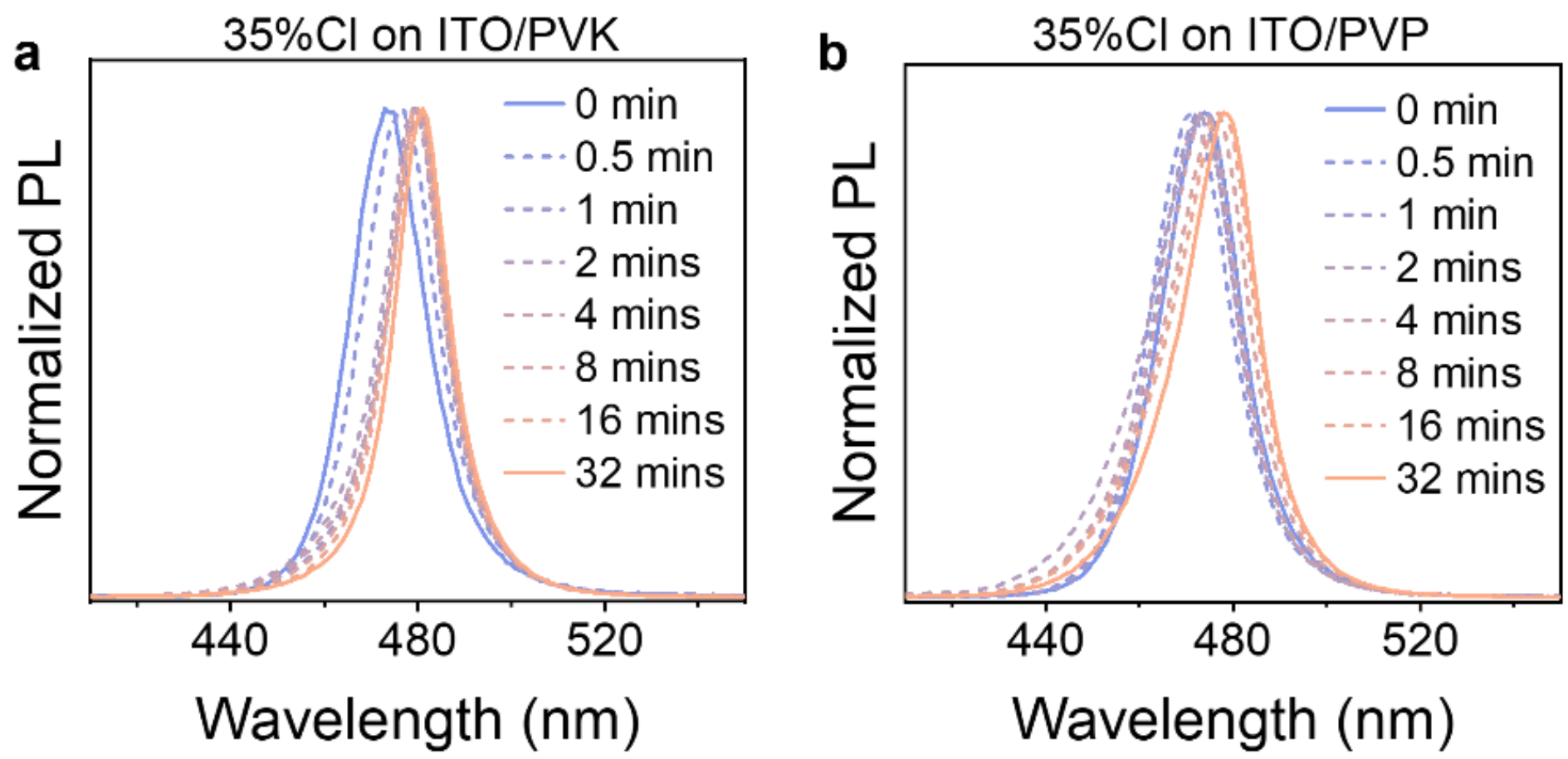

Supplementary Fig. 1 | PL spectra of 35%Cl perovskite films after DMF vapor treatment. **a**, on ITO/PVK. **b**, on ITO/PVP. 0 min indicates no vapor treatment. Upon vapor treatment, the perovskite films were subsequently annealed at 80 °C for 8 minutes to remove residual solvent.

The PL peak of the perovskite films on ITO/PVK undergoes a gradual redshift from 473 nm (without DMF vapor treatment) to 481 nm after extended exposure to DMF vapor (32 mins). Concurrently, the FWHM decreases monotonically from 18.8 nm to 13.8 nm. In contrast, the PL spectra of perovskite films on ITO/PVP exhibit an initial blueshift from 474 nm to 471 nm within the first 0.5 minutes of DMF vapor treatment, followed by a redshift to 478 nm after 32 minutes. Beyond the spectral shift, the PL emission from perovskite on ITO/PVP becomes increasingly asymmetric over time, characterized by a pronounced high-energy shoulder.

The PL redshift after DMF vapor treatment should arise from the redistribution of both A-site cation and X-site halide during vapor-assisted crystallization. Without vapor treatment, rapid thermal annealing of this mixed-composition perovskite leads to a heterogeneous perovskite film containing both large bandgap and small bandgap domains, as supported by the energy transfer from ~460 nm to ~480 nm in TA (Extended Data Fig. 2a-b) as well as the spatially varying PL peak (470-480 nm) in hyperspectral PL (Supplementary Fig. 14). The large bandgap domain should be Cl-rich and Cs/Rb-rich phases, which are more likely located near the bottom of the perovskite film. This assignment is supported by the larger *q*-value observed in incident angle-dependent GIWAXS (Fig. 2a) at a relatively large incident angle (~0.4°), together with the stronger Cs^+ and Rb^+ signals detected near the bottom of the film in the TOF-SIMS depth profile in Fig. 2e. After DMF vapor treatment, these large bandgap domains are effectively suppressed, resulting in a more homogeneous perovskite film dominated by a uniform smaller bandgap perovskite phase, which in turn leads to the observed PL redshift. The disappearance of the large bandgap domains is evidenced by the negligible energy transfer in TA (Extended Data Fig. 2e-f) and by the nearly identical PL peak across different regions in hyperspectral PL imaging (Fig. 1b). Moreover, the suppression of the large bandgap domains should originate from the incorporation of additional Br^- and/or FA^+ into the perovskite lattice during DMF vapor treatment, therefore giving rise to a more uniform lattice spacing throughout the film thickness (Fig. 2d). Although TOF-SIMS shows that FA^+ is mainly enriched near the grain surface, we believe that a fraction of FA^+ should be incorporated into the perovskite lattice, especially in the 0%Cl perovskite system. Otherwise, the PL spectra of pure bromide perovskite system should remain unchanged after DMF vapor treatment.

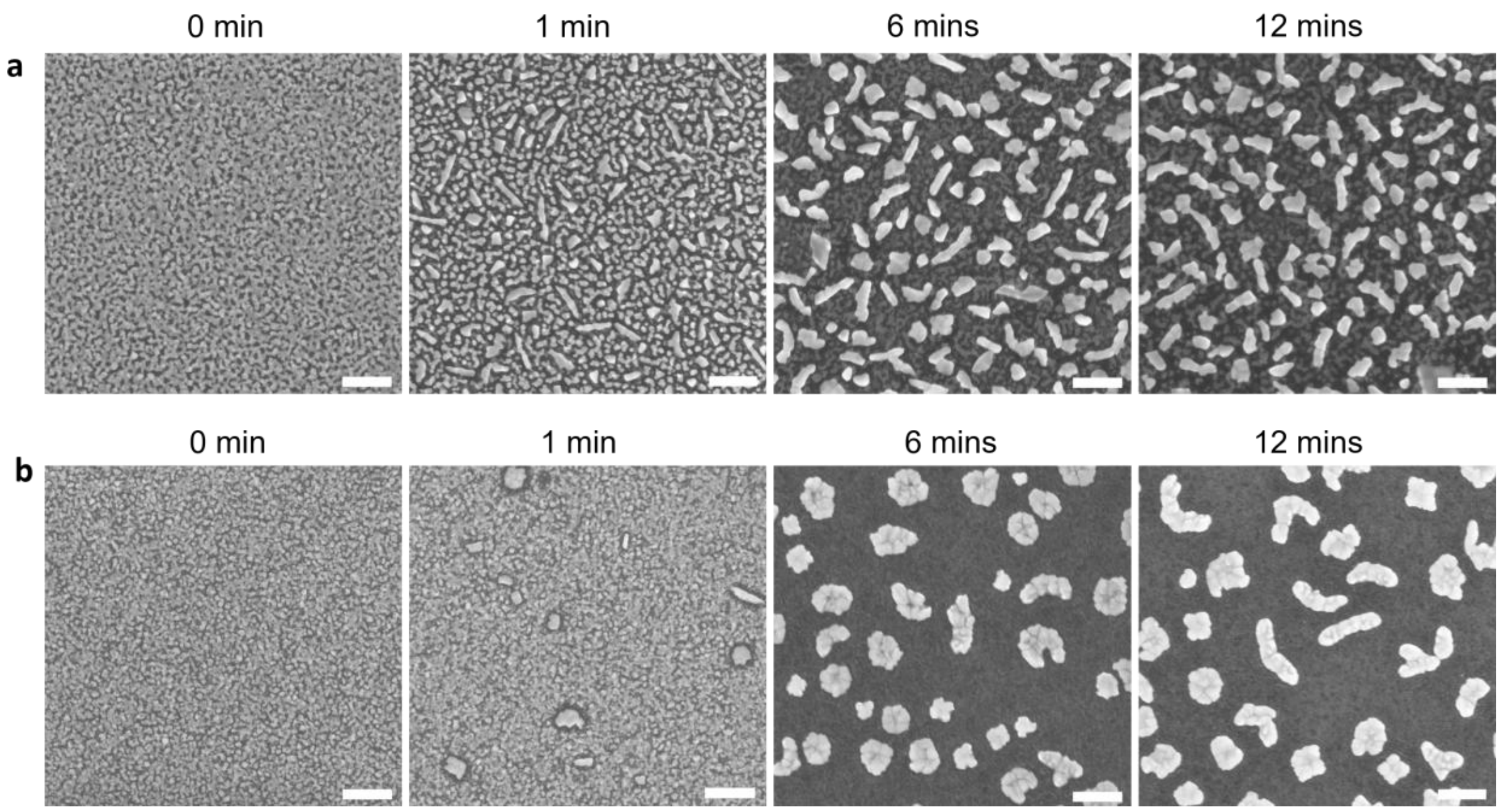

Supplementary Fig. 2 | SEM micrographs of 35%Cl perovskite films. a, on ITO/PVK after DMF vapor exposure for 0 minutes, 1 minute, 6 minutes, and 12 minutes. **b**, on ITO/PVP after DMF vapor exposure for 0 minutes, 1 minute, 6 minutes, and 12 minutes. The scale bar is 500 nm.

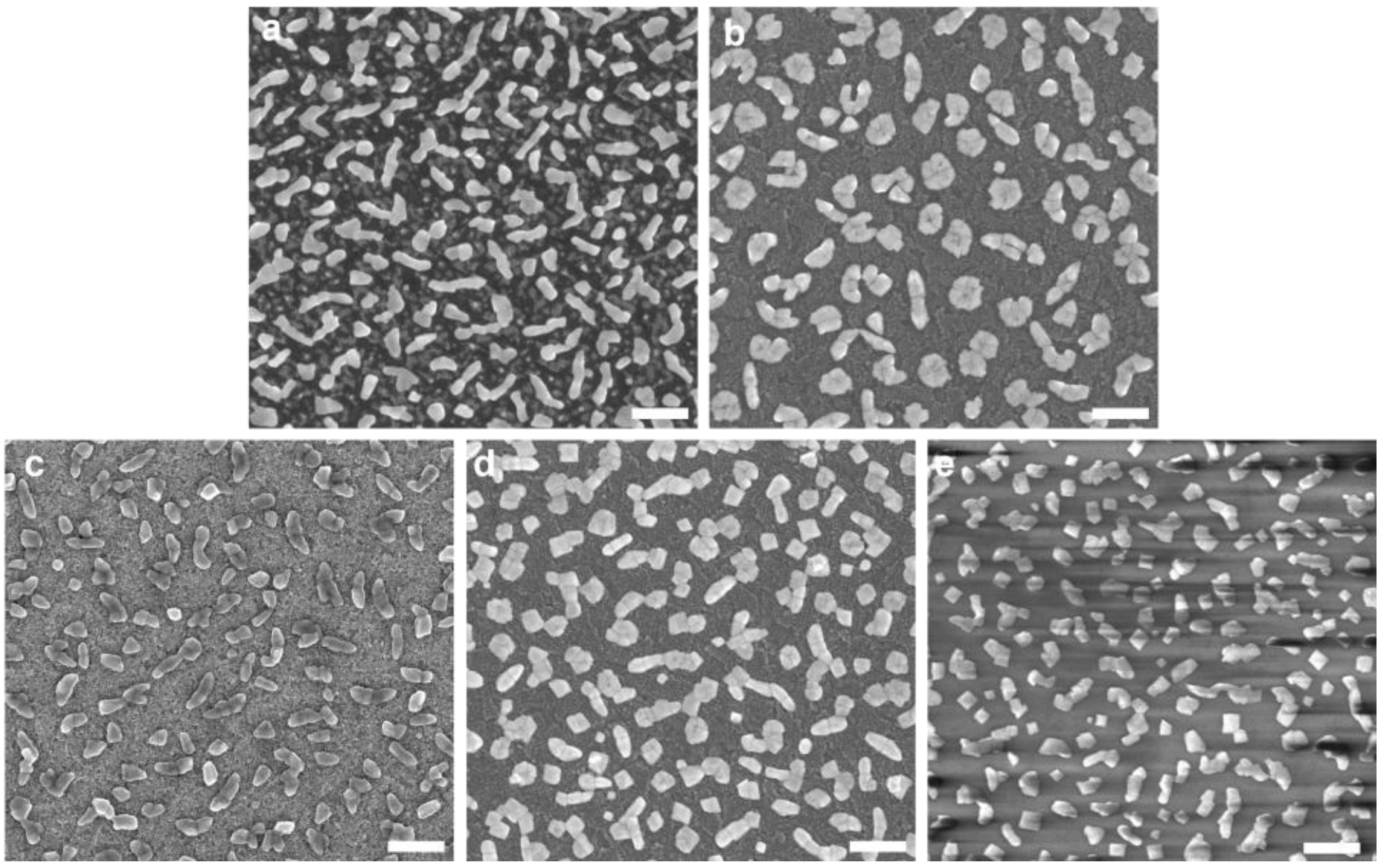

Supplementary Fig. 3 | SEM micrographs of 48%Cl and 35%Cl perovskite films. a, 48%Cl perovskite on ITO/PVK after 20 minutes of DMF vapor treatment. **b**, 48%Cl perovskite on ITO/PVP after 20 minutes of DMF vapor treatment. **c**, 35%Cl perovskite on ITO/NiO_x after 12 minutes of DMF vapor treatment. **d**, 35%Cl perovskite on ITO after 12 minutes of DMF vapor treatment. **e**, 35%Cl perovskite on glass after 12 minutes of DMF vapor treatment. The scale bar is 500 nm. The dark region in (**e**) is electron charging due to the poor conductivity of glass.

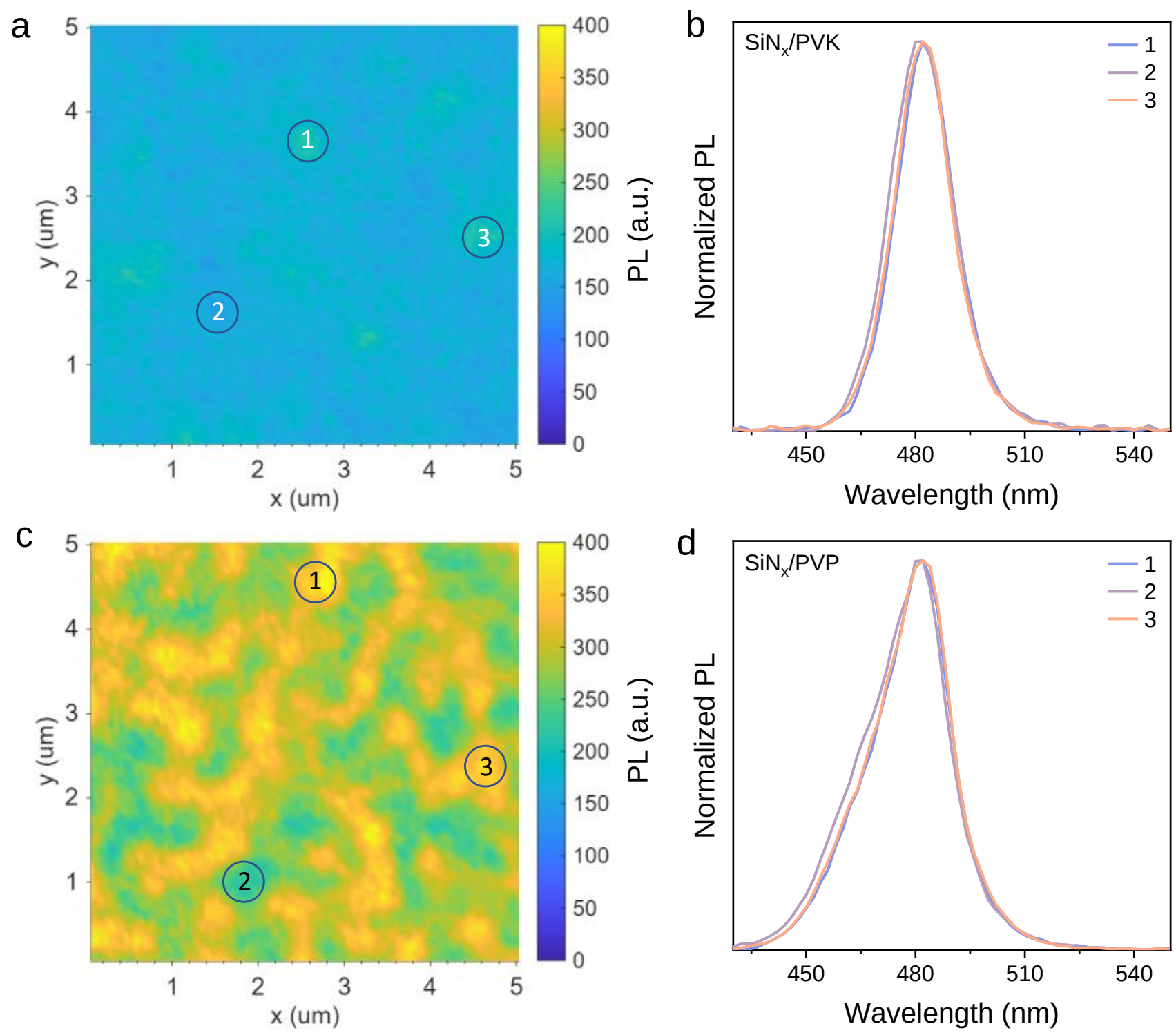

Supplementary Fig. 4 | Hyperspectral PL spectroscopy of vapor-treated 35%Cl on SiN$_x$/PVK and SiN$_x$/PVP. a. Hyperspectral PL intensity map (excitation at 405 nm) of vapor-treated 35%Cl on SiN$_x$/PVK. **b**, PL spectra of randomly selected three regions in (**a**). **c**. Hyperspectral PL intensity map of vapor-treated 35%Cl on SiN$_x$/PVP. **d**, PL spectra of randomly selected three regions in (**c**).

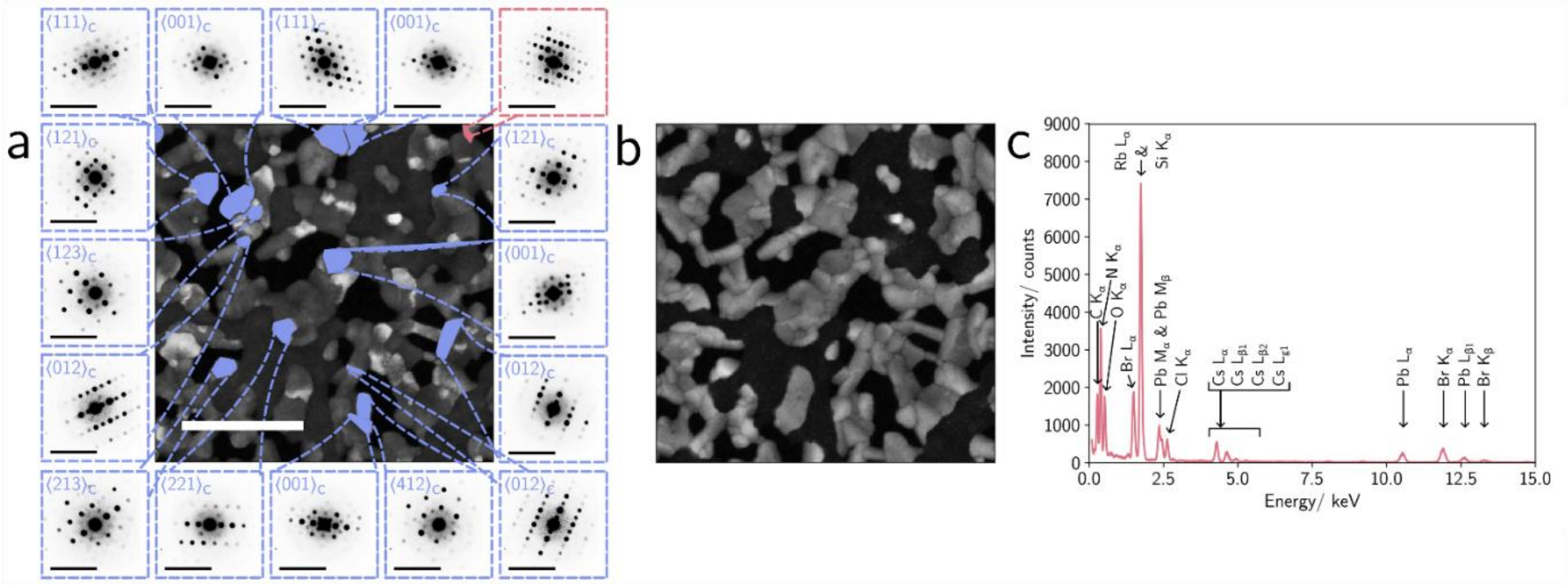

Supplementary Fig. 5 | STEM of post-treated 35%Cl perovskite on SiN_x/PVK. a, Virtual darkfield image reconstructed from the scanning electron diffraction (SED) data, formed with an inner and outer annular radius of 0.18 $Å^{-1}$ and 1.08 $Å^{-1}$. Around the image are diffraction patterns that are indexed to an approximate zone axis using a pseudo-cubic unit cell (blue), minimal phase impurities are also present which cannot be indexed to a pseudo-cubic unit cell (red). **b**, A HAADF image of the same area shown in (a). **c**, A summed energy dispersive X-ray spectra from the sample. Scale bars are 300 nm and 1$Å^{-1}$. **a** & **b** were recorded with a D_f of ~11 $e^- Å^{-2}$. (c) was recorded with a D_f of ~4500 $e^- Å^{-2}$.

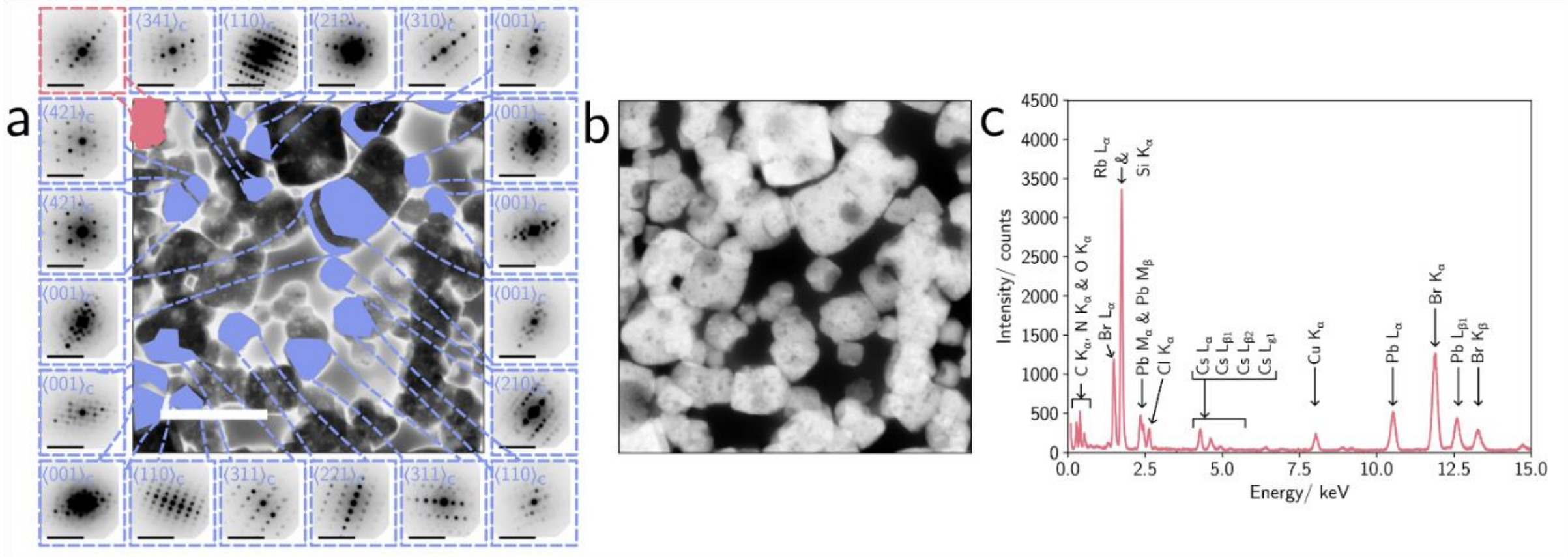

Supplementary Fig. 6 | STEM of post-treated 35%Cl on SiN$_x$/PVP. a, A virtual darkfield image reconstructed from the SED data, formed with an inner and outer annular radius of **s** = 0.18 Å^{-1} and 1.08 Å^{-1}. Around the image are diffraction patterns that are indexed to an approximate zone axis using a pseudo-cubic unit cell (blue), minimal phase impurities are also present which cannot be indexed to a pseudo-cubic unit cell (red). **b**, A HAADF image of the same area shown in (a). **c**, A summed energy dispersive X-ray spectra from the sample. Scale bars are 1 µm and **s** = 1 Å^{-1}. **a** & **b** were recorded with a D_f of ~22 e$^-$ Å^{-2}. **c** was recorded with a D_f of 7250 e$^-$ Å^{-2}.

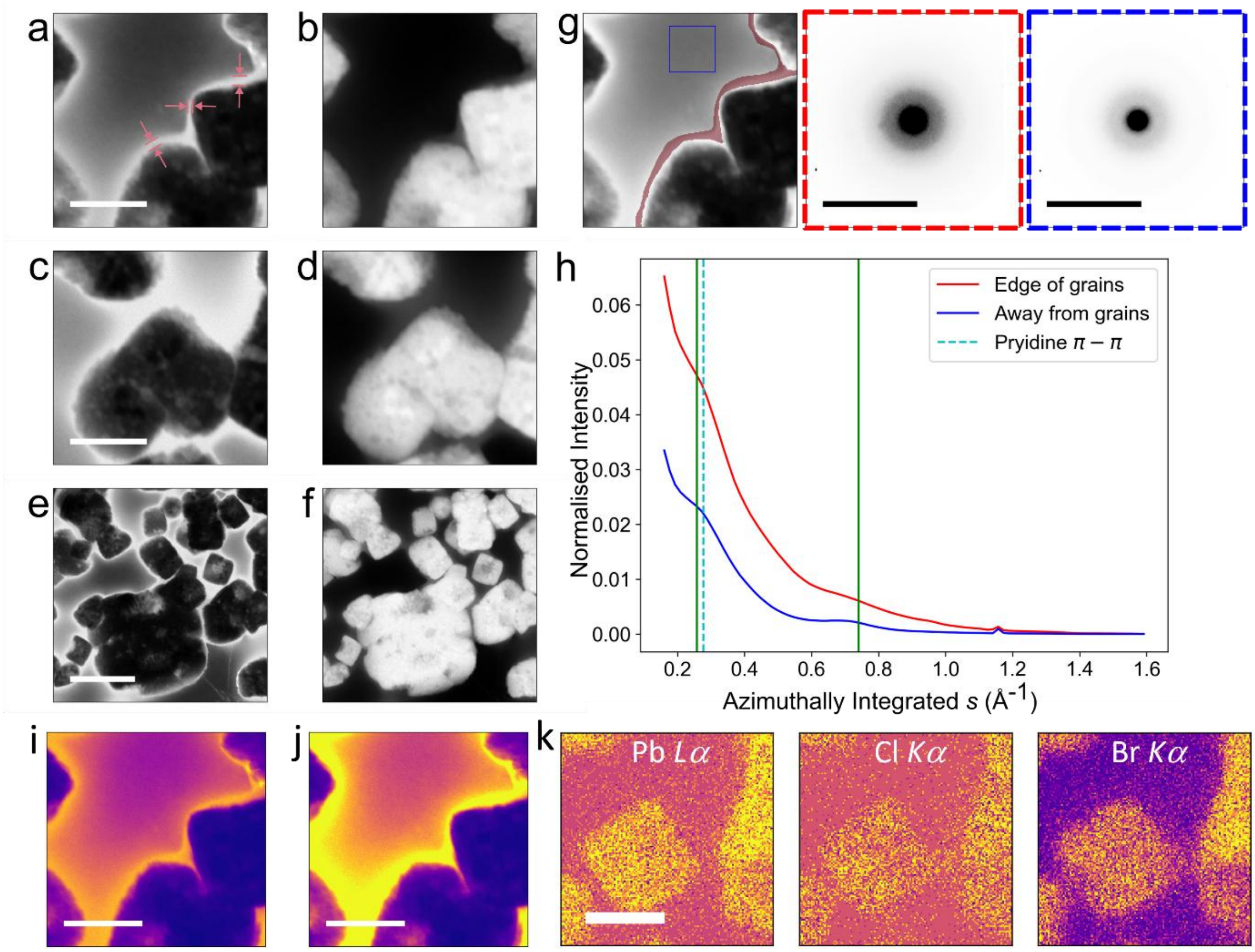

Supplementary Fig. 7 | STEM of post-treated 35%Cl on SiN_x/PVP. Virtual darkfield images (**a**, **c**, **e**) reconstructed from the SED data, formed with an inner and outer annular radius of **s** = 0.18 $Å^{-1}$ and 1.08 $Å^{-1}$. HAADF images (**b**, **d**, **f**) of the same area shown in **a**, **c**, and **e**, respectively. **g**, A mask has been created with PVP surrounding grains shown in red and an area away from the edge of grains shown in blue. The mean diffraction patterns for each region are displayed. **h**, Azimuthally integrated intensities having been normalised from the red and blue regions shown in (**g**). Vertical lines show where scattering from the *π-π* stacking of pyridine[1] is expected as well as SiN_x (green). Scale bars are 300 nm (**a** and **c**); 1 µm (**e**) and **s** = 1 $Å^{-1}$. **a-d** were recorded with a D_f of ~11 $e^- Å^{-2}$ whereas **e** and **f** were recorded with D_f of ~1 $e^- Å^{-2}$ as the current was maintained at ~2 pA. Note this will not have lowered the D_p for **e** and **f**, as described in the Methods Section. Virtual darkfield images formed with an inner and outer annulus between **s** = 0.2 $Å^{-1}$ and 0.35 $Å^{-1}$, where scattering from PVP is expected given *π-π* stacking distances. (**i**) With no threshold applied, (**j**) With a threshold of 70% of the maximum value applied. Scale bars: 300 nm. **k**, Integrated EDX signal from the Pb *Lα*, Cl *Kα*, and Br *Kα* peaks after background subtraction. Cumulative electron fluence (D_f) of ~7250 $e^- Å^{-2}$. Scale bar: 300 nm. This amorphous layer highlighted in red in Supplementary Fig. 7a, 7g is consistent with PVP polymer, with increased scattering at a **s** vector around ~0.28 $Å^{-1}$ (pyridine *π-π* stacking expected ~3.6 Å)[1] evidenced from azimuthally integrated intensities (Supplementary Fig. 7h) and virtual darkfield images (Supplementary Fig. 7i-j).

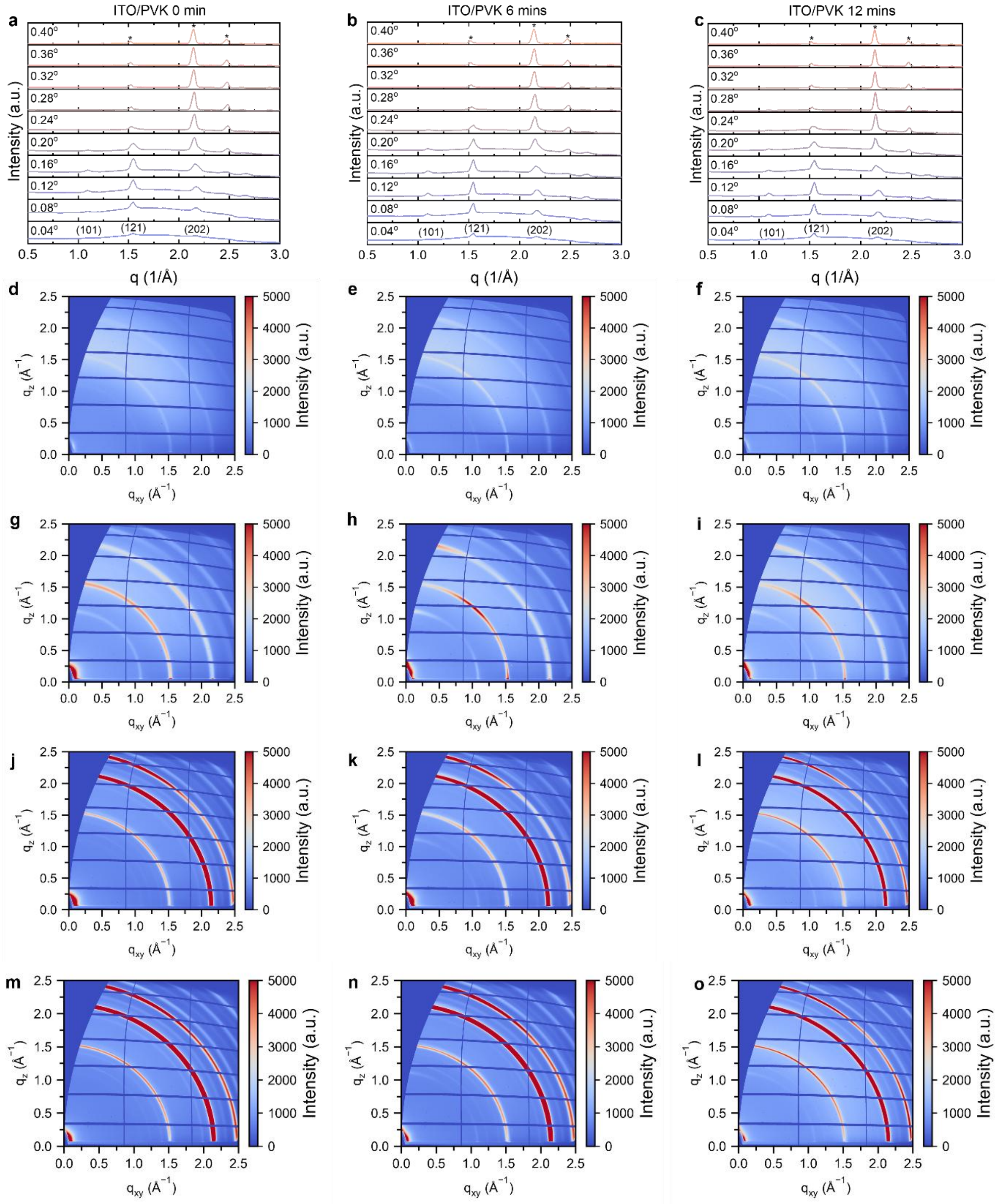

Supplementary Fig. 8 | 2D GIWAXS plot and GIWAXS patterns of 35%Cl perovskite films on ITO/PVK. a, incident angle-dependent 2D GIWAXS plot of 35%Cl perovskite on ITO/PVK without treatment and their GIWAXS patterns at an incident angle of 0.04^{o} (**d**), 0.16^{o} (**g**), 0.28^{o} (**j**), and 0.40^{o} (**m**). **b**, incident angle-dependent 2D GIWAXS plot of 35%Cl perovskite on ITO/PVK after 6 minutes of treatment and their GIWAXS patterns at an incident angle of 0.04^{o} (**e**), 0.16^{o} (**h**), 0.28^{o} (**k**), and 0.40^{o} (**n**). **c**, incident angle-dependent 2D GIWAXS plot of 35%Cl perovskite on ITO/PVK after 12 minutes of treatment and their GIWAXS patterns at an incident angle of 0.04^{o} (**f**), 0.16^{o} (**i**), 0.28^{o} (**l**), and 0.40^{o} (o). The * indicates ITO diffraction peaks.

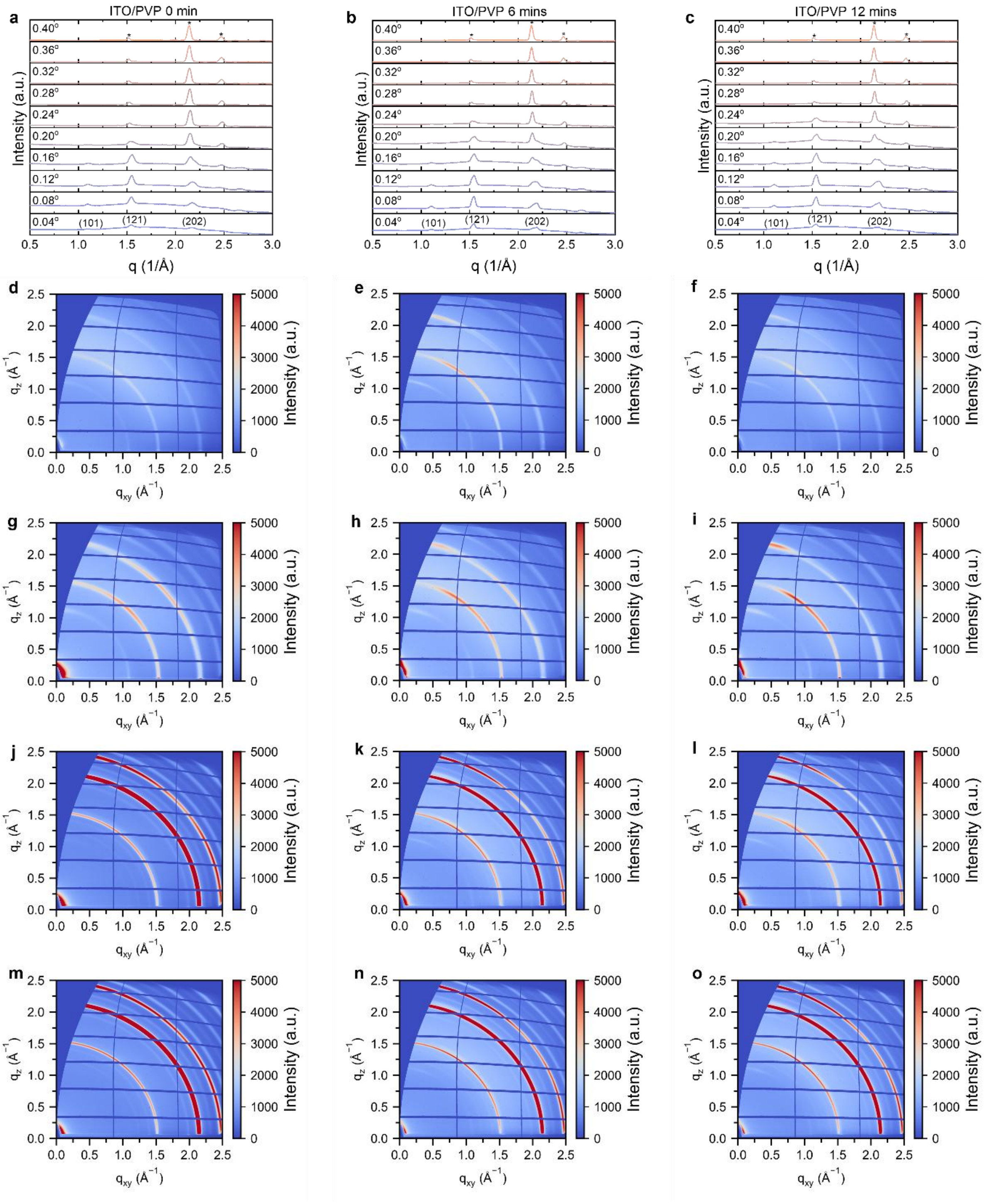

Supplementary Fig. 9 | 2D GIWAXS plot and GIWAXS patterns of 35%Cl perovskite films on ITO/PVP. a, incident angle-dependent 2D GIWAXS plot of 35%Cl perovskite on ITO/PVP without treatment and their GIWAXS patterns at an incident angle of 0.04º (**d**), 0.16º (**g**), 0.28º (**j**), and 0.40º (**m**). **b**, incident angle-dependent 2D GIWAXS plot of 35%Cl perovskite on ITO/PVP after 6 minutes of treatment and their GIWAXS patterns at an incident angle of 0.04º (**e**), 0.16º (**h**), 0.28º (**k**), and 0.40º (**n**). **c**, incident angle-dependent 2D GIWAXS plot of 35%Cl perovskite on ITO/PVP after 12 minutes of treatment and their GIWAXS patterns at an incident angle of 0.04º (**f**), 0.16º (**i**), 0.28º (**l**), and 0.40º (o). The * indicates ITO diffraction peaks.

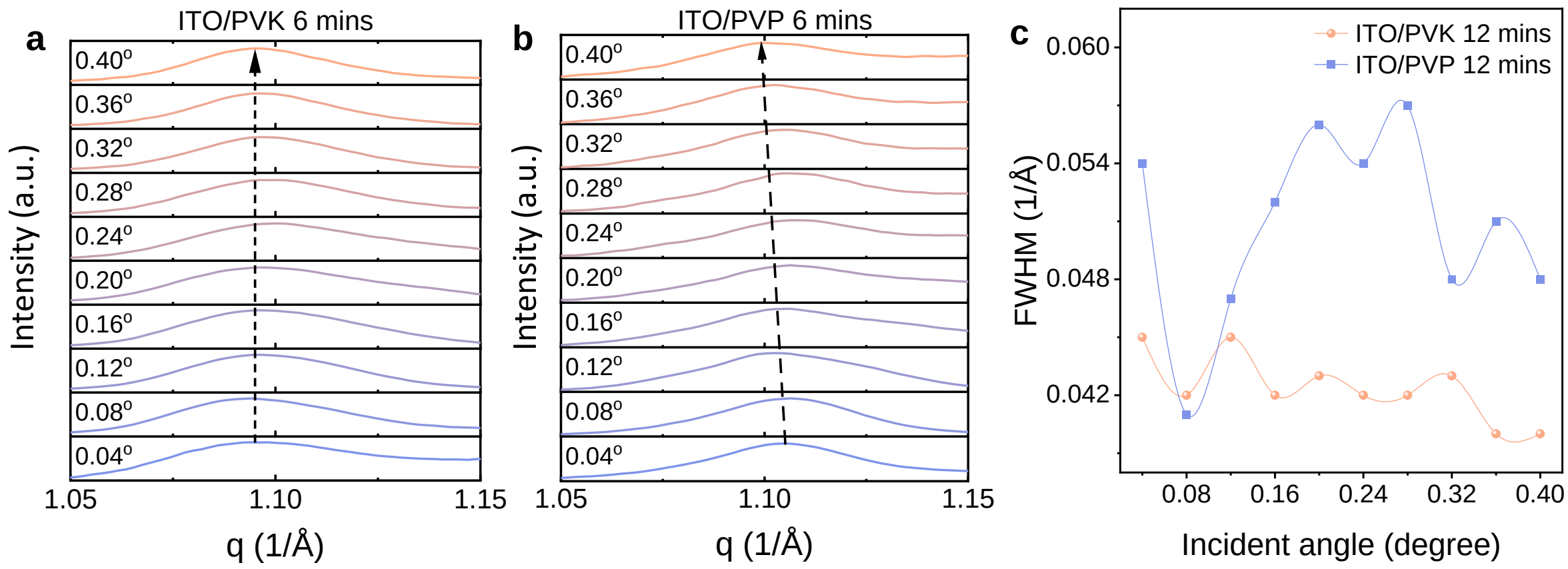

Supplementary Fig. 10 | 2D GIWAXS plot of 35%Cl perovskite films on ITO/PVK and ITO/PVP. a, 35%Cl perovskite on ITO/PVK after DMF vapor treatment for 6 minutes. **b**, 35%Cl perovskite on ITO/PVP after DMF vapor treatment for 6 minutes. **c**, extracted FWHM of the (101) plane of 35%Cl perovskite on ITO/PVK and ITO/PVP after vapor exposure for 12 minutes.

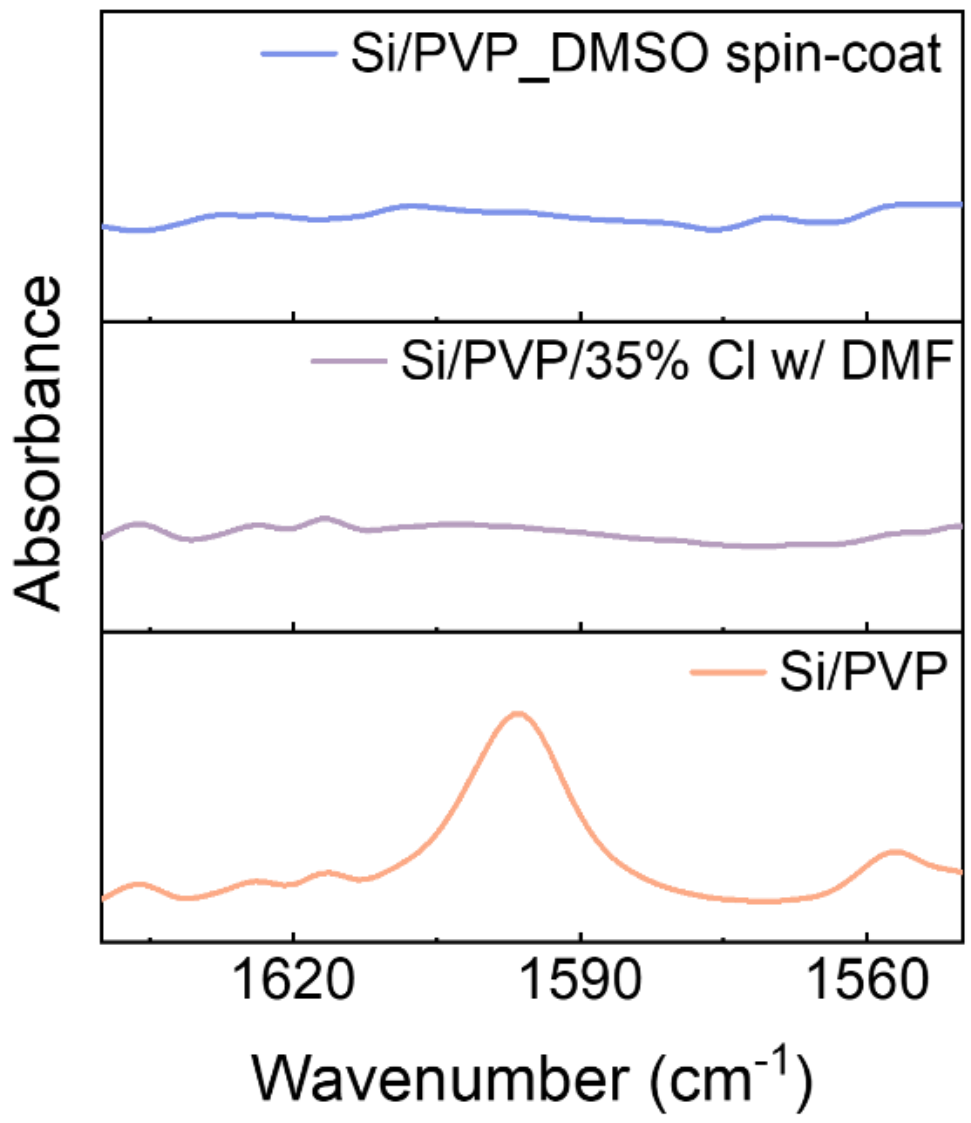

Supplementary Fig. 11 | FTIR spectra. FTIR spectra of PVP (6 mg/ml in IPA) on Si substrate before and after depositing perovskite, as well as PVP after spin coating pure DMSO.

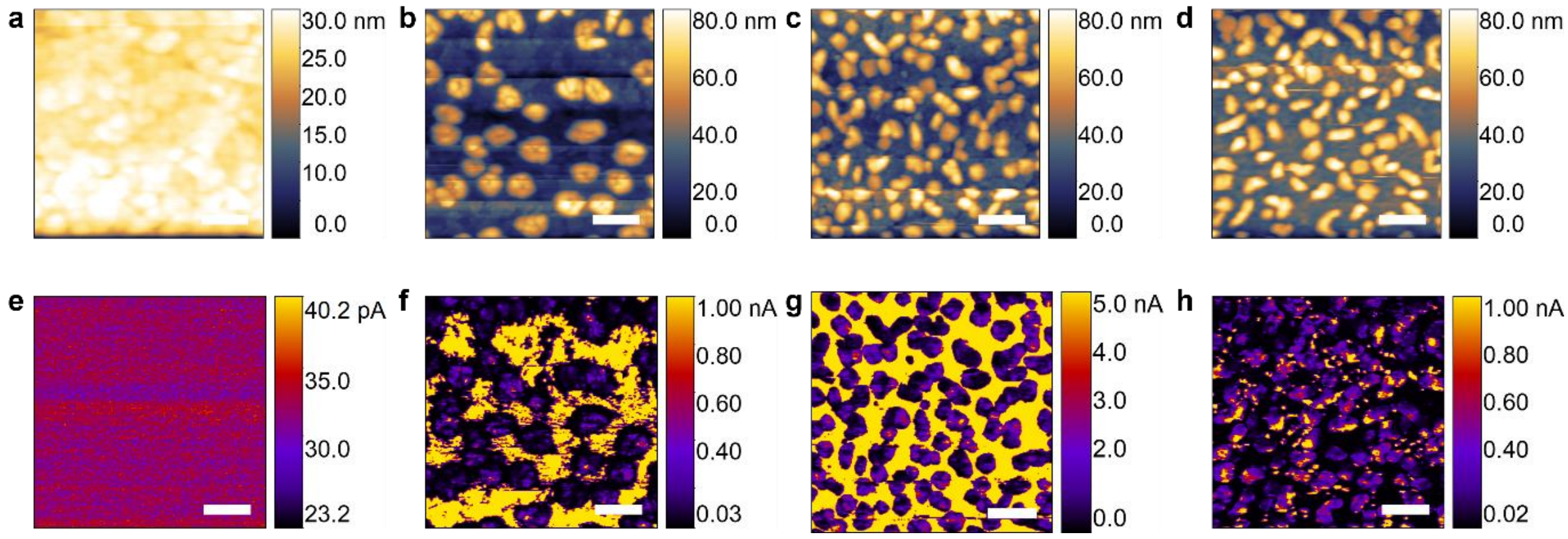

Supplementary Fig. 12 | Conductive AFM of post-treated 35%Cl perovskite. Topography (**a**) and current (**e**) map of ITO/PVP under a bias of 3 V. Topography (**b**) and current (**f**) map of 35%Cl on ITO/PVP after DMF vapor treatment for 12 minutes under a bias of 3 V. Topography (**c**) and current (**g**) map of 35%Cl on ITO after DMF vapor treatment for 12 minutes under a bias of 3 V. Topography (**d**) and current (**h**) map of 35%Cl on ITO/PVK after DMF vapor treatment for 12 minutes under a bias of 3 V. The scale bar is 500 nm.

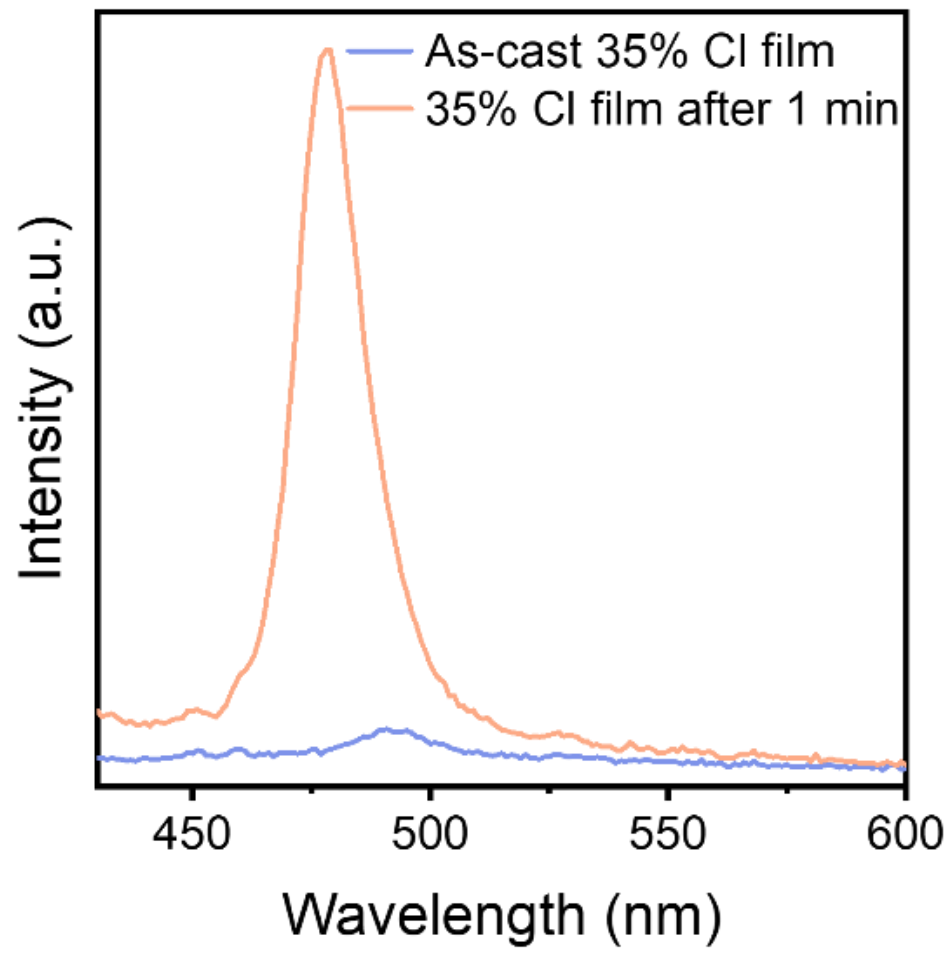

Supplementary Fig. 13 | PL spectra of 35%Cl perovskite without annealing. As-cast thin films and the films after 1 minute of DMF vapor exposure. Neither of these two films was thermally annealed. The negligible PL intensity under 400 nm excitation in as-cast films indicates that perovskite was not formed without thermal annealing.

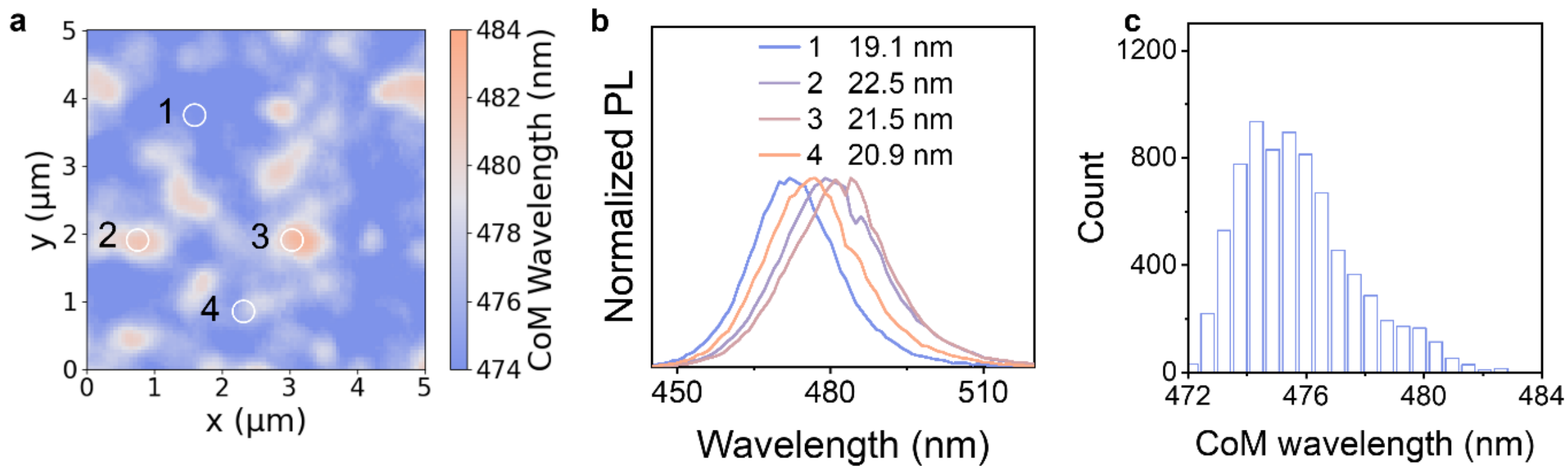

Supplementary Fig. 14 | Hyperspectral PL microscopy of 35%Cl perovskite without vapor treatment. Hyperspectral PL CoM peak map (**a**), PL spectra (**b**) of randomly selected four grains in (a), CoM histogram (**c**) of 35%Cl perovskite on ITO/PVK without DMF vapor treatment.

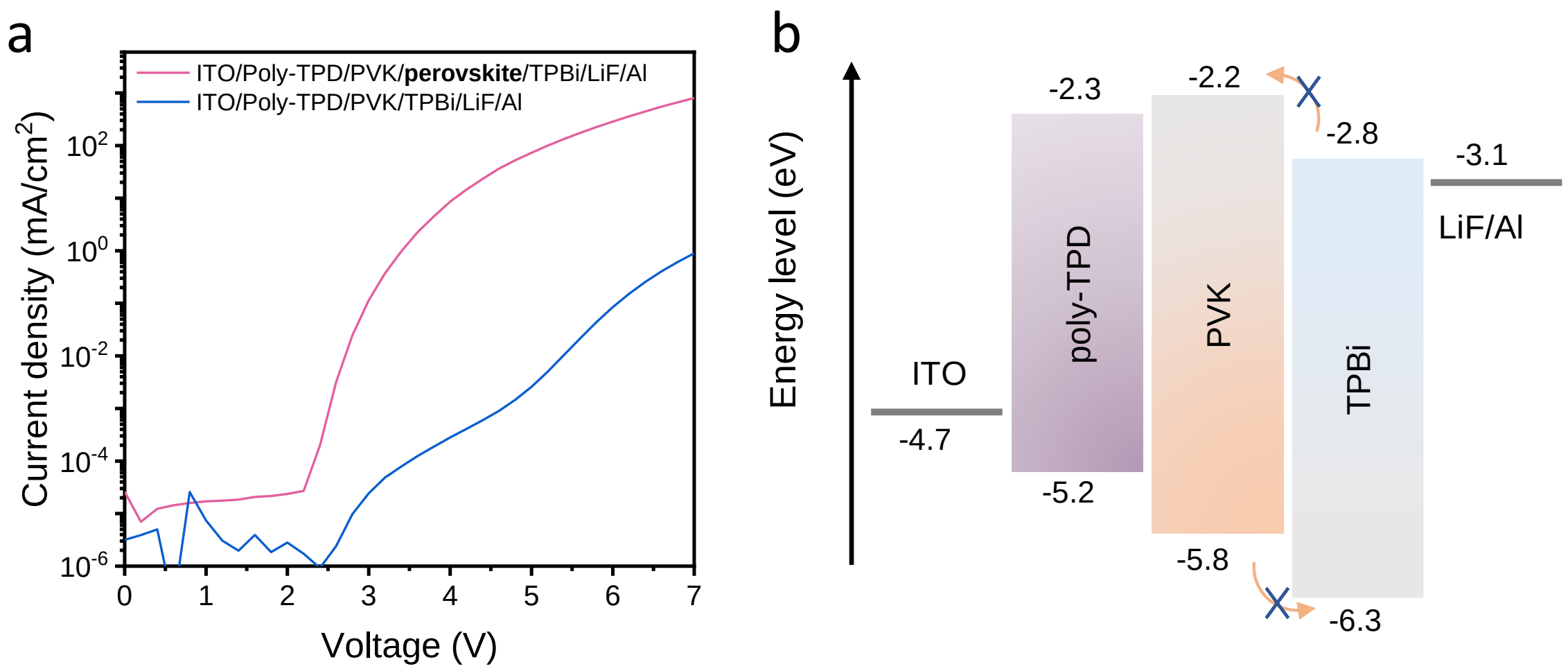

Supplementary Fig. 15 | Understanding the negligible leakage current in the device. a. Current density-voltage characteristics of devices with structures ITO/poly-TPD/PVK/perovskite/TPBi/LiF/Al or ITO/poly-TPD/PVK/TPBi/LiF/Al. **b.** Flat band energy level of the device with structure ITO/poly-TPD/PVK/TPBi/LiF/Al. The thickness of each layer in both devices was kept identical except for the 35%Cl perovskite layer. The energy level was taken from ref.2, ref.3, and ref.4.

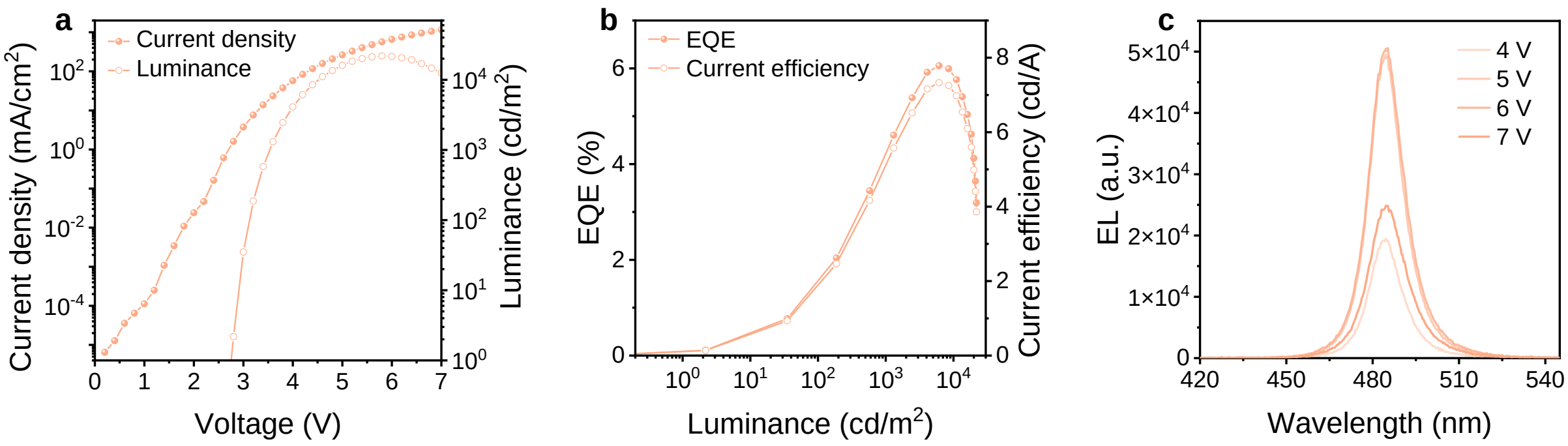

Supplementary Fig. 16 | Device performance of 35%Cl PeLEDs based on NiO_x. Voltage-current density-luminance characteristics (**a**), Luminance-EQE-current efficiency characteristics (**b**), EL spectra (**c**) of 35%Cl PeLEDs with a device structure of ITO/NiO_x/PVK/perovskite/TPBi/LiF/Al. Note that 1.5 mg/ml PVP was deposited on PVK but it was eventually dissolved in the perovskite precursors during perovskite fabrication. The perovskite was prepared using 0.08 M Pb.

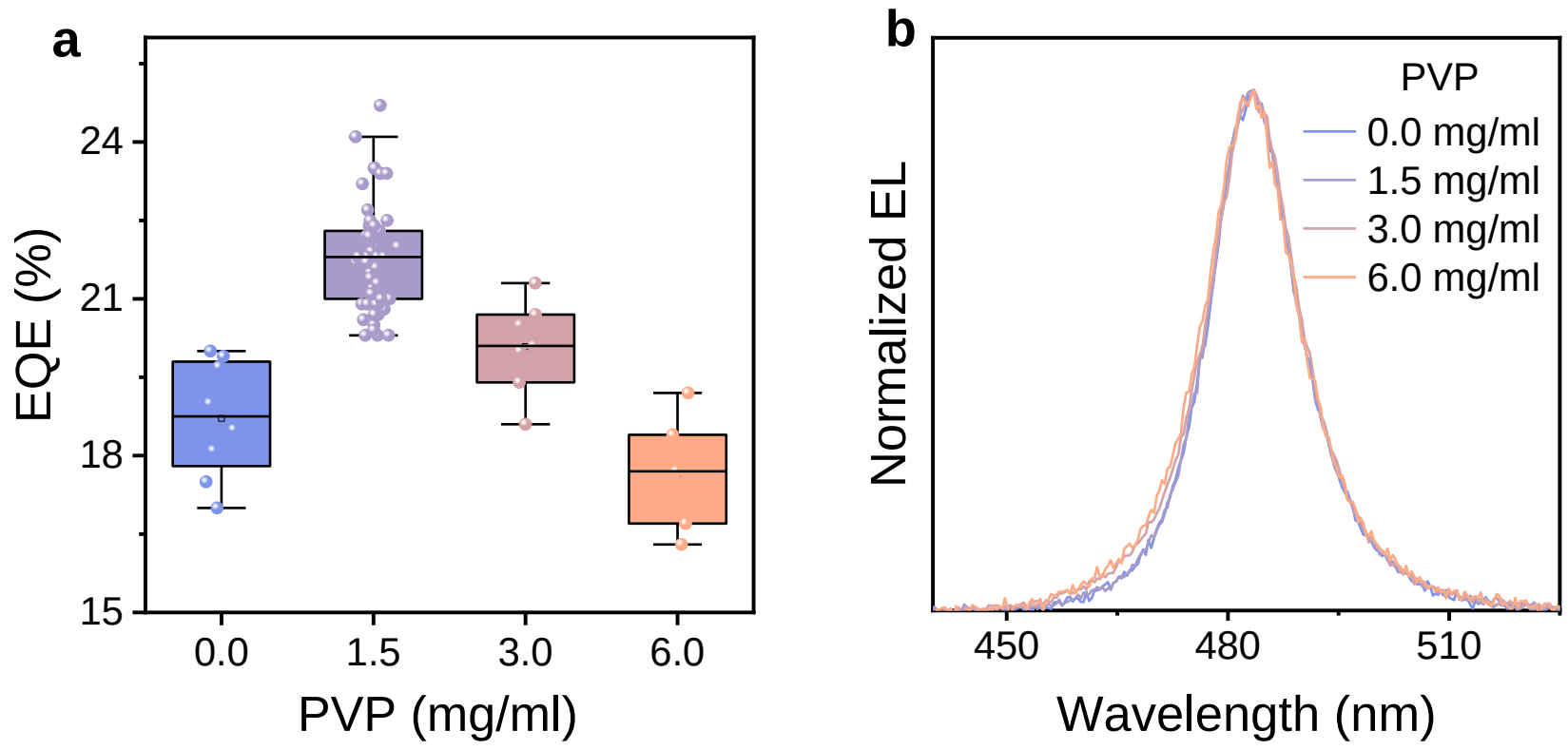

Supplementary Fig. 17 | Device performance of 35%Cl PeLEDs as a function of PVP. **a**, EQE histogram of 35%Cl PeLEDs with different concentrations of PVP. The number of PeLEDs is 8, 57, 7, and 5 for PVP with 0 mg/ml, 1.5 mg/ml, 3.0 mg/ml, and 6.0 mg/ml. **b**, EL spectra of 35%Cl PeLEDs with different concentrations of PVP under a bias voltage of 6 V. The device structure is ITO/poly-TPD/PVK/perovskite/TPBi/LiF/Al. The perovskite thin films were after DMF vapor treatment for 12 minutes. Clear emission on the high energy side is observed after increasing the PVP concentration, indicating the formation of large-bandgap domains. Nevertheless, the relative intensity of this high-energy emission in EL of the device is substantially weaker than that observed in the corresponding PL spectra of perovskite thin films on ITO/PVP. We attribute this difference primarily to inefficient carrier injection into the large bandgap domains during device operation. As a result, under electrical excitation, carriers preferentially recombine in the lower bandgap regions which dominate the EL spectrum.

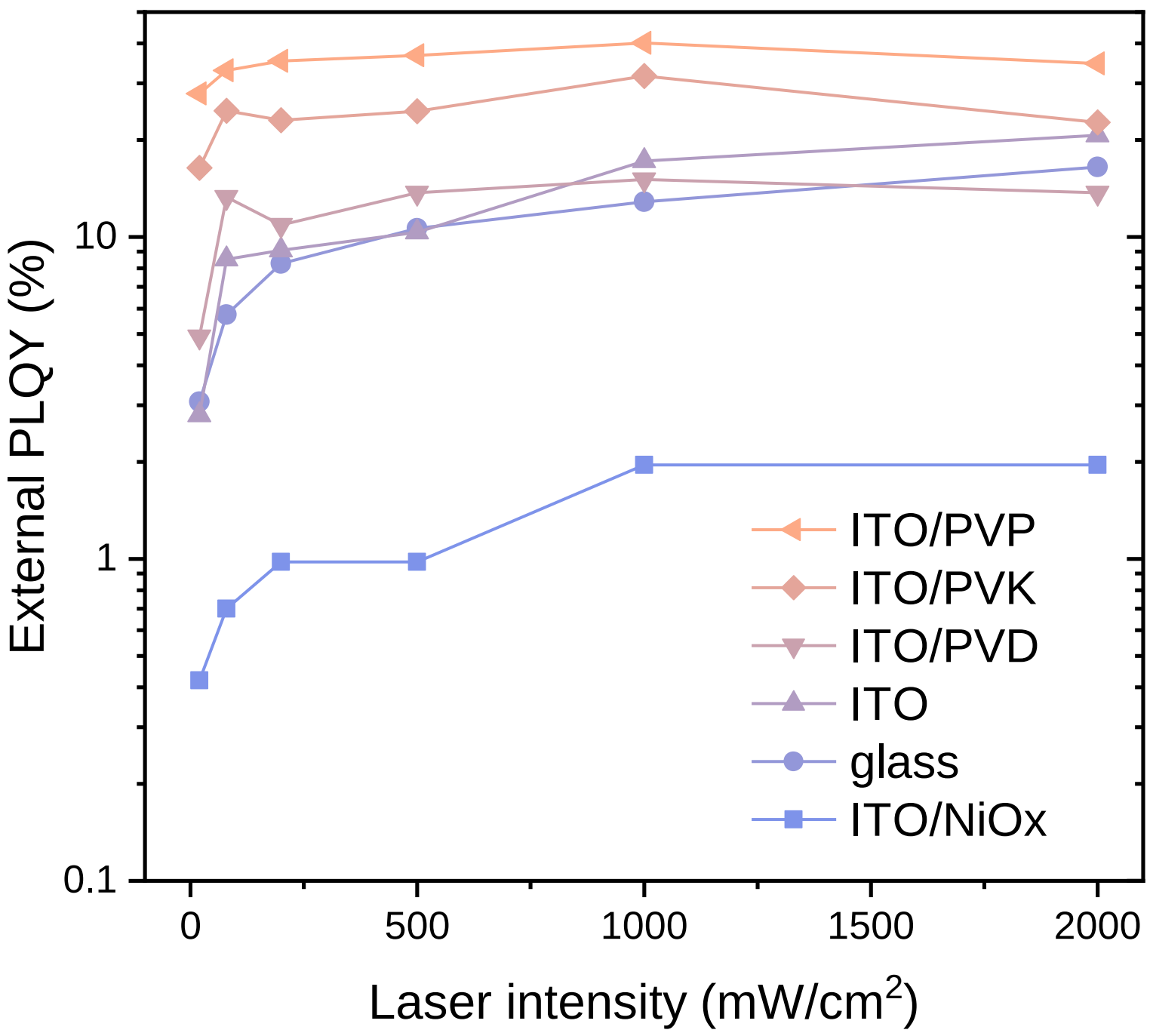

Supplementary Fig. 18 | Fluence-dependent external PLQY of vapor-treated 35%Cl perovskite films deposited on different underlying materials.

First, the external PLQY of the perovskite films gradually increases with increasing excitation intensity. This excitation fluence-dependent PLQY behavior has been widely reported in halide perovskite thin films and is commonly attributed to trap filling under illumination[5]. Second, the perovskite film on ITO/NiO_x exhibits the lowest external PLQY (1-2%), whereas the perovskite film on ITO/PVP shows the highest external PLQY (30-40%) among all samples. Notably, this trend differs from that of the FWHM shown in Fig. 1a, indicating that PLQY and FWHM are different figures of merit of thin films. The particularly low external PLQY of the perovskite film on ITO/NiO_x should be due to PL quenching induced by the NiO_x underlayer. The high external PLQY of the perovskite film on ITO/PVP was attributed to defect passivation by PVP owing to its strong coordination with perovskite precursors, as supported by the FTIR results in Fig. 3b and XPS analysis in Fig. 3c. Moreover, the higher external PLQY of the perovskite film on ITO/PVP compared with that on ITO/PVK is consistent with the improved EQE observed in PeLEDs employing an optimal concentration of PVP, as shown in Supplementary Fig. 17.

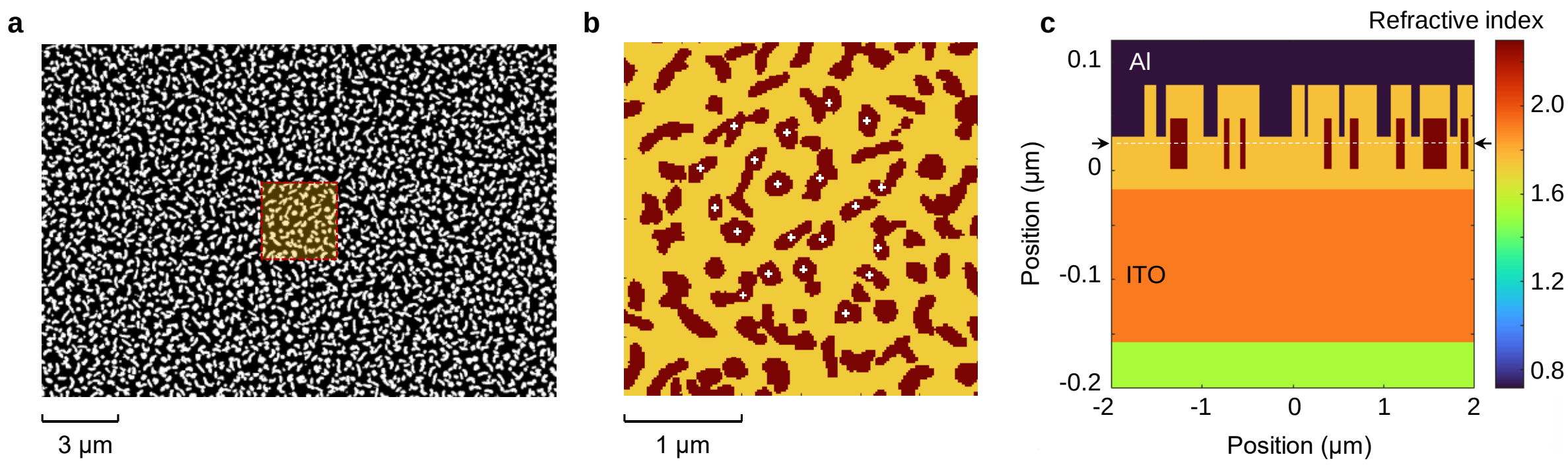

Supplementary Fig. 19 | 3D-FDTD simulation of light outcoupling of PeLED. a, Binary image of the morphology of a perovskite film obtained from SEM micrographs. **b**, top view of the perovskite emissive layer (central region) in the 3D-FDTD model of the PeLED. The disordered patterns are directly imported from the SEM image and modeled as isolated islands with a height of 50 nm. The 20 dipole positions to simulate light out-coupling processes are indicated as crossbars. **c**, Cross-sectional view of the refractive index distribution in the PeLED model. The thickness of ITO, hole-transporting layers (HTL, poly-TPD and PVK), electron-transporting layers (ETL, TPBi) above perovskite grains, and ETL above HTL were set as 150 nm, 20 nm, 30 nm, and 30 nm, respectively. The white dashed line indicates the vertical position to extract the top-view image as shown in **b**. The refractive indices at 483 nm are 1.9+0.004i (ITO), 2.3 (perovskite), 1.75 (HTL and ETL), and 0.72+5.9i (Al).

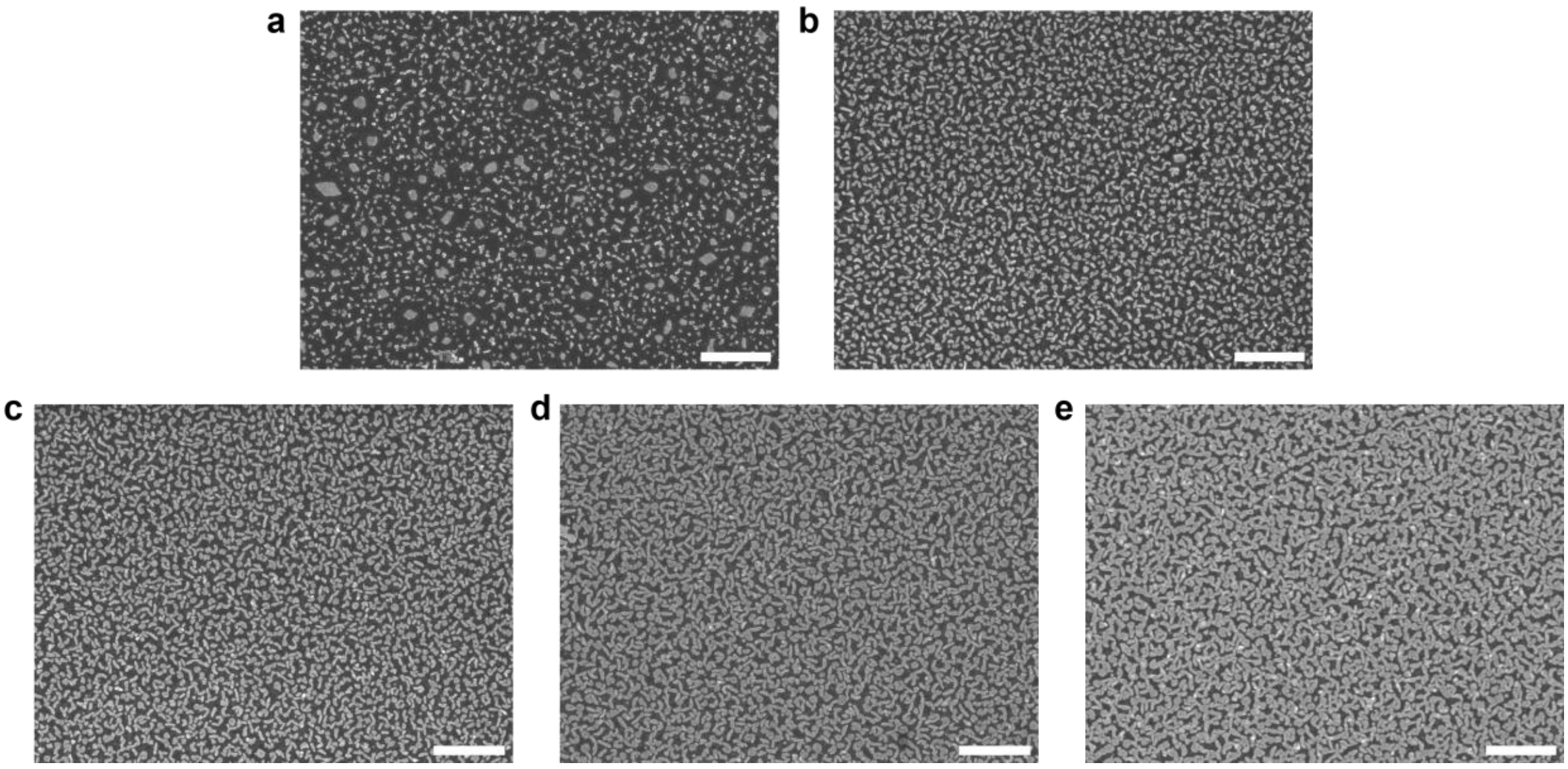

Supplementary Fig. 20 | SEM micrographs of 35%Cl perovskite with different film coverage. a, Film coverage of ~18% (0.06 M Pb). **b**, film coverage of ~34% (0.08 M Pb). **c**, film coverage of ~42% (0.10 M Pb). **d**, film coverage of ~51% (0.12 M Pb). **e**, film coverage of ~62% (0.14 M Pb). The scale bar is 3 μm. The film coverage was estimated using the software ImageJ.

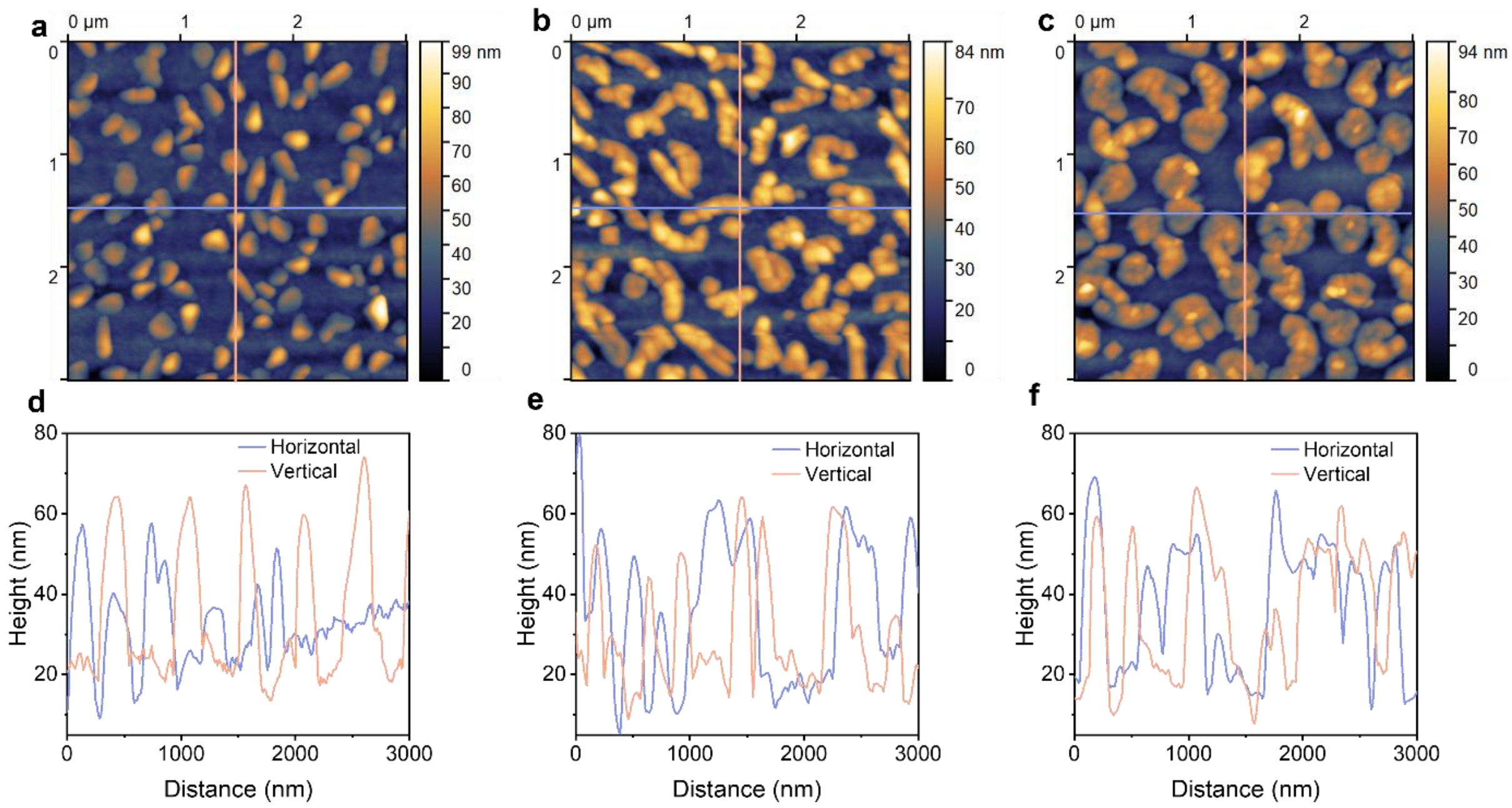

Supplementary Fig. 21 | AFM images of 35%Cl perovskite with different film coverage. **a**, 35%Cl perovskite (0.06 M Pb) on ITO/PVK after 12 minutes of treatment and its height profile in **d**. **b**, 35%Cl perovskite (0.10 M Pb) on ITO/PVK after 12 minutes of treatment and its height profile in **e**. **c**, 35%Cl perovskite (0.14 M Pb) on ITO/PVK after 12 minutes of treatment and its height profile in **f**. These results indicate that varying the concentration leads to different film coverage but a comparable grain height (~50 nm).

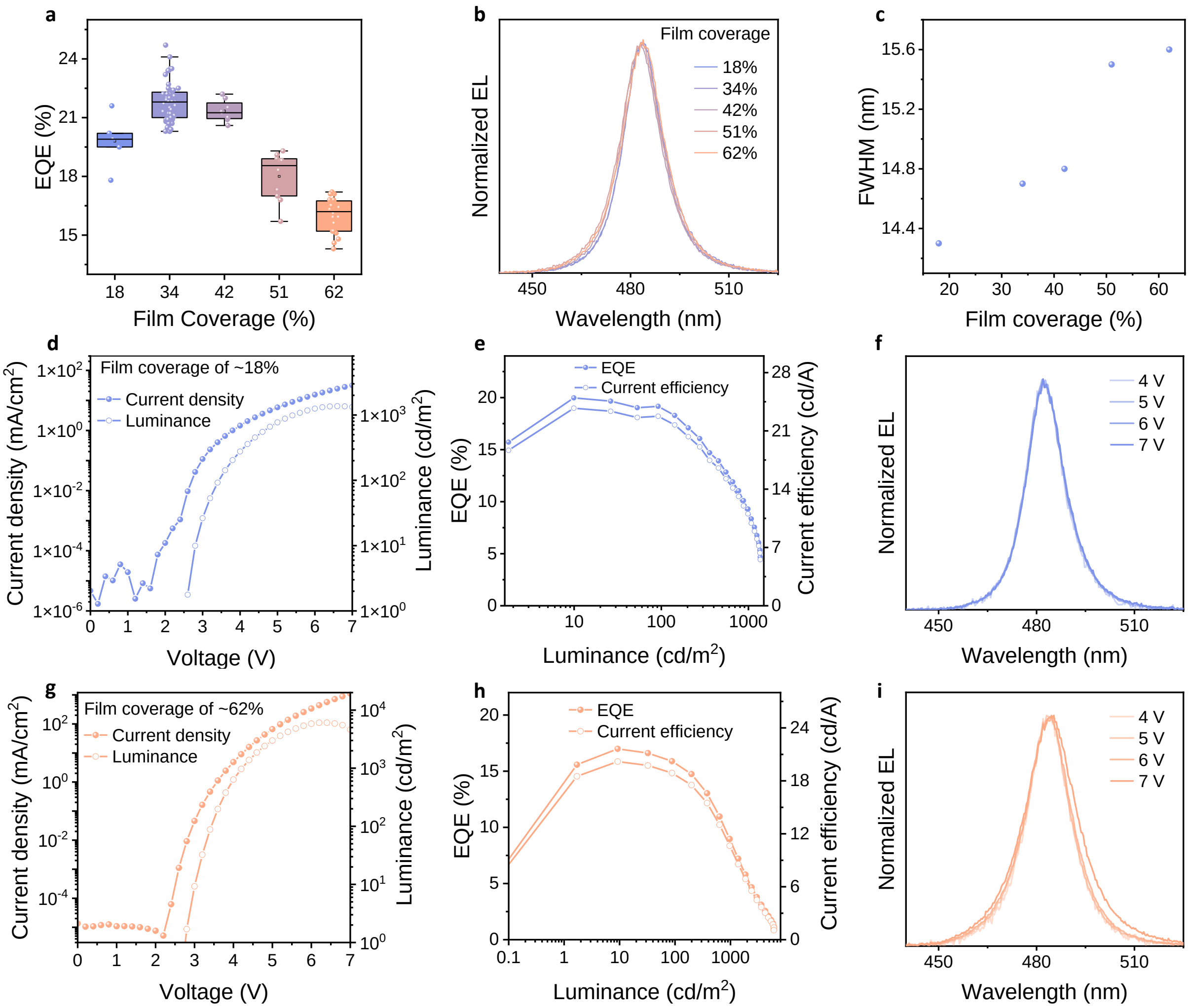

Supplementary Fig. 22 | Device performance of 35%Cl PeLEDs with different film coverage. **a**, Average EQE (**a**), EL spectra (**b**), and FWHM (**c**) of 35%Cl PeLEDs using perovskite thin films with different film coverage. The number of PeLEDs is 5, 57, 8, 10, 20 for devices with film coverage of 18%, 34%, 42%, 51%, and 62%, respectively. The EL spectra were obtained under a bias voltage of 6 V. Voltage-current density-luminance characteristics (**d**), luminance-EQE-current efficiency (**e**), and EL spectra (**f**) of 35%Cl PeLEDs with a film coverage of ~18%. Voltage-current density-luminance characteristics (**g**), luminance-EQE-current efficiency (**h**), and EL spectra (**i**) of 35%Cl PeLEDs with a film coverage of ~62%. The increased FWHM of PeLEDs with a high film coverage is consistent with its broad FWHM of perovskite thin films after DMF vapor treatment (Supplementary Fig. 29b), which should be attributed to insufficient halide interdiffusion and ion exchange in concentrated perovskite thin films. Perovskite with a film coverage of ~18% was fabricated with a concentration of ~0.06 M Pb, while that with a film coverage of ~62% was prepared with a concentration of ~0.14 M Pb.

We observed that reducing the film coverage not only improved the light outcoupling (Fig. 4b) but also contributed to higher electroluminescence color purity (Supplementary Fig. 22c) and enhanced spectral stability (Supplementary Fig. 22f). We attribute this to the greater compositional homogeneity and higher PLQY (Supplementary Fig. 30a) in the diluted films from smooth ion diffusion and crystallization assisted by DMF vapor.

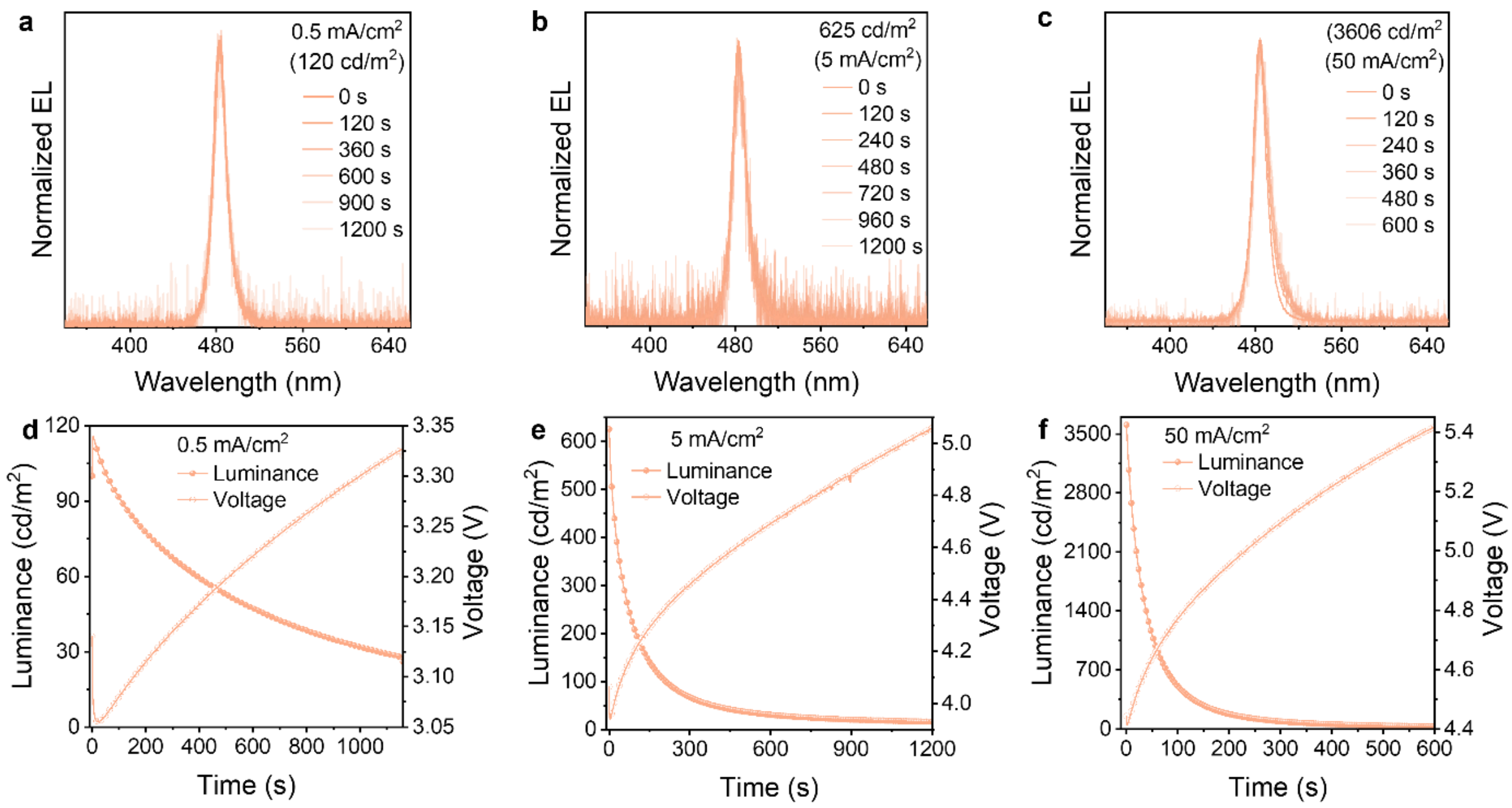

Supplementary Fig. 23 | EL spectral stability of 35%Cl PeLEDs. EL spectra of 35%Cl PeLEDs under operation at a constant current density of 0.5 mA cm^{-2} (**a**), 5 mA cm^{-2} (**b**), 50 mA cm^{-2} (**c**). Decay of the 35%Cl PeLEDs under operation at a constant current density of 0.5 mA cm^{-2} (**d**), 5 mA cm^{-2} (**e**), 50 mA cm^{-2} (**f**). The stability was conducted in the air after device encapsulation.

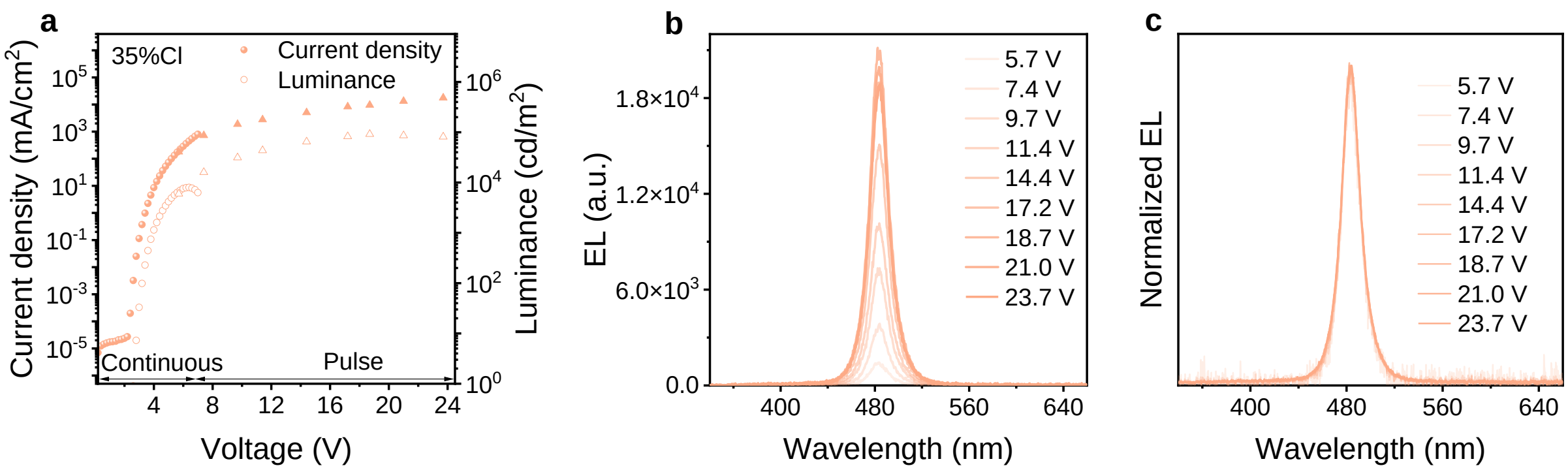

Supplementary Fig. 24 | Pulse operation of 35%Cl PeLEDs. **a**, Voltage-current density-luminance characteristics of 35%Cl PeLEDs under continuous and pulsed mode. **b**, the corresponding EL spectra of 35%Cl PeLEDs under pulsed mode. **c**, Normalized EL spectra of 35%Cl PeLEDs under pulsed mode. The pulse width is 500 ns with a frequency of 10 Hz.

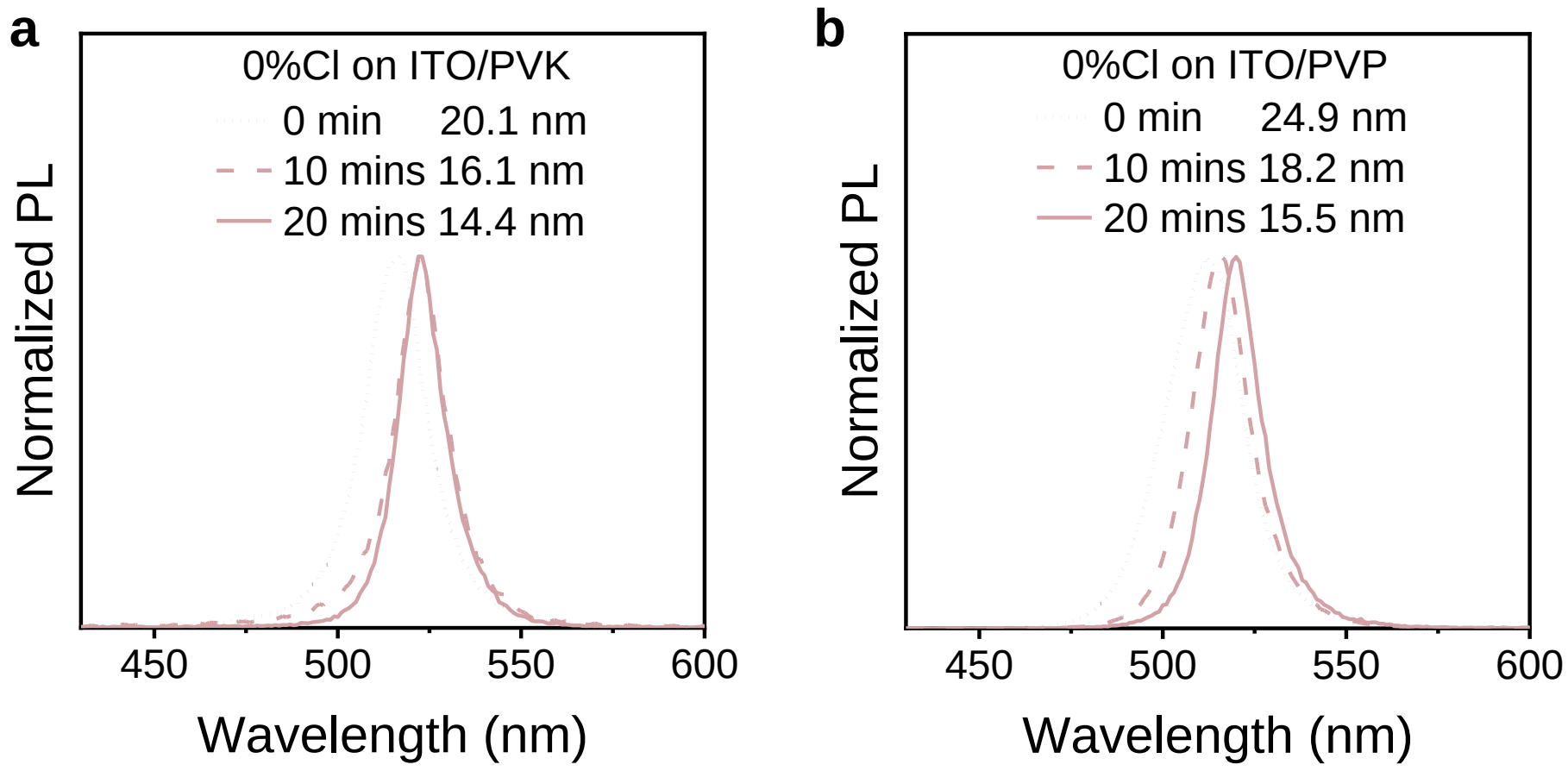

Supplementary Fig. 25 | PL spectra of 0%Cl perovskite after post-treatment. **a**, PL spectra of 0%Cl perovskite on ITO/PVK after DMF vapor treatment. **b**, PL spectra of 0%Cl perovskite on ITO/PVP after DMF vapor treatment. The redshift of PL spectra after DMF vapor treatment should be due to the incorporation of FA into the perovskite, which results in reduced bandgap. The FWHM of post-treated 0%Cl perovskite is comparable on ITO/PVK and ITO/PVP. This is reasonable because no Cl-rich perovskite (no Cl in the precursor) can be generated.

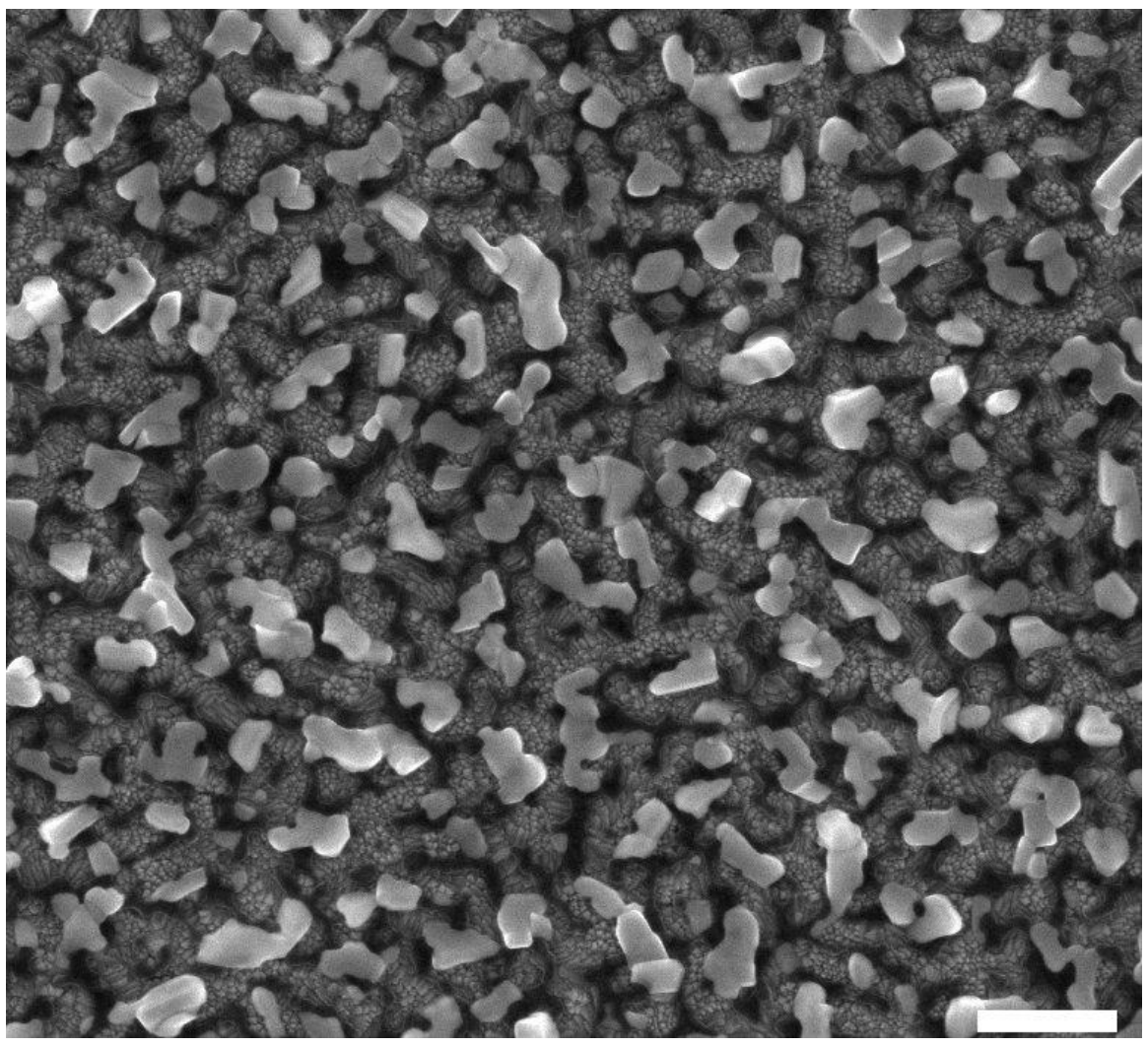

Supplementary Fig. 26 | SEM image of vapor-treated 0%Cl perovskite on ITO/PVK. The scale bar is 500 nm.

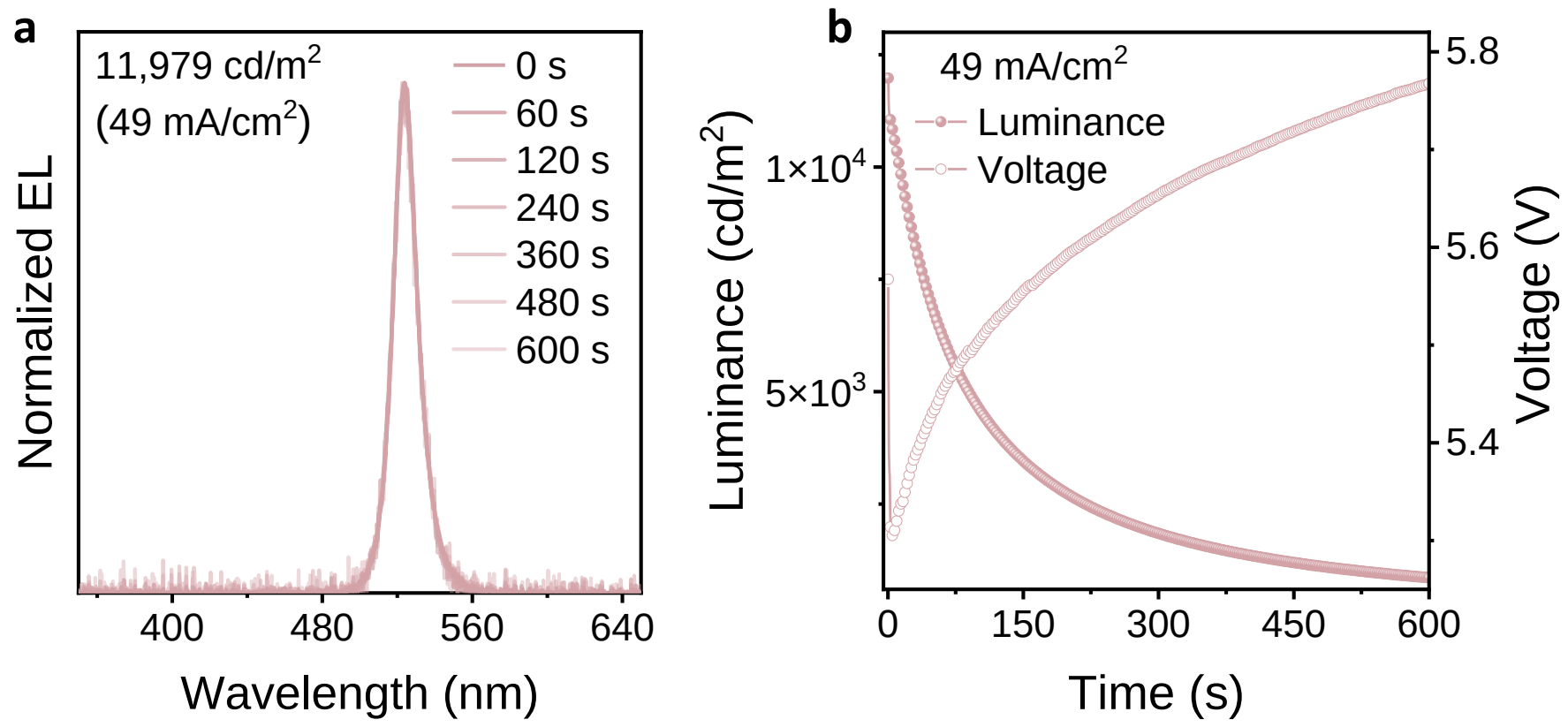

Supplementary Fig. 27 | EL spectral stability of 0%Cl PeLEDs. **a**, EL spectra of 0%Cl PeLEDs under a constant current density of 49 mA cm^{-2} and their luminance decay (**b**).

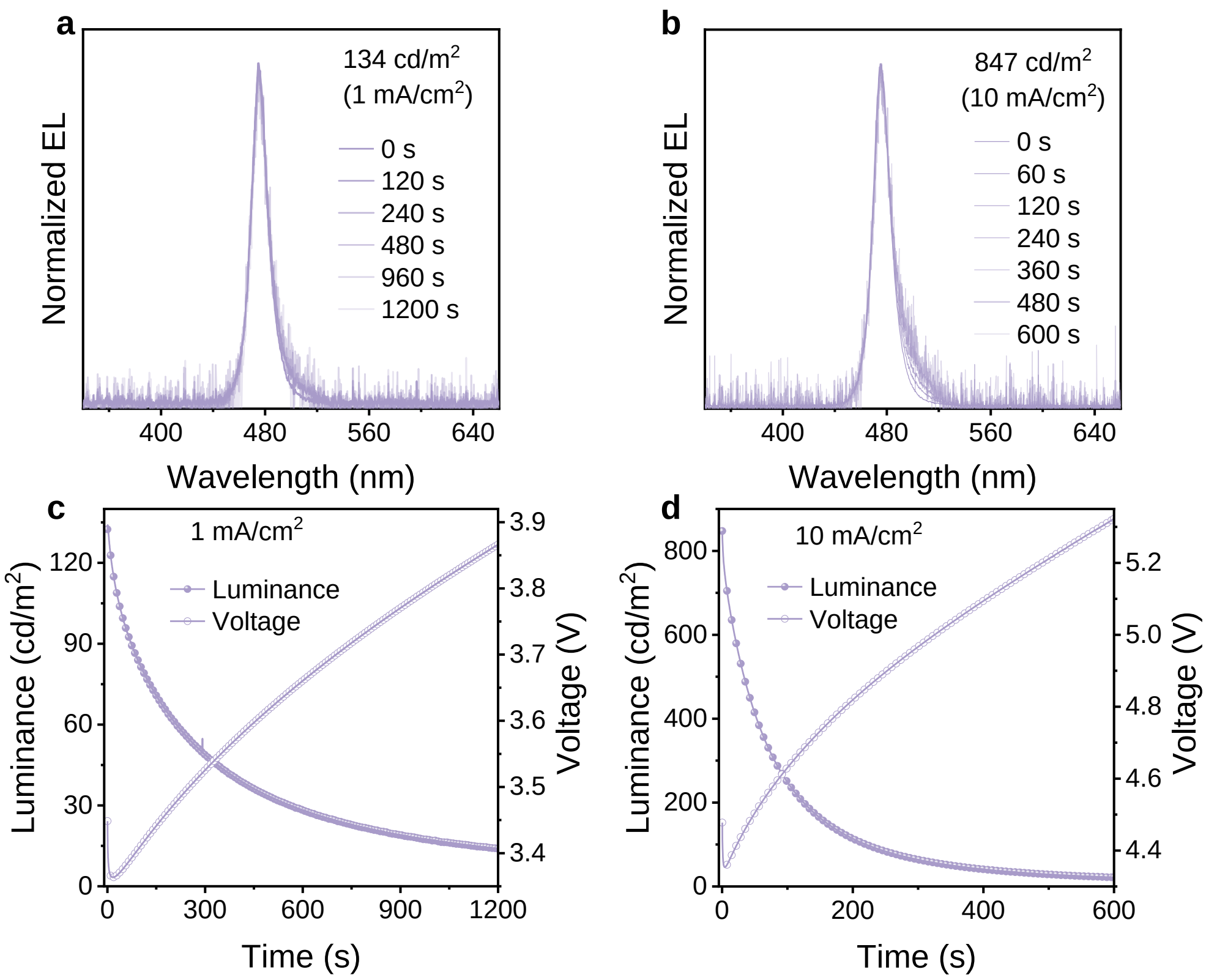

Supplementary Fig. 28 | EL spectral stability of 40%Cl PeLEDs. **a**, EL spectra of 40%Cl PeLEDs under a constant current density of 1 mA cm^{-2} (initial luminance of 134 cd m^{-2}) and their luminance decay (**c**). **b**, EL spectra of 40%Cl PeLEDs under a constant current density of 10 mA cm^{-2} (initial luminance of 847 cd m^{-2}) and their luminance decay (**d**).

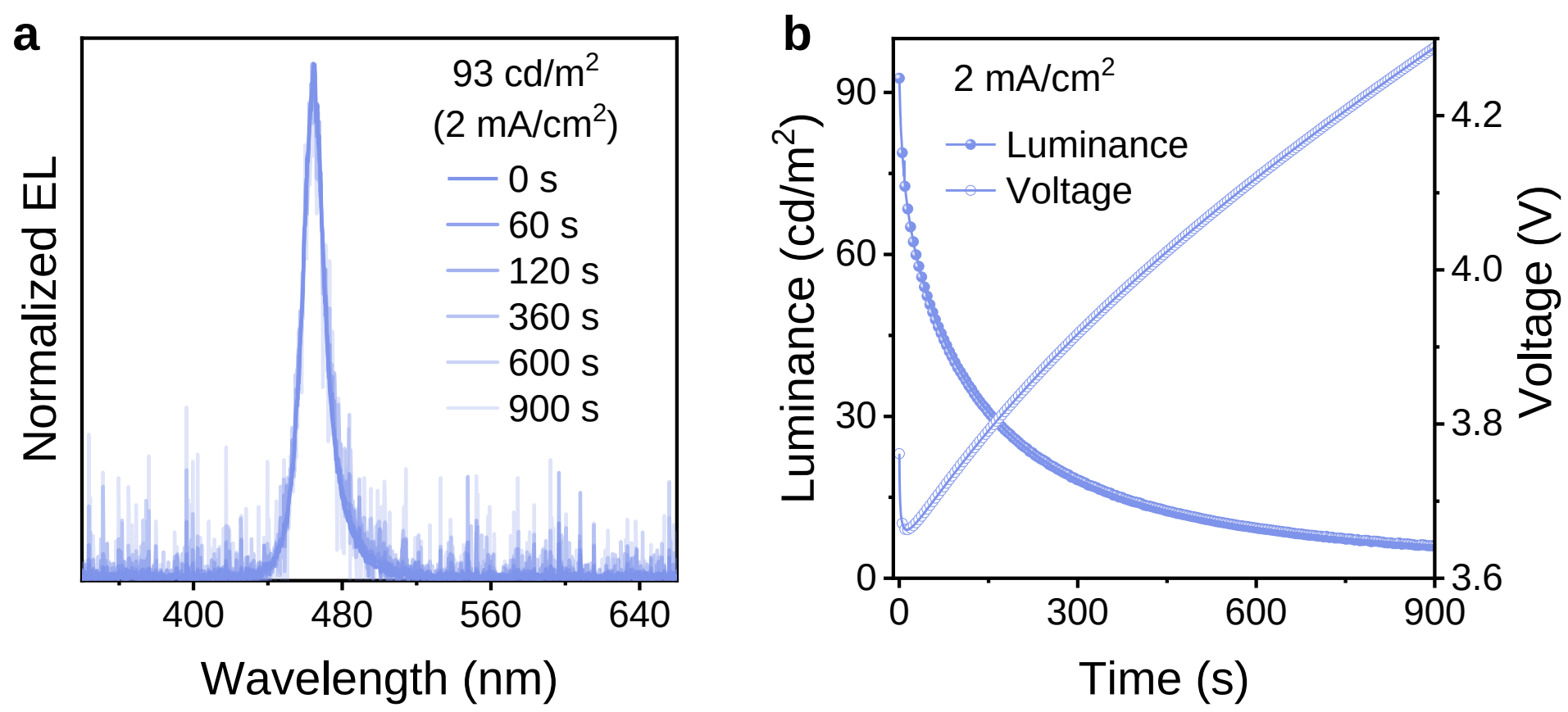

Supplementary Fig. 29 | EL spectral stability of 48%Cl PeLEDs. **a**, EL spectra of 48%Cl PeLEDs under a constant current density of 2 mA cm^{-2} and their luminance decay (**b**).

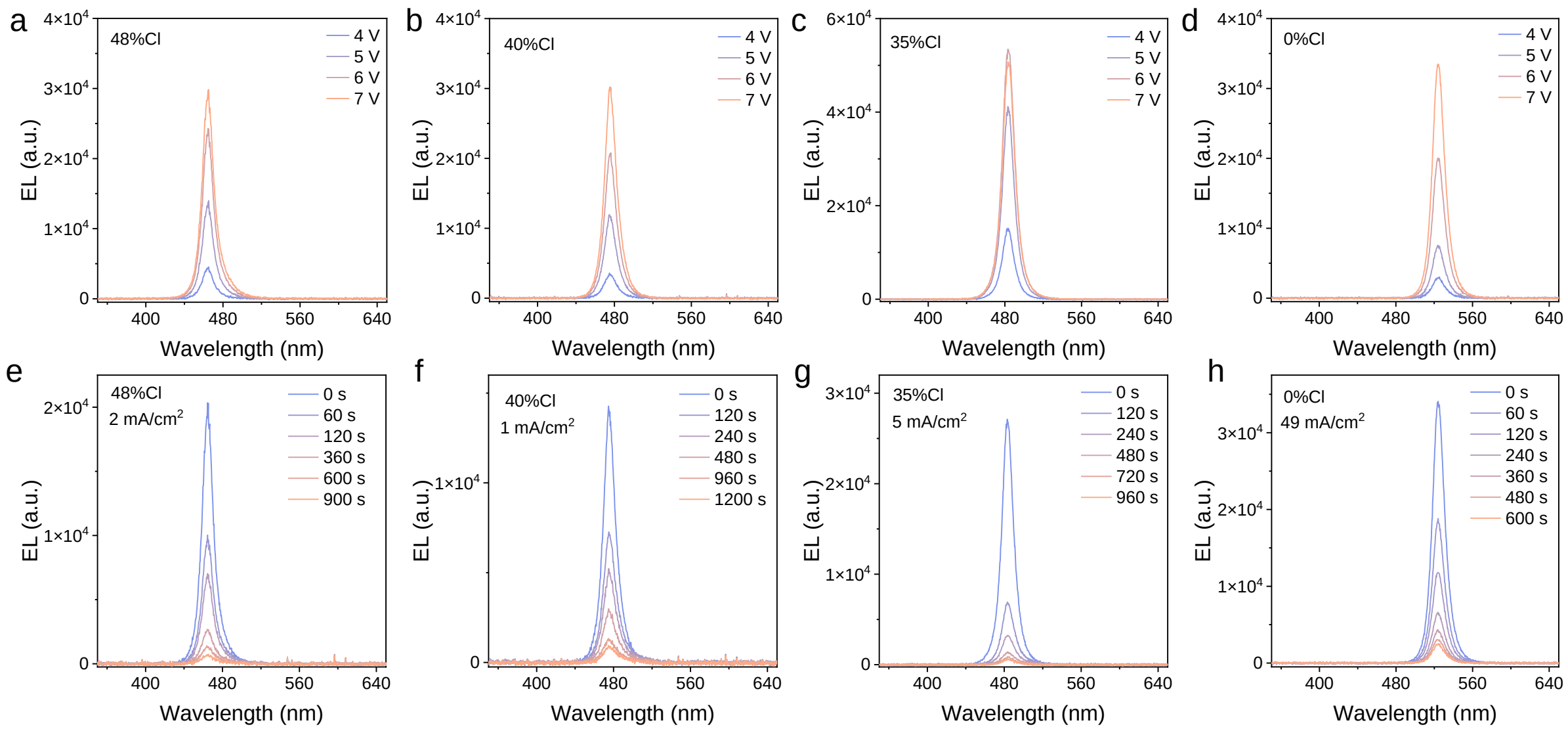

Supplementary Fig. 30 | EL spectra of 48%Cl, 40%Cl, 35%Cl, and 0%Cl PeLEDs. Voltage-dependent EL spectra of 48%Cl PeLEDs (**a**), 40%Cl PeLEDs (**b**), 35%Cl PeLEDs (**c**), PeLEDs 0%Cl (**d**). EL spectra of 48%Cl PeLEDs (**e**), 40%Cl PeLEDs (**f**), 35%Cl PeLEDs (**g**), PeLEDs 0%Cl (**h**) under continuous constant current density excitation.

The EL spectra were recorded using an Ocean Optics Flame spectrometer without any smoothing. To avoid EL signal saturation (maximum counts of ~6.28×10^4) at high luminance under a bias of 6-7 V, we employed a short integration time of approximately 50 ms during device characterization. While this setting is necessary to ensure accurate spectral acquisition at high brightness, it also reduces the signal-to-noise ratio at low luminance under biases of 3-4 V. As a result, the EL spectra collected at lower driving voltage appear slightly noisy.

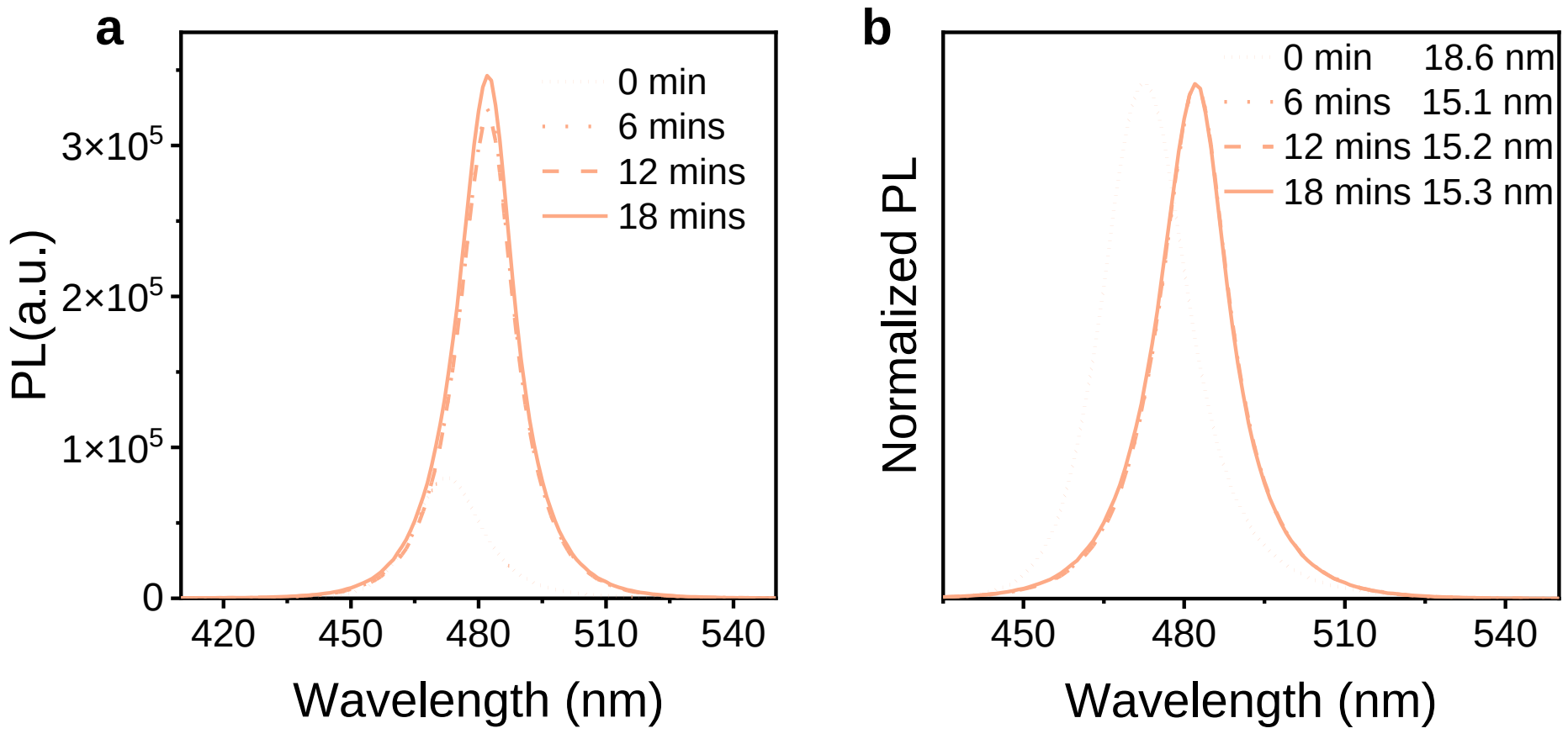

Supplementary Fig. 31 | PL spectra of 35%Cl perovskite with a film coverage of ~61%. **a**, PL spectra of 35%Cl perovskite on ITO/PVK after DMF vapor treatment. **b**, Normalized PL spectra of 35%Cl perovskite on ITO/PVK after DMF vapor treatment. The concentration of perovskite precursor is 0.14 M Pb.

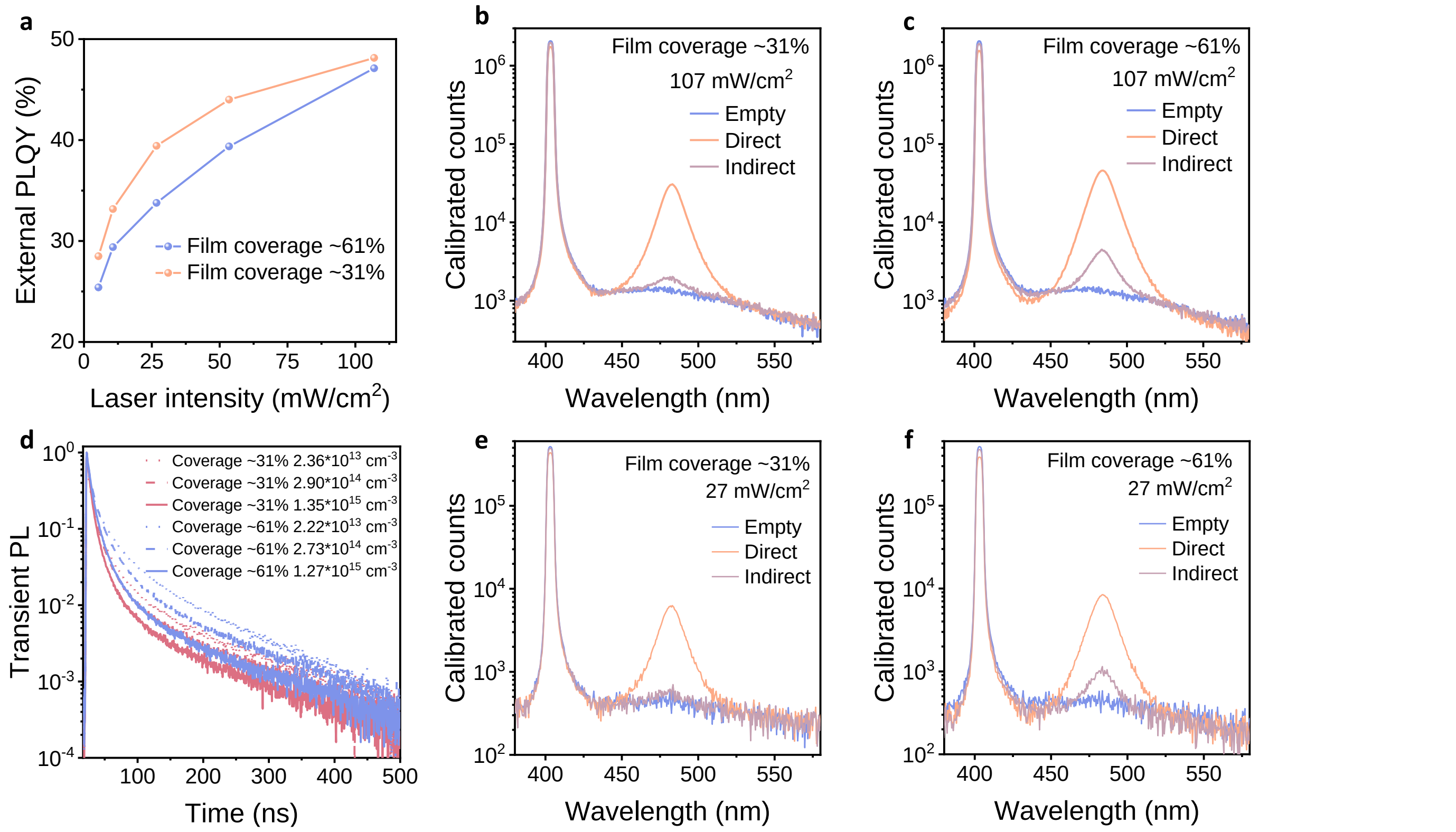

Supplementary Fig. 32 | External PLQY of 35%Cl perovskite with different film coverage. **a**, External PLQY of 35%Cl on ITO/poly-TPD/PVK with a film coverage of ~31% and ~61%. PL spectra of perovskite with a film coverage of ~31% under the illumination of laser intensity of ~107 mW cm^{-2} (**b**) and ~27 mW cm^{-2} (**e**). Empty indicates that no perovskite thin film was placed in the integrating sphere. Direct indicates that the perovskite thin film was directly illuminated by the laser in the integrating sphere. Indirect indicates that the perovskite thin film was indirectly illuminated (the laser was scattered by the integrating sphere) by the laser in the integrating sphere. PL spectra of perovskite with a film coverage of ~61% under the illumination of laser intensity of ~107 mW cm^{-2} (**c**) and ~27 mW cm^{-2} (**f**). **d**, Carrier density-dependent transient PL of perovskite with film coverage of ~61% and ~31%. Note that 1.5 mg/ml PVP was spin-coated on PVK but it was eventually dissolved in the perovskite precursors.

The carrier density[6] is calculated according to $n = \frac{1}{\pi r^2 d \Lambda}\frac{\alpha P}{h\nu f}$ with πr^2 the area of the laser spot (2.12 $\times 10^{-3}$ cm^2), d the thickness of perovskite thin films (45 nm), Λ the film coverage, α the absorptance (15.3% and 8.3% for perovskite thin films with film coverage of ~61% and ~31%, respectively), P the laser power (47.4 µW, 10.2 µW, and 0.83 µW, corresponding to a fluence of 11.2 nJ pulse^{-1} cm^{-2}, 2.4 nJ pulse^{-1} cm^{-2}, and 0.19 nJ pulse^{-1} cm^{-2}), f the laser repetition rate (2 MHz) and $h\nu$ the photon energy (405 nm, equivalent to 3.06 eV and 4.90 $\times$ 10^{-19} J) of the laser.

The initial fast decay phase in Supplementary Fig. 32d is attributed to band-to-band radiative recombination, while the subsequent slower decay phase is associated with first-order trap-assisted recombination. Notably, the fast decay phase accelerates as the excitation fluence increases in both films, with the diluted films exhibiting faster initial decay compared to the concentrated films. Additionally, the slow decay phase is faster in the concentrated sample than in the diluted sample, indicating the presence of more defect states in the concentrated sample, which is consistent with the higher PLQY of the diluted films (Supplementary Fig. 32a).

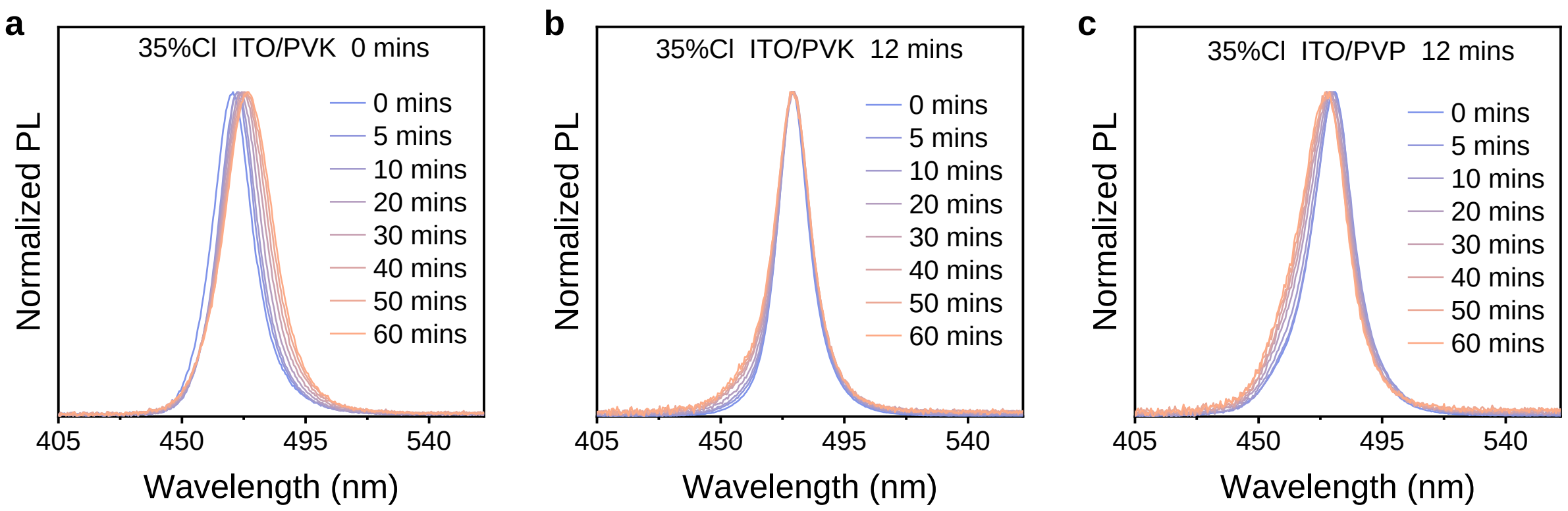

Supplementary Fig. 33 | PL spectral stability of 35%Cl perovskite thin films. **a**, PL spectra of 35%Cl perovskite on ITO/PVK without DMF vapor treatment. **b**, PL spectra of 35%Cl perovskite on ITO/PVK after 12 minutes of treatment. **c**, PL spectra of 35%Cl perovskite on ITO/PVP after 12 minutes of treatment. The PL spectra were obtained after continuous illumination of the perovskite thin films using a CW 405 nm laser with an excitation fluence of 1.5 W/cm^2. The concentration of the perovskite was 0.08M, which gave a film coverage of ~31%.

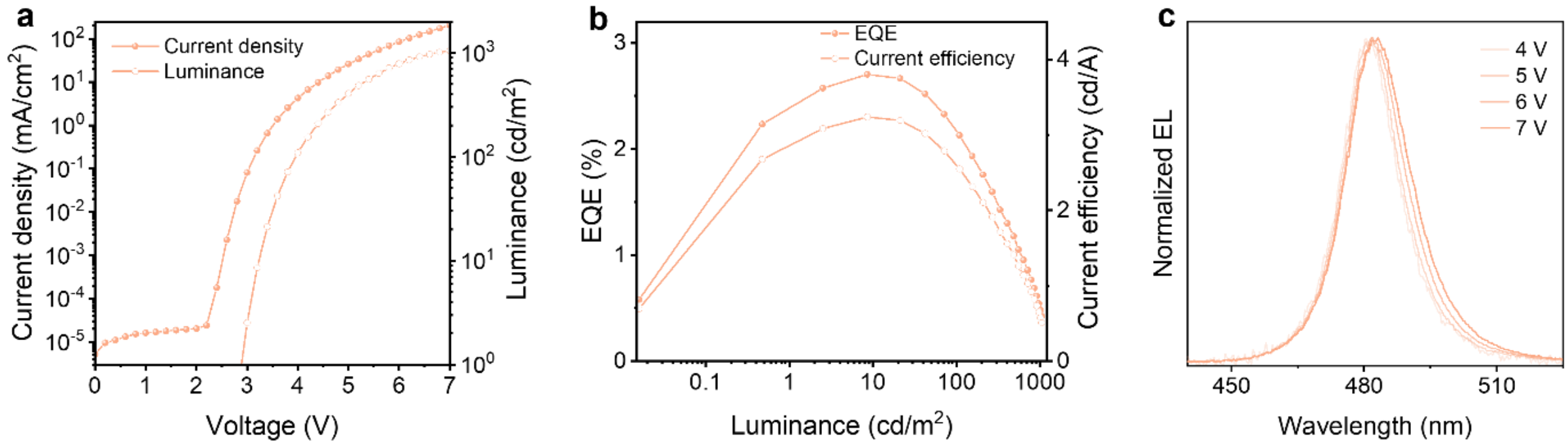

Supplementary Fig. 34 | Device performance of 35%Cl PeLEDs without vapor exposure. **a**, Voltage-current density-luminance characteristics of the device. **b**, Luminance-EQE-current efficiency of the device. **c**, voltage-dependent EL spectra of the device. The device structure is the same as 35%Cl PeLEDs after vapor exposure with 1.5 mg/ml PVP atop PVK.

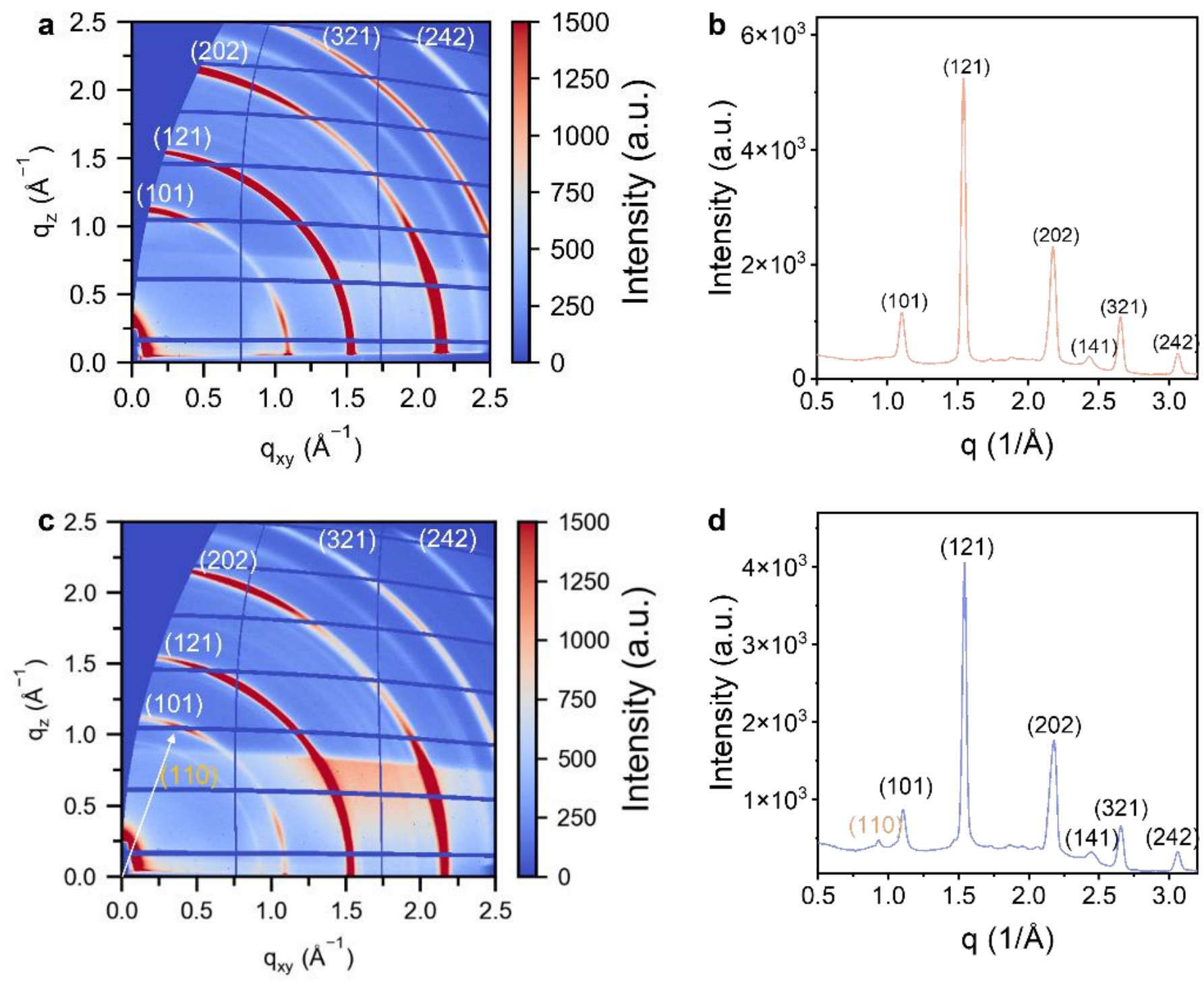

Supplementary Fig. 35 | GIWAXS pattern of 35%Cl perovskite films with different film coverage. GIWAXS pattern (**a**) of 35%Cl perovskite films on ITO/poly-TPD (~5 nm)/PVK (~15 nm) with film coverage of ~31% and its 2D plot (**b**) by integrating the intensity between 3° and 90°. GIWAXS pattern (**c**) of 35%Cl perovskite films on ITO/poly-TPD (~5 nm)/PVK (~15 nm) with film coverage of ~61% and its 2D plot (**d**) by integrating the intensity between 3° and 90°. Note that 1.5 mg/ml PVP was spin-coated on PVK but it was eventually dissolved in the perovskite precursors. The perovskite thin films were after DMF vapor exposure for 6 minutes. The yellow (110) plane in (**d**) was assigned to non-perovskite phase Cs_4PbBr_6, which likely originates from local Cs enrichment or Pb deficiency, both of which can occur under concentrated perovskite precursor (film coverage of ~61%) due to inefficient ion redistribution during DMF vapor-assisted crystallization. The presence of Cs_4PbBr_6 can disturb the local stoichiometric ratio in the mixed-halide perovskite, leading to compositional inhomogeneity and broad FWHM. In addition, we observed that the (101) plane was tilted ~20° with respect to the substrate, probably due to obstructed growth of perovskite by neighboring grains because the grains are densely packed in concentrated perovskite films (Supplementary Fig. 20e and 21c).

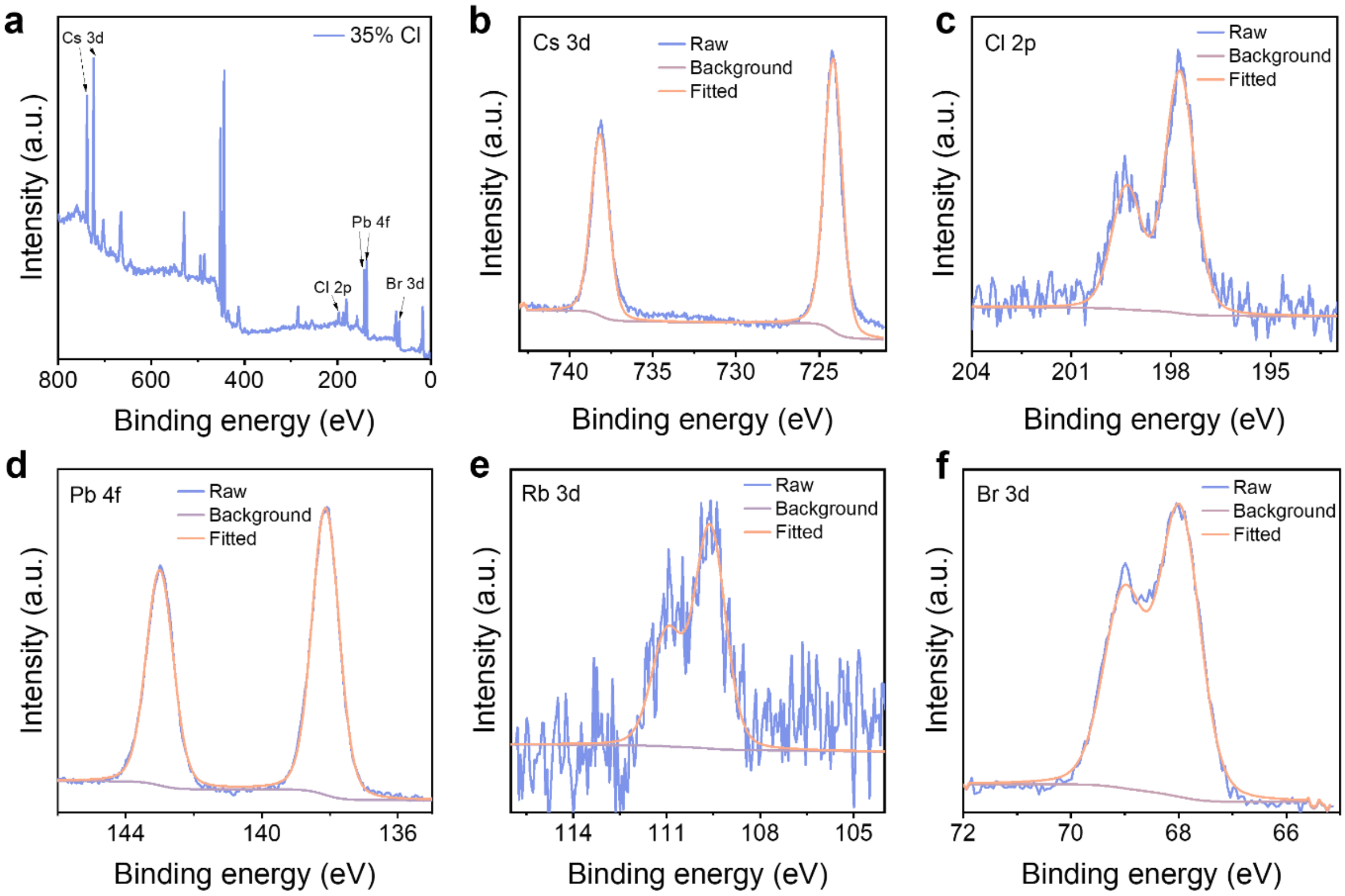

Supplementary Fig. 36 | XPS analysis of 35%Cl perovskite on ITO after vapor exposure for 12 minutes. a, Full survey scan of the perovskite films. **b-f**, High-resolution spectra with peak fitting for Cs (**b**), Cl (**c**), Pb (**d**), Rb (**e**), and Br (**f**). The estimated elemental ratios are Cl/(Br+Cl) = 43.2% and Pb/(Br+Cl) = 28.3%.

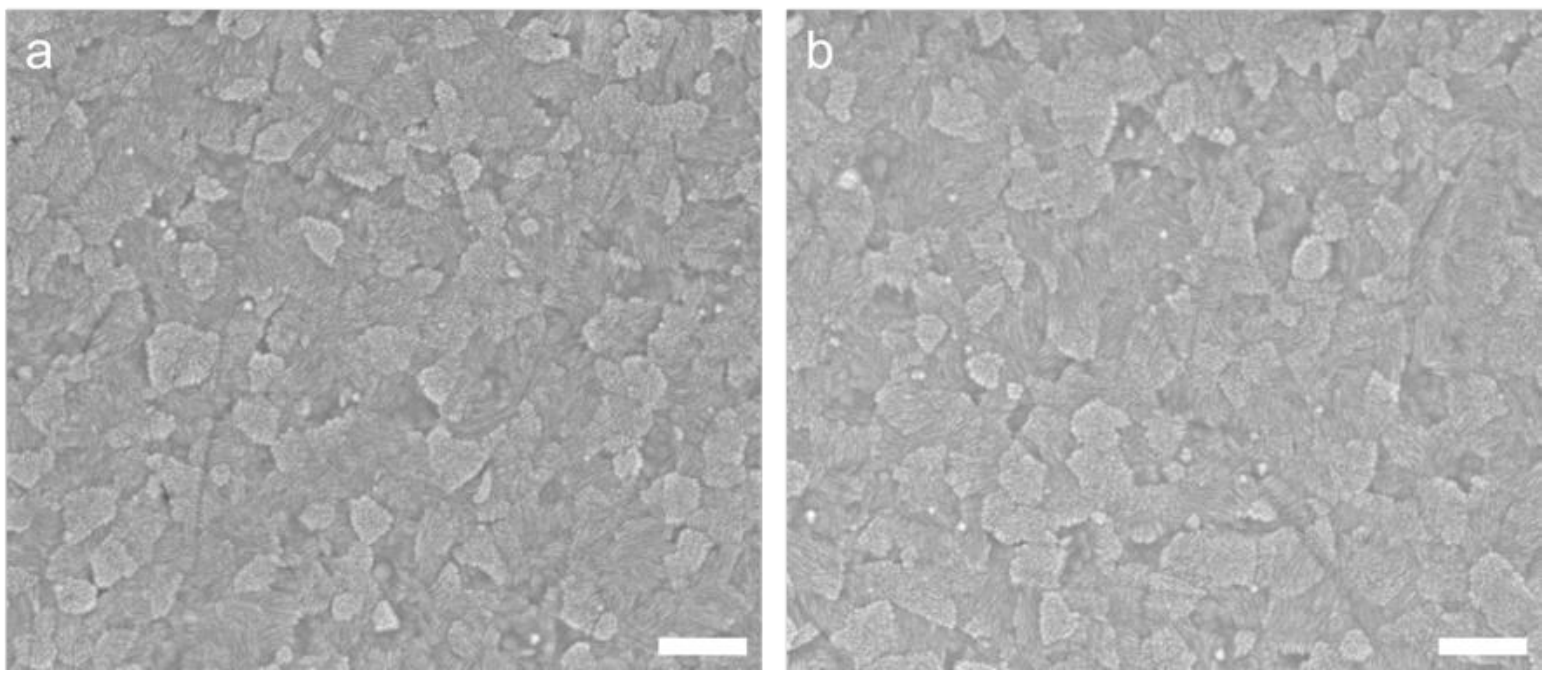

Supplementary Fig. 37 | SEM images of ITO/PVK before and after DMF vapor treatment. **a**, before DMF vapor treatment. **b**, after DMF vapor treatment for 6 minutes. The scale bars are 500 nm.

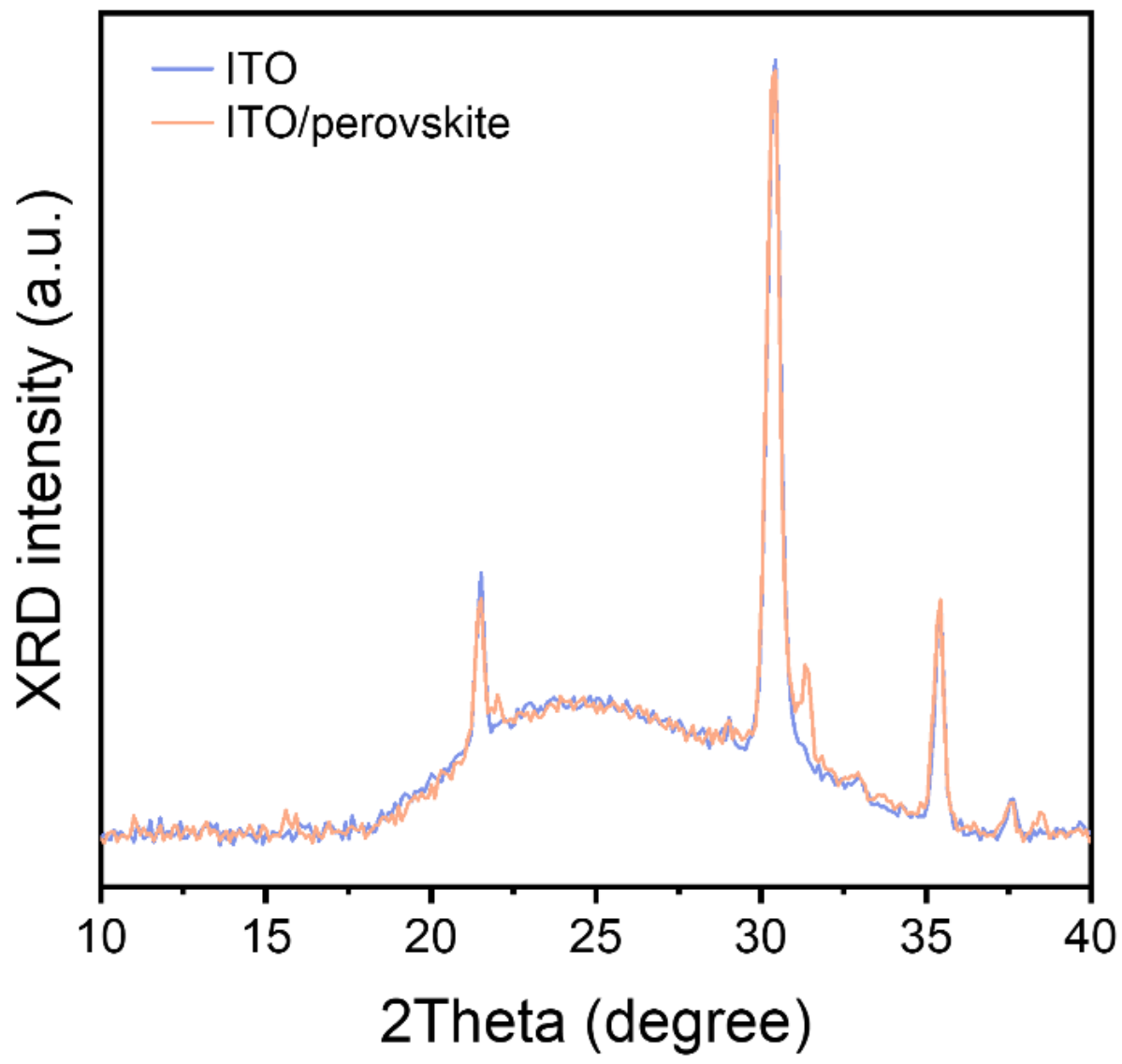

Supplementary Fig. 38 | XRD of 35%Cl perovskite on ITO after vapor exposure for 12 minutes.

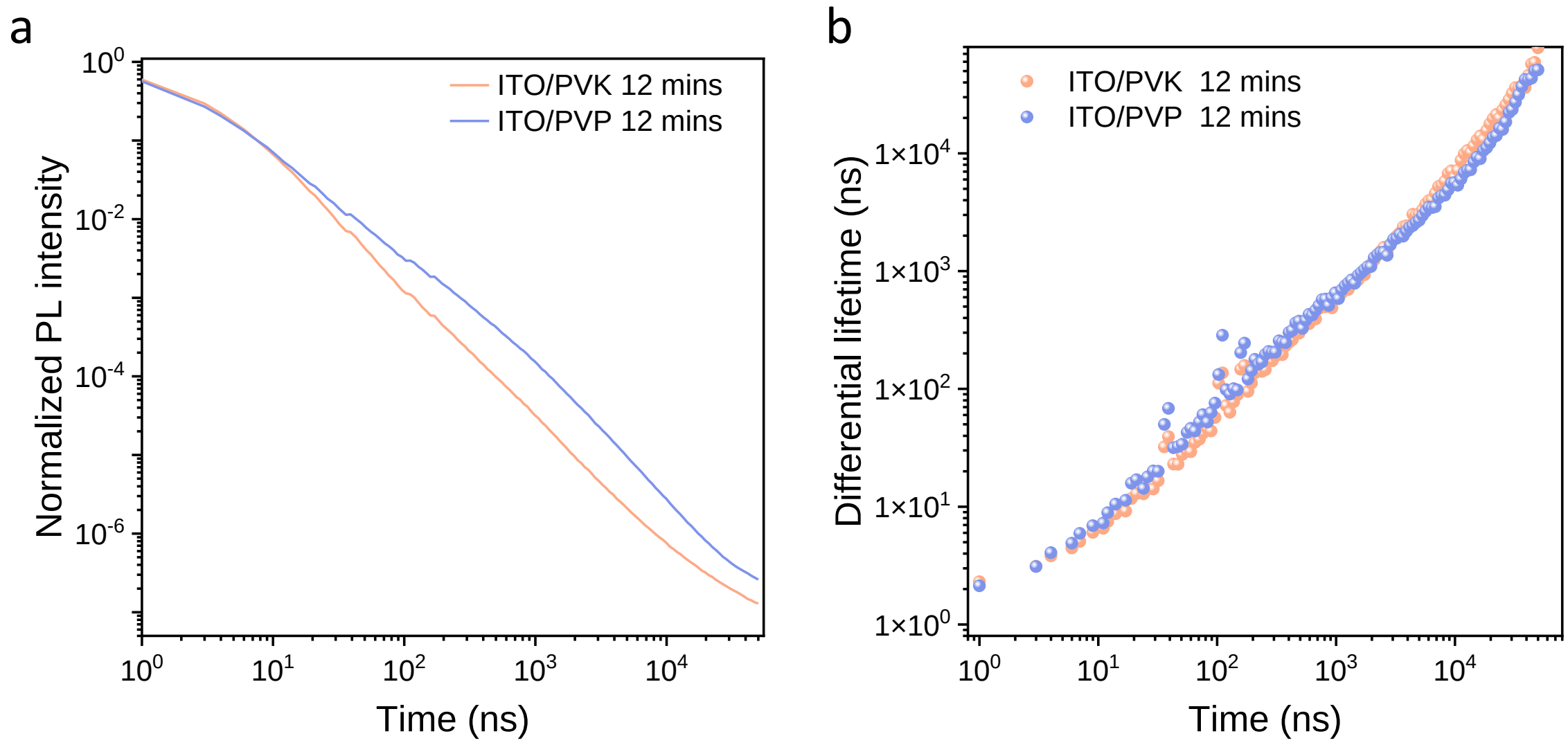

Supplementary Fig. 39 | Time-resolved PL (TRPL) of vapor-treated perovskite on ITO/PVK and ITO/PVP. **a**. Transient PL of 35%Cl perovskite on ITO/PVK and ITO/PVP after DMF vapor treatment for 12 minutes. **b**. Differential lifetime of 35%Cl perovskite on ITO/PVK and ITO/PVP after DMF vapor treatment for 12 minutes. The TRPL was conducted by using a 400 nm pulsed laser with a fluence of 1.53 μJ cm^{-2} $pulse^{-1}$ and a repetition rate of 500 kHz. The absorptance of perovskite thin films was approximately 8.3% and the thickness was ~45 nm. Therefore, the estimated initial carrier density was approximately 5.68×10^{16} cm^{-3}. The differential lifetime was estimated using the equation $\tau_{diff}(t) = -(\frac{dln(PL(t))}{dt})^{-1}$ following ref.7.

As shown in Supplementary Fig. 39b, the two perovskite films exhibit similar differential lifetime over the delay range. In addition, no clear plateau in the differential lifetime is observed for either film, which prevents a reliable extraction of the trap-assisted Shockley-Read-Hall (SRH) lifetime. Given the complexity of the possible recombination processes in these island-like films, which may also include both excitons and free charges, a rigorous determination of decay metrics is beyond the scope of this current work.

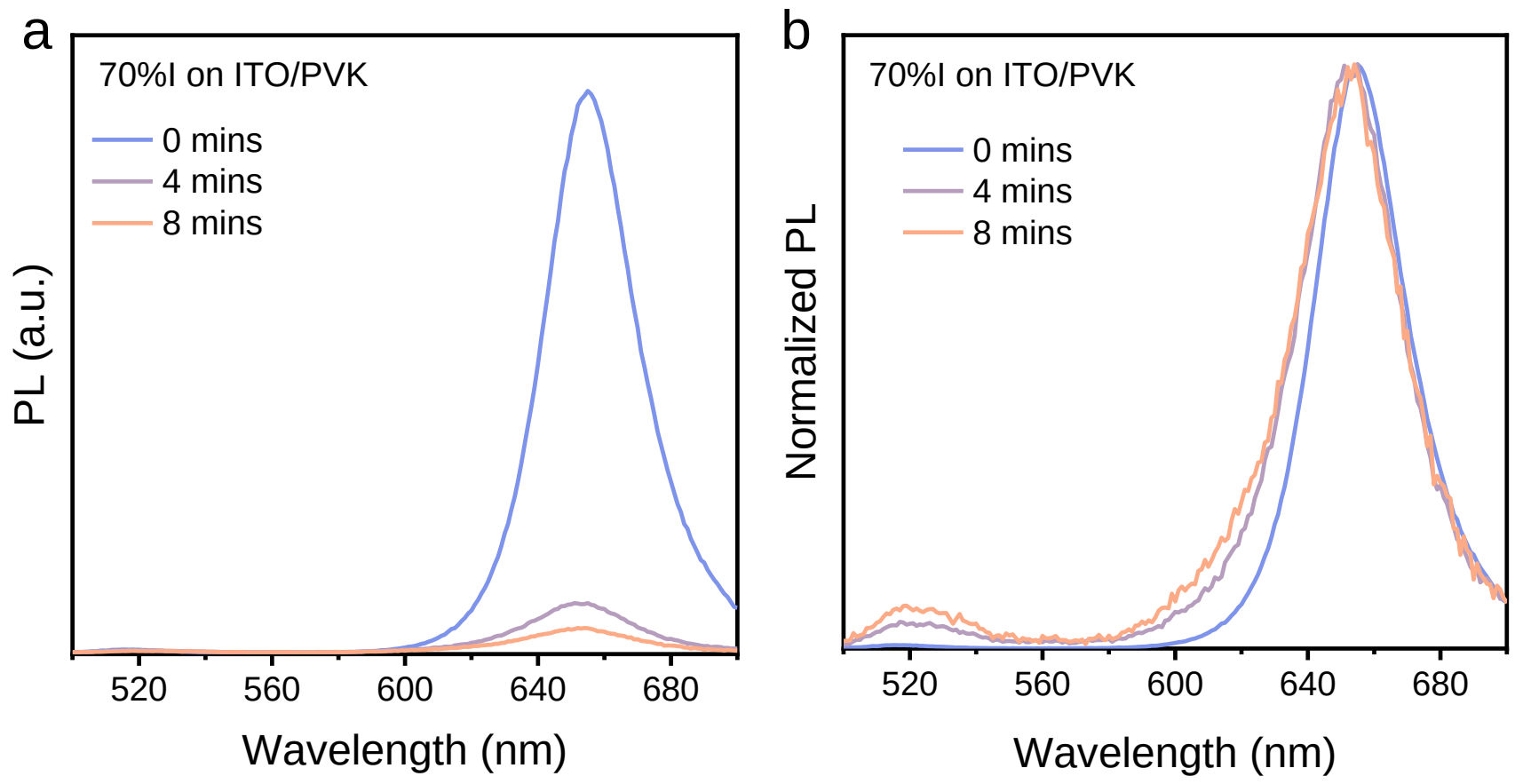

Supplementary Fig. 40 | PL spectra of 70%I (30%Br) perovskite on ITO/PVK after DMF vapor treatment.

The PL intensity of 70%I (30%Br) perovskite thin films decreases remarkably after DMF vapor treatment. Meanwhile, the PL spectrum becomes broader and an additional peak appears at approximately 520 nm, suggesting increased phase segregation after vapor treatment. These results suggest that direct application of the present interface-regulated vapor crystallization strategy to the mixed Br/I perovskite system is not yet straightforward. This difference likely originates from the intrinsically greater tendency of mixed Br/I perovskite toward halide segregation together with their distinct crystallization kinetics. Therefore, extending this strategy to red-emitting perovskite will likely require further optimization, such as rational design of the underlayer, selection of alternative solvent vapor atmosphere, adjustment of precursor stoichiometry, or even modulation of the treatment temperature and pressure in order to realize highly color-pure red emission.

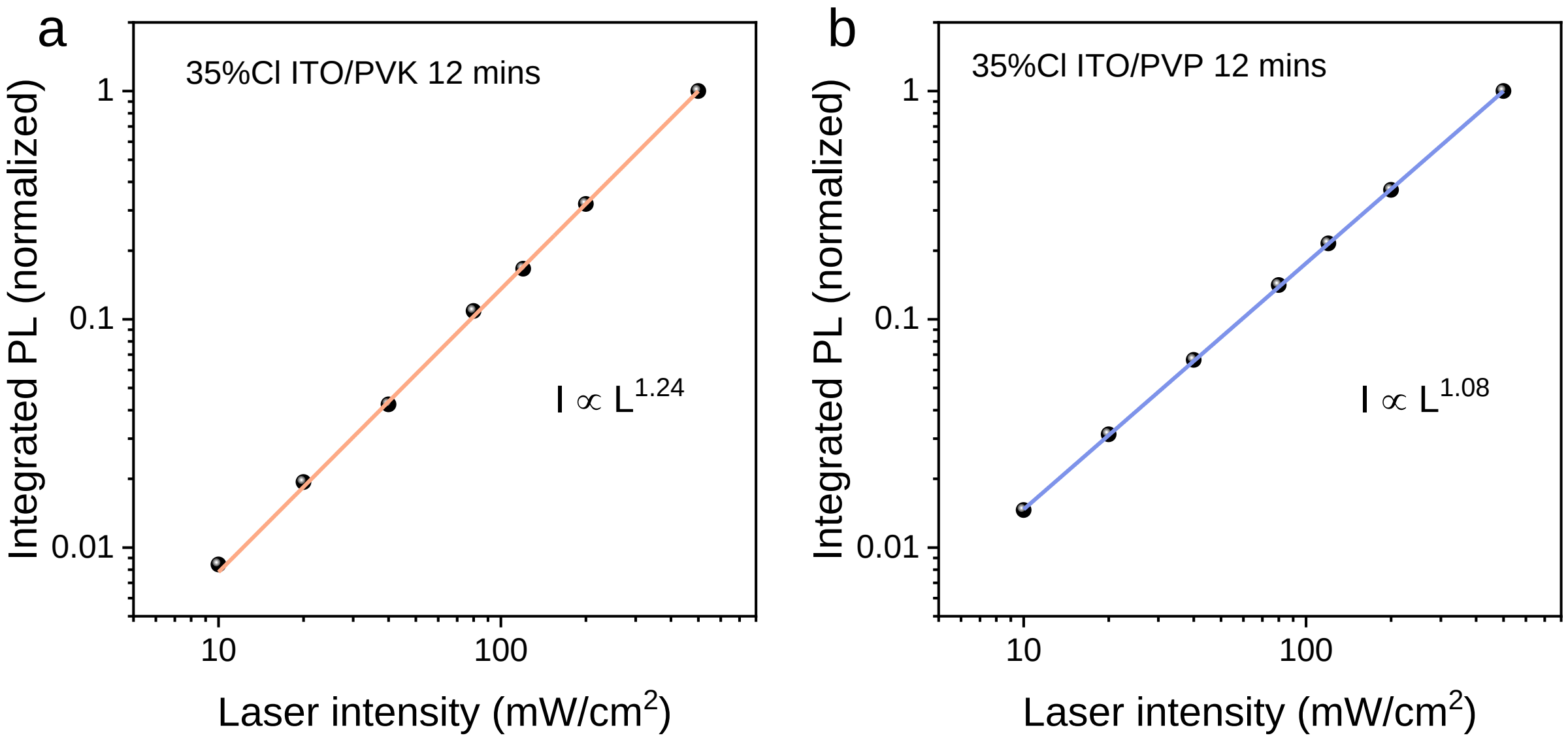

Supplementary Fig. 41 | Logarithm plot of the integrated PL intensity versus laser intensity. Logarithm plot of the integrated PL intensity (normalized) of post-treated 35%Cl perovskite on ITO/PVK (**a**) and ITO/PVP (**b**) versus laser intensity (405 nm, continuous wave).

Supplementary Note 1: Morphology change of perovskite on different underlying materials upon vapor treatment.

SEM images in Supplementary Fig. 2a revealed that, without vapor treatment, the perovskite films on ITO/PVK consisted of small grains with sizes on the order of tens of nanometers. After 1 minute of vapor exposure, substantial nanorod structures with several hundred nanometers in length were present and spatially separated across the film. Further treatment up to 6 minutes led to increased nanorod dimensions, although minimal size change was observed between 6 and 12 minutes, which is consistent with the minimal change in FWHM between 6 and 12 minutes observed in Fig.1a. In contrast, the perovskite films on ITO/PVP (Supplementary Fig. 2b) initially formed a continuous morphology without vapor treatment, but after 1 minute of vapor treatment, only a handful of large grains were observed, suggesting a slower crystallization rate as compared to the perovskite on ITO/PVK. Prolonged vapor treatment (6 minutes) resulted in flower-like clusters composed of numerous small grains, a morphology that persisted after 12 minutes of treatment. A similar clustering phenomenon was observed in post-treated 48%Cl perovskite on ITO/PVP (Supplementary Fig. 3b), indicating an underlying material-dependent morphological response to DMF vapor treatment.

Supplementary Note 2: Discussion of the excitonic absorption peak of post-treated perovskite

We observed that the excitonic absorption peak is more pronounced for post-treated perovskite on ITO/PVK than that on ITO/PVP, as shown in Extended Data Fig. 3. To better understand the carrier recombination in our perovskite thin films, we performed additional excitation power-dependent PL measurements (Supplementary Fig. 41). The integrated PL intensity (I) was fitted using a power law relationship of the form $I \propto L^k$, where L is the excitation power and k provides insight into the dominant recombination pathway. In general, a k value close to 2 has been attributed to bimolecular free-carrier recombination[8], whereas a k value close to 1 has been suggested for monomolecular excitonic recombination or photo-doping[9]. The extracted value is 1.24 and 1.08 for perovskite on ITO/PVK and ITO/PVP, respectively, suggesting a similar carrier recombination mechanism where excitonic recombination or photo-doping is playing a key role in the radiative pathway in both films, although a contribution from free-carrier recombination cannot be excluded. Therefore, we believe that the less pronounced excitonic absorption peak in post-treated perovskite on ITO/PVP than that on ITO/PVK could be due to inhomogeneity-induced obscuration of the excitonic feature.

Supplementary Note 3. Perovskite light-emitting diodes (PeLEDs) design and optimization

Although the HAADF image in Extended Data Fig. 5 suggests direct contact between the TPBi and PVK layers, the devices exhibited negligible leakage current. We attribute this behavior primarily to the large carrier injection barriers (approximately 0.5-0.6 eV) at the PVK/TPBi interface[3,10], which makes direct charge transport energetically unfavorable. This interpretation is supported by the observation that the current density of the device with the structure ITO/poly-TPD/PVK/TPBi/LiF/Al (without perovskite) was several orders of magnitude lower than that of the corresponding PeLEDs under the same bias voltage, as shown in Supplementary Fig. 15. In addition, a thin layer of poly-TPD (hole mobility of 1×10^{-4} cm^2 V^{-1} s^{-1})[11] is introduced between ITO and PVK to enhance the hole transporting capability of the devices given that the hole mobility of PVK (2.5×10^{-6} cm^2 V^{-1} s^{-1})[11] is approximately one order of magnitude lower than the electron mobility of TPBi (3.3×10^{-5} cm^2 V^{-1} s^{-1})[12] and a substantial hole injection barrier (~1.1 eV) is present at ITO/PVK interface (Fig. 4a)[4]. Note that replacement of poly-TPD with NiO_x leads to inferior device performance (Supplementary Fig. 16), likely due to the interfacial traps at the NiO_x/PVK interface, leading to imbalanced carrier injection[13]. We further discover that spin-coating a thin layer of PVP (~1.5 mg/ml) on PVK improves the device performance but a higher concentration of PVP deteriorates both external quantum efficiency (EQE) and color purity (Supplementary Fig. 17). Although PVP is unfavorable from the perspective of achieving ultranarrow emission because its strong coordination with the perovskite tends to increase inhomogeneity during vapor treatment, we nevertheless observed a slightly higher external PL quantum yield (PLQY) for the vapor-treated perovskite on ITO/PVP than for that on ITO/PVK (Supplementary Fig. 18). This result suggests that the PVP that is dissolved into the perovskite precursors during film fabrication and accumulates near the film surface could passivate defects owing to its strong coordination with perovskite precursors, thereby increasing the external PLQY of thin films and EQE of the corresponding PeLEDs.

Supplementary Note 4. Outcoupling efficiency simulation

Post-treated perovskite thin films exhibit spatially isolated grains, which have previously been suggested to enhance light outcoupling in PeLEDs[8,14-16]. However, a quantitative relationship between film morphology and light outcoupling in blue PeLEDs remains underexplored. To address this, we employed 3D finite-difference time-domain (FDTD) simulation[16] (Supplementary Fig. 19) based on the SEM images of post-treated 35%Cl perovskite thin films (Supplementary Fig. 20). Atomic force microscopy (AFM) height profiles (Supplementary Fig. 21) confirm comparable film thicknesses (~50 nm) across samples with varying film coverage. The simulation indicates that light outcoupling efficiency increases from 21±2% to 31±4% as film coverage decreases from ~61% to ~18% (Fig. 4b). Note that the outcoupling efficiency should be underestimated as photon recycling is not included in the simulation owing to its complexity. Photon recycling has been widely reported in halide perovskites[17] and has been well recognized to improve light extraction in PeLEDs through repeated re-absorption and re-emission of photons[18]. Despite that, our optical simulation qualitatively correlates well with the experimental trends. The average EQE improves from 16.3% to 21.8% as the film coverage is reduced from ~61% to ~31% (Supplementary Fig. 22a). However, further reducing the film coverage to ~18% leads to decreased EQE of 19.8%, likely due to increased current leakage in over diluted perovskite thin films.

Supplementary Note 5. Discussion of our strategy and the stability of the device

Our mechanism also offers insight into the broader EL linewidth observed in devices fabricated from concentrated perovskite precursors (Supplementary Fig. 22). In such films, densely packed grains (Supplementary Fig. 20e and 21c) likely suppress vapor-assisted ion diffusion and crystallization, leading to increased inhomogeneity, broader emission linewidths (Supplementary Fig. 31b), and lower PLQY (Supplementary Fig. 32a). Although energy funneling in inhomogeneous films can partially narrow emission by concentrating radiative recombination in lower bandgap domains, this process is often incomplete especially when the energetic offsets are small or the domains are spatially disconnected. In contrast, narrow spectral linewidths in homogeneous thin films reflect intrinsic energetic order rather than a redistribution of emission.

Beyond high color purity, our perovskite thin films and LEDs exhibit good spectral stability under continuous intense laser illumination (Supplementary Fig. 33b) and high current density operation (Supplementary Fig. 23c). This is particularly significant given the widespread concern over spectral instability in mixed-halide perovskite under optical and electrical stress, primarily attributed to halide segregation. Our results demonstrate that this issue could be substantially mitigated by improving the horizontal and vertical homogeneity of perovskite thin films. In contrast, pronounced spectral shifts are observed in thin films and devices with greater inhomogeneity (Supplementary Fig. 33a, 33c, 34c, and 22i), highlighting the critical role of film homogeneity in achieving a stable spectrum in mixed halide perovskite. Nevertheless, despite the spectral stability, the overall operational lifetime of the devices remains comparable to other high-performance blue PeLEDs. This observation highlights the multifaceted nature of degradation in blue PeLEDs where failure mechanisms such as ion migration-induced interface degradation, electrochemical reactions, and Joule heating may dominate even when the spectral stability issue is substantially improved. In addition to the decrease in EL under continuous operation, we observed that the driving voltage initially decreased and then gradually increased under constant current density in both green and blue PeLEDs (Supplementary Fig. 23d-f, 27b, 28c-d, 29b). This evolution of the driving voltage suggests a complicated electrical degradation process within the device and further indicates that the green and blue PeLEDs likely share a similar degradation mechanism. Previous work[19] attributed this behavior to ion migration into the electron-transporting layer, thereby degrading its electron-transporting capability. As a consequence, the device resistance increases over time and a higher driving voltage is required to maintain a constant current. In our system, the precursor ratio of AX (CsBr, RbBr, FABr) to PbX ($PbBr_2$, $PbCl_2$) is 1.5. Therefore, a fraction of the excess halide salts may not be incorporated into the perovskite lattice but instead remain at the film surface and grain boundaries. These excess halide species are more susceptible to electric field-driven migration during device operation, which may accelerate interfacial degradation and shorten the operational lifetime. This motivates future work to improve device stability including the development of strategies to reduce the quantity of excess halides and introducing further barrier layers to suppress ion migration into adjacent junctional layers. The improvements introduced in this work, combined with such further strategies, pave a promising path for maintaining high performance and stability in blue PeLEDs.

Supplementary Table 1. Estimated attenuation length of X-ray at different incident angles

Incident angle	Attenuation length, nm
0.04°	2.52
0.08°	2.65
0.12°	2.93
0.16°	3.48
0.20°	5.16
0.24°	26.32
0.28°	54.07
0.32°	74.65
0.36°	92.33
0.40°	108.92

Notes: The attenuation length is the depth into the material measured along the surface normal where the intensity of X-rays falls to 1/e of its value at the surface. The attenuation length is estimated using the website https://henke.lbl.gov/optical_constants/atten2.html. For simplicity, the chemical formula was set as $CsPbBr_2Cl$. Its density of 4.66 g cm^{-3} was extracted from ref. [20]. Photon energy was set as 10 keV.

Supplementary Table 2. Summary of the state-of-the-art blue PeLEDs

**EL peak** nm	**FWHM**		**Peak EQE** %	**Peak luminance** cd m^{-2}	**Reference**
	nm	meV*			
488	28	146	14.71	5,015	21
488	29	151	14.29	6,111	22
488	21	109	23.6	~1,000	23
487	~21	110	14.82	3,131	24
486	23	121	16.07	2,541	25
485	23	121	11.0	9,040	26
483	24	127	21.4	1,981	27
483	25	133	18.65	1,147	28
483	14.7	78	24.6	7,997	This work
482	15.8	84	19.28	1,457	29
482	15	80	4.13	3,180	30
480	17	91	26.4	1,601	31
480	21	113	18	~3,000	32
479	20	108	12.3	~400	33
478	21	114	20.9	~400	23
478	21.1	114	21.9	640	34
477	16	87	12.0	1,867	35
477	18	98	11	2,180	14
476	15.0	82	19.1	3,305	This work
475	17	93	24.3	1,175	31
475	18	99	10.1	14,000	36
474	-	-	13.2	~700	27
470	20	112	10.1	11,610	37
470	17	95	21.3	635	31
469	15	84	1.00	666	30
468	22	124	11.87	710	38
468	15	85	4.6	1,680	39
468	-	-	6.6	582	40
468	21	119	15.0	~100	23
467	18	102	5.5	~340	14
465	23	132	10	576	32
465	14.0	80	10.6	1,713	This work
465	17	97	16.7	430	31
464	17	98	9.8	2,180	41
464	-	-	7.3	~500	27
463	>20	>115	12	300	42
461	15.4	90	3.05	2,707	43
460	17	100	12	255	31
460	15	88	0.23	171	30

Note: the FWHM in meV was estimated from the data in nm.

Supplementary Table 3. Summary of the state-of-the-art blue QLEDs

EL peak<br>nm	FWHM		Peak EQE<br>%	Peak luminance<br>cd/m^2	Reference
	nm	meV			
482	22	117	20.4	-	44
479	23	124	21.9	~50,000	45
478	19	103	19.7	-	44
474	~24	~132	20.6	24,020	46
474	28	154	21.4	~20,000	47
472	34	189	16.2	14,100	48
471	20	112	20.8	~10,000	44
468	20	113	19.8	4,890	49
467	20	114	20.1	-	44
464	27	155	21.35	13,150	50
461	20	117	18.59	44,080	51
460	35	205	20.2	~88,900	52
460	17	100	24.7	36,850	53
458	26	154	23	17,641	54
458	25	148	18.6	10,398	55
455	20	120	10.7	-	56
452	22	133	18	3,520	57

Supplementary Table 4. Summary of the state-of-the-art blue OLEDs

EL peak<br>nm	FWHM		Peak EQE<br>%	Peak luminance<br>cd/m^2	Reference
	nm	meV			
480	26	140	37.6	13,850	58
480	27	145	22.9	-	59
478	25	140	21.0	-	60
473	19	105	28.5	-	61
473	19	105	38.8	-	62
472	23	128	30.9	-	63
472	17	95	29.4	-	64
470	19	106	41	-	65
470	28	157	30.8	-	66
469	26	146	33.7	-	67
469	18	101	34.4	-	68
466	21	120	24.2	-	69

References

1 Mishra, B. K. & Sathyamurthy, N. π−π Interaction in Pyridine. *J. Phys. Chem. A* **109**, 6–8 (2004).

2 Malinkiewicz, O. *et al.* Perovskite solar cells employing organic charge-transport layers. *Nat. Photon.* **8**, 128–132 (2013).

3 Abbaszadeh, D. *et al.* Elimination of charge carrier trapping in diluted semiconductors. *Nat. Mater.* **15**, 628–633 (2016).

4 Mao, J. *et al.* All-Perovskite Emission Architecture for White Light-Emitting Diodes. *ACS Nano* **12**, 10486–10492 (2018).

5 Stranks, S. D. *et al.* Recombination Kinetics in Organic-Inorganic Perovskites: Excitons, Free Charge, and Subgap States. *Phys. Rev. Appl.* **2**, 034007 (2014).
6 Wolff, C. M. *et al.* Orders of Recombination in Complete Perovskite Solar Cells - Linking Time-Resolved and Steady-State Measurements. *Adv. Energy Mater.* **11**, 2101823 (2021).
7 Kirchartz, T., Márquez, J. A., Stolterfoht, M. & Unold, T. Photoluminescence-Based Characterization of Halide Perovskites for Photovoltaics. *Adv. Energy Mater.* **10**, 1904134 (2020).
8 Li, M. *et al.* Acceleration of radiative recombination for efficient perovskite LEDs. *Nature* **630**, 631–635 (2024).
9 Feldmann, S. *et al.* Photodoping through local charge carrier accumulation in alloyed hybrid perovskites for highly efficient luminescence. *Nat. Photon.* **14**, 123–128 (2019).
10 D'Andrade, B. W. & Forrest, S. R. White Organic Light-Emitting Devices for Solid-State Lighting. *Adv. Mater.* **16**, 1585–1595 (2004).
11 Dai, X. *et al.* Solution-processed, high-performance light-emitting diodes based on quantum dots. *Nature* **515**, 96–99 (2014).
12 Wu, I. W., Wang, P.-S., Tseng, W.-H., Chang, J.-H. & Wu, C.-I. Correlations of impedance–voltage characteristics and carrier mobility in organic light emitting diodes. *Org. Electron.* **13**, 13–17 (2012).
13 Li, Z. C. *et al.* Charge injection engineering at organic/inorganic heterointerfaces for high-efficiency and fast-response perovskite light-emitting diodes. *Nat. Commun.* **14**, 6441 (2023).
14 Karlsson, M. *et al.* Mixed halide perovskites for spectrally stable and high-efficiency blue light-emitting diodes. *Nat. Commun.* **12**, 361 (2021).
15 Richter, J. M. *et al.* Enhancing photoluminescence yields in lead halide perovskites by photon recycling and light out-coupling. *Nat. Commun.* **7**, 13941 (2016).
16 Cao, Y. *et al.* Perovskite light-emitting diodes based on spontaneously formed submicrometre-scale structures. *Nature* **562**, 249–253 (2018).
17 Pazos-Outon, L. M. *et al.* Photon recycling in lead iodide perovskite solar cells. *Science* **351**, 1430–1433 (2016).
18 Cho, C. *et al.* The role of photon recycling in perovskite light-emitting diodes. *Nat. Commun.* **11**, 611 (2020).
19 Cheng, T. *et al.* Ion Migration-Induced Degradation and Efficiency Roll-off in Quasi-2D Perovskite Light-Emitting Diodes. *ACS Appl. Mater. Interface* **12**, 33004–33013 (2020).
20 Balvanz, A. *et al.* Unveiling the Monoclinic Phase in $CsPbBr_{3-x}Cl_x$ Perovskite Crystals, Phase Transition Suppression and High Energy Resolution γ-Ray Detection. *J. Am. Chem. Soc.* **146**, 31836–31848 (2024).
21 Liu, B. *et al.* Lattice strain modulation toward efficient blue perovskite light-emitting diodes. *Sci. Adv.* **8**, eabq0138 (2022).
22 Zhang, F. J. *et al.* Engineering of Hole Transporting Interface by Incorporating the Atomic-Precision Ag Nanoclusters for High-Efficiency Blue Perovskite Light-Emitting Diodes. *Nano Lett.* **23**, 1582–1590 (2023).
23 Zhang, K. *et al.* Nondestructive halide exchange via SN2-like mechanism for efficient blue perovskite light-emitting diodes. *Nat. Commun.* **15**, 10621 (2024).
24 Shen, Y. *et al.* Multifunctional Crystal Regulation Enables Efficient and Stable Sky-Blue Perovskite Light-Emitting Diodes. *Adv. Funct. Mater.* **32**, 2206574 (2022).

25 Chu, Z. M. *et al.* Blue light-emitting diodes based on quasi-two-dimensional perovskite with efficient charge injection and optimized phase distribution via an alkali metal salt. *Nat. Electron.* **6**, 360–369 (2023).
26 Wang, Q. *et al.* Efficient sky-blue perovskite light-emitting diodes via photoluminescence enhancement. *Nat. Commun.* **10**, 5633 (2019).
27 Yuan, S. *et al.* Efficient blue electroluminescence from reduced-dimensional perovskites. *Nat. Photon.* **18**, 425–431 (2024).
28 Zhou, W. *et al.* Manipulating Ionic Behavior with Bifunctional Additives for Efficient Sky-Blue Perovskite Light-Emitting Diodes. *Adv. Funct. Mater.* **33**, 2301425 (2023).
29 Liu, A. *et al.* Optimizing Perovskite Surfaces to Enhance Post-Treatment for Efficient Blue Mixed-Halide Perovskite Light-emitting Diodes. *Adv. Mater.* **37**, e2414788 (2024).
30 Cheng, L. *et al.* Halide Homogenization for High-Performance Blue Perovskite Electroluminescence. *Research* **2020**, 9017871 (2020).
31 Gao, Y. *et al.* Highly efficient blue light-emitting diodes based on mixed-halide perovskites with reduced chlorine defects. *Sci. Adv.* **10**, eado5645 (2024).
32 Jiang, Y. Z. *et al.* Synthesis-on-substrate of quantum dot solids. *Nature* **612**, 679–684 (2022).
33 Dong, Y. T. *et al.* Bipolar-shell resurfacing for blue LEDs based on strongly confined perovskite quantum dots. *Nat. Nanotechnol.* **15**, 668–674 (2020).
34 Qi, H. *et al.* Homogenizing Energy Landscape for Efficient and Spectrally Stable Blue Perovskite Light-Emitting Diodes. *Adv. Mater.* **36**, e2409319 (2024).
35 Luo, X. Y. *et al.* Effects of local compositional heterogeneity in mixed halide perovskites on blue electroluminescence. *Matter* **7**, 1054-1070 (2024).
36 Yang, Y. *et al.* Highly Efficient Pure-Blue Light-Emitting Diodes Based on Rubidium and Chlorine Alloyed Metal Halide Perovskite. *Adv. Mater.* **33**, e2100783 (2021).
37 Liu, A. Q. *et al.* High Color-Purity and Efficient Pure-Blue Perovskite Light-Emitting Diodes Based on Strongly Confined Monodispersed Quantum Dots. *Nano Lett.* **23**, 2405–2411 (2023).
38 Liu, Y. J. *et al.* A Multifunctional Additive Strategy Enables Efficient Pure-Blue Perovskite Light-Emitting Diodes. *Adv. Mater.* **35**, e2302161 (2023).
39 Tong, Y. F. *et al.* In Situ Halide Exchange of Cesium Lead Halide Perovskites for Blue Light-Emitting Diodes. *Adv. Mater.* **35**, e2207111 (2023).
40 Zhao, S. *et al.* Postdeposition Halide Exchange for Achieving Deep-Blue Perovskite Light-Emitting Diodes: The Role of the Organic Cations in the Chloride Source. *Small Methods* **8**, e2300572 (2023).
41 Jiang, Y. Z. *et al.* Unraveling Size-Dependent Ion-Migration for Stable Mixed-Halide Perovskite Light-Emitting Diodes. *Adv. Mater.* **35**, e2304094 (2023).
42 Wang, Y. K. *et al.* Self-assembled monolayer-based blue perovskite LEDs. *Sci. Adv.* **9**, eadh2140 (2023).
43 Lee, S. *et al.* Brightening deep-blue perovskite light-emitting diodes: A path to Rec. 2020. *Sci. Adv.* **10**, eadn8465 (2024).
44 Chen, X. T. *et al.* Blue light-emitting diodes based on colloidal quantum dots with reduced surface-bulk coupling. *Nat. Commun.* **14**, 284 (2023).
45 Deng, Y. Z. *et al.* Solution-processed green and blue quantum-dot light-emitting diodes with eliminated charge leakage. *Nat. Photon.* **16**, 505–511 (2022).
46 Wang, F. F. *et al.* High-Performance Blue Quantum-Dot Light-Emitting Diodes by Alleviating Electron Trapping. *Adv. Opt. Mater.* **10**, 2200319 (2022).
47 Zhang, H., Chen, S. & Sun, X. W. Efficient Red/Green/Blue Tandem Quantum-Dot Light-Emitting Diodes with External Quantum Efficiency Exceeding 21. *ACS Nano* **12**, 697–704 (2018).

48 Lin, Q. *et al.* Nonblinking Quantum-Dot-Based Blue Light-Emitting Diodes with High Efficiency and a Balanced Charge-Injection Process. *ACS Photon.* **5**, 939–946 (2018).
49 Wang, L. *et al.* Blue Quantum Dot Light-Emitting Diodes with High Electroluminescent Efficiency. *ACS Appl. Mater. Interfaces* **9**, 38755–38760 (2017).
50 Li, C. G. *et al.* Low-Temperature Cross-Linked Hole Transport Layer for High-Performance Blue Quantum-Dot Light-Emitting Diodes. *Nano Lett.* **24**, 5729–5736 (2024).
51 Zhang, X., Li, D., Zhang, Z., Liu, H. & Wang, S. Constructing Effective Hole Transport Channels in Cross-Linked Hole Transport Layer by Stacking Discotic Molecules for High Performance Deep Blue QLEDs. *Adv. Sci.* **9**, 2200450 (2022).
52 Kim, T. *et al.* Efficient and stable blue quantum dot light-emitting diode. *Nature* **586**, 385–389 (2020).
53 Wu, Q. Q. *et al.* Homogeneous ZnSeTeS quantum dots for efficient and stable pure-blue LEDs. *Nature* **639**, 633–638 (2025).
54 Zhang, W. J. *et al.* Stable and efficient pure blue quantum-dot LEDs enabled by inserting an anti-oxidation layer. *Nat. Commun.* **15**, 783 (2024).
55 Lee, S.-H. *et al.* Heterostructural tailoring of blue ZnSeTe quantum dots toward high-color purity and high-efficiency electroluminescence. *Chem. Eng. J.* **429**, 132464 (2022).
56 Yang, Y. *et al.* High-efficiency light-emitting devices based on quantum dots with tailored nanostructures. *Nat. Photon.* **9**, 259–266 (2015).
57 Bi, Y. H. *et al.* Reducing Emission Linewidth of Pure-Blue ZnSeTe Quantum Dots through Shell Engineering toward High Color Purity Light-Emitting Diodes. *Small* **19**, e2303247 (2023).
58 Xing, L. J. *et al.* Highly efficient pure-blue organic light-emitting diodes based on rationally designed heterocyclic phenophosphazinine-containing emitters. *Nat. Commun.* **15**, 6175 (2024).
59 Oda, S. *et al.* One-Shot Synthesis of Expanded Heterohelicene Exhibiting Narrowband Thermally Activated Delayed Fluorescence. *J. Am. Chem. Soc.* **144**, 21426–21427 (2022).
60 Nagata, M. *et al.* Fused-Nonacyclic Multi-Resonance Delayed Fluorescence Emitter Based on Ladder-Thiaborin Exhibiting Narrowband Sky-Blue Emission with Accelerated Reverse Intersystem Crossing. *Angew. Chem. Int. Ed.* **60**, 20280–20285 (2021).
61 Stavrou, K., Franca, L. G., Danos, A. & Monkman, A. P. Key requirements for ultraefficient sensitization in hyperfluorescence organic light-emitting diodes. *Nat. Photon.* **18**, 554–561 (2024).
62 Braveenth, R. *et al.* Achieving Narrow FWHM and High EQE Over 38% in Blue OLEDs Using Rigid Heteroatom-Based Deep Blue TADF Sensitized Host. *Adv. Funct. Mater.* **31**, 2105805 (2021).
63 Lee, H. L. *et al.* Multiple-Resonance Extension and Spin-Vibronic-Coupling-Based Narrowband Blue Organic Fluorescence Emitters with Over 30% Quantum Efficiency. *Adv. Mater.* **34**, e2202464 (2022).
64 Park, I. S., Yang, M. L., Shibata, H., Amanokura, N. & Yasuda, T. Achieving Ultimate Narrowband and Ultrapure Blue Organic Light-Emitting Diodes Based on Polycyclo-Heteraborin Multi-Resonance Delayed-Fluorescence Emitters. *Adv. Mater.* **34**, 2107951 (2022).
65 Chan, C.-Y. *et al.* Stable pure-blue hyperfluorescence organic light-emitting diodes with high-efficiency and narrow emission. *Nat. Photon.* **15**, 203–207 (2021).

66 Xiao, X., Lei, B. W., Wu, D. & Bin, Z. Y. "Medium-ring" strategy enables high-performance narrowband pure-blue multi-resonance emitters: boost provided by a unique perpendicular geometry. *Chem. Commun.* **59**, 6556–6559 (2023).

67 Naveen, K. R., Oh, J. H., Lee, H. S. & Kwon, J. H. Tailoring Extremely Narrow FWHM in Hypsochromic and Bathochromic Shift of Polycyclo-Heteraborin MR-TADF Materials for High-Performance OLEDs. *Angew. Chem. Int. Ed.* **62**, e202306768 (2023).

68 Kondo, Y. *et al.* Narrowband deep-blue organic light-emitting diode featuring an organoboron-based emitter. *Nat. Photon.* **13**, 678–682 (2019).

69 Liu, G., Sasabe, H., Kumada, K., Arai, H. & Kido, J. Nonbonding/Bonding Molecular Orbital Regulation of Nitrogen-Boron-Oxygen-embedded Blue/Green Multiresonant TADF Emitters with High Efficiency and Color Purity. *Chem. Eur. J.* **28**, e202201605 (2022).
